# Molecular characterization of *Bathymodiolus* mussels and gill symbionts associated with chemosynthetic habitats from the U.S. Atlantic margin

**DOI:** 10.1371/journal.pone.0211616

**Published:** 2019-03-14

**Authors:** D. Katharine Coykendall, Robert Scott Cornman, Nancy G. Prouty, Sandra Brooke, Amanda W. J. Demopoulos, Cheryl L. Morrison

**Affiliations:** 1 US Geological Survey -Leetown Science Center, Kearneysville, West Virginia, United States of America; 2 US Geological Survey—Fort Collins Science Center, Fort Collins, Colorado, United States of America; 3 US Geological Survey—Pacific Coastal and Marine Science Center, Santa Cruz, California, United States of America; 4 Florida State University, Coastal and Marine Laboratory, St. Teresa, Florida, United States of America; 5 US Geological Survey—Wetland and Aquatic Research Center, Gainesville, Florida, United States of America; Museum National d'Histoire Naturelle, FRANCE

## Abstract

Mussels of the genus *Bathymodiolus* are among the most widespread colonizers of hydrothermal vent and cold seep environments, sustained by endosymbiosis with chemosynthetic bacteria. Presumed species of *Bathymodiolus* are abundant at newly discovered cold seeps on the Mid-Atlantic continental slope, however morphological taxonomy is challenging, and their phylogenetic affinities remain unestablished. Here we used mitochondrial sequence to classify species found at three seep sites (Baltimore Canyon seep (BCS; ~400m); Norfolk Canyon seep (NCS; ~1520m); and Chincoteague Island seep (CTS; ~1000m)). Mitochondrial COI (N = 162) and ND4 (N = 39) data suggest that *Bathymodiolus childressi* predominates at these sites, although single *B*. *mauritanicus* and *B*. *heckerae* individuals were detected. As previous work had suggested that methanotrophic and thiotrophic interactions can both occur at a site, and within an individual mussel, we investigated the symbiont communities in gill tissues of a subset of mussels from BCS and NCS. We constructed metabarcode libraries with four different primer sets spanning the 16S gene. A methanotrophic phylotype dominated all gill microbial samples from BCS, but sulfur-oxidizing Campylobacterota were represented by a notable minority of sequences from NCS. The methanotroph phylotype shared a clade with globally distributed *Bathymodiolus spp*. symbionts from methane seeps and hydrothermal vents. Two distinct Campylobacterota phylotypes were prevalent in NCS samples, one of which shares a clade with Campylobacterota associated with *B*. *childressi* from the Gulf of Mexico and the other with Campylobacterota associated with other deep-sea fauna. Variation in chemosynthetic symbiont communities among sites and individuals has important ecological and geochemical implications and suggests shifting reliance on methanotrophy. Continued characterization of symbionts from cold seeps will provide a greater understanding of the ecology of these unique environments as well and their geochemical footprint in elemental cycling and energy flux.

## Introduction

### Distribution and ecology of Atlantic bathymodiolins

Benthic communities dependent on bacterial chemosynthesis are known to arise around a variety of geochemical and biological sources, including hydrothermal vents [1], hydrocarbon seeps [2], hypoxic sediments [3], wood [4], and whale falls [5]. Dominant fauna in these communities engage in symbioses with chemoautotrophic microbes. Deep-sea chemosynthetic communities separated by tens to hundreds of kilometers may share conspecifics and congenerics, but the fauna that rely on chemoautotrophic microbes for nutrition have yet to be discovered outside of reducing habitats. These oases provide a natural laboratory for investigating how the dynamics of symbiosis affect megafaunal community assembly. Bathymodiolin mussels, a sub-family within Mytilidae and endemic to chemosynthetic habitats, are of special interest, in part because they colonize and can dominate both hydrothermal vents and hydrocarbon seeps due to varied symbiont utilization by the hosts.

**Fig 1:**
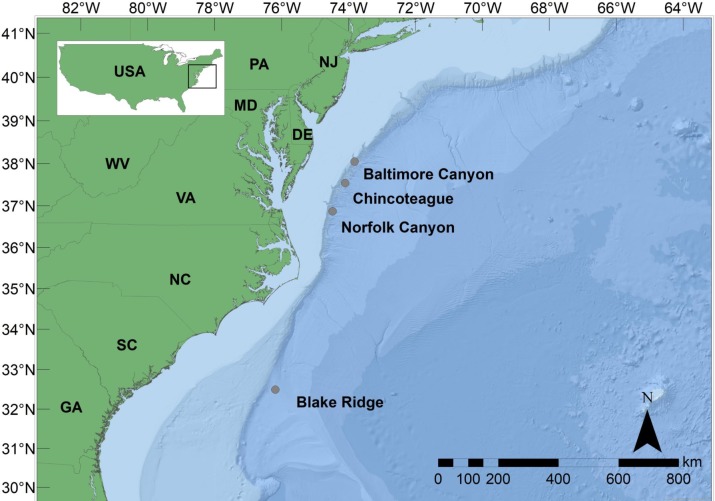
Map of sampling locations of *Bathymodiolus* spp. in this study.

Prior to 2012, only one hydrocarbon seep on the Atlantic margin of North America was known to support a chemosynthetic biological community, though many occur in the GOM [6]. Blake Ridge Diapir (BRD, 2155m) is a site off the coast of South Carolina containing a biological chemosynthetic community first discovered in 1995 [7] and later determined to be dominated by the bathymodiolin species, *Bathymodiolus heckerae* [8, 9]. In 2012, venting and associated chemosynthetic communities were found at Cape Fear Diapir, dominated by vesicomyid clams and bacterial mats [10] and lacking bathymodiolin mussels altogether. More recent surveys of hydrocarbon seepage along the Atlantic margin of North America [10, 11] have documented hundreds of potential seeps, and remotely operated vehicle (ROV) sampling expeditions have confirmed and sampled chemosynthetic fauna at several of these newly discovered sites. A seep community near Baltimore Canyon (BCS), first detected by towed camera in 1982 [12], was verified in 2012 at ~400m depth. A second, deeper seep community near Norfolk Canyon (NCS) was discovered in 2013 at ~1520m depth [13, 14]. Most recently, a seep near Chincoteague Island (CTS) was discovered at approximately ~1000 m depth [15] (Fig 1). Three additional seep communities have been discovered and explored in the northeastern U.S. (NEUS) near Nygren and Veatch canyons off the coast of New England [16]. Like chemosynthetic communities in the Gulf of Mexico (GOM) [17], the Barbados Accretionary Prism (BAP) [18, 19], and the three Mid-Atlantic seep sites (MAS: BCS, NCS, CTS), the chemosynthetic communities observed from the NEUS were dominated by bathymodiolin mussels. Vestimentiferan tubeworms and vesicomyid clams were conspicuously absent from NEUS and MAS sites, though present at similar depths within many other Atlantic and GOM seep communities [9, 10, 20, 21].

Given the wide geographic and bathymetric distributions of Atlantic bathymodiolins [22] there are several species that could inhabit the MAS sites. *Bathymodiolus heckerae* at the BRD is the closest in geographic proximity, though its known depth range is deeper than the MAS sites. In fact, BCS is the shallowest of all the Atlantic seep chemosynthetic communities so far reported. Both NCS and CTS are within the depth range of all Atlantic bathymodiolins. *Bathymodiolus childressi* has the greatest depth range of the Atlantic species (525–2284m) [23]. The closest *B*. *childressi* assemblages to the MAS occur in the Caribbean off Trinidad and Tobago [19] and throughout the GOM [2, 22, 24]. Furthermore, *B*. *childressi* larvae have been recovered from surface water plankton tows in the GOM [25], and Lagrangian transport models predict that GOM larvae can disperse to Mid-Atlantic waters [26]. Some mussels from the BAP share genetic affinity with *B*. *mauritanicus* [22, 27–29], a species found in the Gulf of Cadiz [27] and West Africa [6, 30], but their morphological characteristics are *B*. *childressi*-like, *B*. *mauritanicus*-like, or intermediate [22]. Other mussels nearby are *B*. *boomerang* [22, 31]. If other bathymodiolin species have similar larval durations and spawning behaviors as *B*. *childressi*, then the MAS mussels could potentially be any of the known GOM, BRD, or Caribbean species, or possibly a new species altogether.

Bathymodiolin species prove difficult to identify solely based on morphology, as evidenced by a study where 12–33% of initial morphological field identifications of GOM mussels were incorrect when verified with molecular markers [24]. Molecular data have also indicated the presence of species complexes in which separate species may be conspecifics [9, 29, 32, 33]. Therefore, augmenting morphological analyses of complex and plastic bathymodiolin taxonomy to perform species identifications at newly-discovered chemosynthetic communities, like the MAS sites, necessitate molecular methods.

### Bathymodiolin symbiont diversity

Initial studies of mussels from chemosynthetic environments concluded that each species harbored a single methanotrophic endosymbiont (e.g. [2, 34, 35]), a single thiotrophic endosymbiont (e.g [36]), or both (e.g. [37]) contained in bacteriocytes within gill tissue [2, 34]. These functionally divergent symbionts typically fell into two distinct clades of Gammaproteobacteria [38]. However, more recent studies have demonstrated that the symbiotic and nutritional profiles of bathymodiolin mussels are more varied and complex [39–41]. For example, filamentous, thiotrophic, “Epsilonproteobacterial” (since re-classified as Phylum Epsilonbacteraeota, then amended to Campylobacterota [42, 43]) epibionts were isolated from gill tissue of *Bathymodiolus childressi* in addition to its known methanotrophic Gammaproteobacteria endosymbiont. Furthermore, these Campylobacterota appear to be associated broadly with bathymodiolins, as metagenomic signatures of the epibionts were found in nucleotide sequences from four out of eight bathymodiolin species surveyed [44]. The discovery of thiotrophic Campylobacterota epibionts associated with *B*. *childressi*, a species presumed to rely strictly on methanotrophic Gammaproteobacterial endosymbionts for nutritional input, and the fact that not all species of *Bathymodiolus* contain Campylobacterota epibionts [44], illustrates the potential plasticity and adaptability of the hosts to a chemically dynamic environment, and that there is still much to discover with regard to host-symbiont relationships in bathymodiolins.

Sulfur isotope signatures (δ^34^S) from gill tissue of the recently-discovered MAS mussels suggested their dominant nutritional source is methane, but with a reliance of 16% (NCS) and 14% (BCS) on hydrogen sulfide (H_2_S) as an energy source [13], indicating the MAS mussels demonstrate thiotrophic and methanotrophic nutritional modes, much like most other Atlantic bathymodiolins. However, the microbial source of the chemical signatures remains unknown. Therefore, to more fully understand the ecology of the MAS mussels and their role in geochemical cycling, characterizing the symbiont pool within the gill tissue in MAS hosts found at different seep sites is essential.

In this study, we present the first genetic analysis of bathymodiolin mussels from BCS, NCS, and CTS and their gill symbionts from BCS and NCS. We sequenced two mitochondrial genes (Cytochrome Oxidase I (COI) and NADH dehydrogenase subunit 4 (ND4)) to verify the identities of MAS mussels. Second, we used the mitochondrial sequence data to examine biodiversity and to reconstruct phylogeographic relationships among these and other Atlantic bathymodiolin species. Additionally, we characterized the bacterial community found in the gill tissue from NCS and BCS mussels via high-throughput 16S metabarcoding and four overlapping primer sets to cover the majority of the 16S gene. Lastly, we verified dominant microbial phylotypes with Sanger sequencing. The metagenomic sequencing approach of the mussel gill microbiome has the potential to detect rarer bacterial phylotypes than traditional Sanger sequencing and cloning. Results were interpreted with respect to key issues in taxonomy, distributions, and ecology of *Bathymodiolus*.

## Methods

### Sample collection

In 2012, 2013, and 2017, bathymodiolin mussels were collected from methane seeps found near Baltimore Canyon (N = 48, sampled at depths between 353–507 m), Norfolk Canyon (N = 92, 1487–1612 m) and a site near Chincoteague Island (N = 35, 925-1037m), using the ROVs *Kraken* (University of Connecticut) in 2012, the *Jason II* (Woods Hole Oceanographic Institute) in 2013, and the *Global Explorer* (National Oceanographic and Atmospheric Administration) in 2017 (Table 1; S1 Table). Adductor or mantle tissue was taken from the mussels for host characterization to avoid co-extraction of symbiotic bacterial DNA and gill tissue for microbiome characterization. All tissue was preserved in 95% molecular grade ethanol. Because the CTS samples were obtained more recently, none were included in the 16S symbiont metabarcoding study. Permissions were obtained to collect specimens in the study regions from the NOAA National Marine Fisheries Service as scientific research in accordance with the definitions and guidance at 50 CFR Sections 600.10 and 600.745(a). The proposed activities were not subject to fishing regulations at 50 CFR 622 or other regulations developed in accordance with the Manguson-Stevens Fishery Conservation and Management Act and did not involve endangered or protected species.

**Table 1:** Sampling information of Mid-Atlantic bathymodiolin mussels from three seep sites.

Site	Dive/Station #	Date	Depth (m)	Lat (N)	Lon (W)	Sample Size
BCS	B08	8/27/2012	412–454	38°03'04	73°49'19	15
	B14	9/7/2012	407–507	38°02'57	73°49'20	9
	B689	5/16/2013	353–441	38°02'53	73°49'19	20
	GEX06-075	5/11/2017	393	38°02'50	73°49'22	4
CTS	GEX04-032	5/9/2017	1037	37°32'33	74°06'06	9
	GEX04-035	5/9/2017	1024	37°32'29	74°06'07	10
	GEX05-053	5/10/2017	925	37°32'29	74°09'16	10
	GEX05-069	5/10/2017	938	37°31'13	74°09'09	6
NCS	N01	5/8/2013	1519–1612	36°52'05	74°29'14	37
	J2683	5/9/2013	1421–1564	36°52'11	74°29'18	28
	GEX03-009	5/8/2017	1482	36°52'17	74°28'34	7
	GEX03-011	5/8/2017	1491	36°52'17	74°28'35	7
	GEX03-023	5/8/2017	1494	36°52'20	74°49'28	11

BCS = Baltimore Canyon Seep, NCS = Norfolk Canyon Seep, CTS = Chincoteague Seep. Latitudes (lat (N)), longitudes (lon (W)), and depth ranges from years 2012–2013 are from [45].

### Bathymodiolin molecular methods

Genomic DNA was extracted and purified via the Puregene Tissue Protocol (Qiagen), doubling the volume of all reagents. The DNA was eluted in 100 μl of molecular grade water. For most samples, a portion of the mitochondrial COI was amplified via PCR using the primers HCO2198 and LCO1490 [46] or BathCOIF and BathCOIR [22]. The primers ArgBL (L-10421 in [47] and NAD2H (NAD2 in [48] were used to amplify a portion of tRNA methionine, tRNA valine, and the 5’ portion of the ND4 gene [49] (see S2A and S2B Table for PCR conditions). PCR purification, cycle sequencing reactions, clean-up, and Sanger sequencing was performed as in [50].

### Bathymodiolin mussel data analyses

DNA sequences were edited using Sequencher 5.2.2 (Genecodes) and aligned in MEGA 7.0.26 [51] using the ClustalW algorithm [52] and translated into amino acids (excluding tRNA-Met and tRNA-Val from the ND4 sequences) using the invertebrate mtDNA translation table to ensure no stop codons were present. Then, sequences were divided into three partitions corresponding to the 1st, 2nd, and 3rd codon positions for phylogenetic tree generation (Open Science Framework, DOI 10.17605/OSF.IO/GCWT2, File 1).

Bayesian phylogenetic analyses were performed for the concatenated mtDNA data set (S3 Table) and symbiont data sets (below) with MrBayes v3.2.6 x64 [53] on XSEDE using the CIPRES Science Gateway V. 3.3 (https://www.phylo.org/; [54]). Four independent runs of six chains of Markov Chain Monte Carlo sampling were run for a total of 20^7^−50^7^ generations with settings to match the most appropriate model of sequence evolution for the dataset estimated in MEGA. The “sumt” command was used to generate a consensus tree file, which was visualized using FigTreev1.4.2 (http://tree.bio.ed.ac.uk/software/figtree/). All runs were analyzed in Tracerv1.6 [55] to assess convergence (Open Science Framework, DOI 10.17605/OSF.IO/GCWT2, File 1).

Genetic diversity indices were estimated from the mitochondrial data using DnaSP 5.10 [56]. Both datasets contained MAS sequences, *B*. *childressi* from several GOM sites, *Bathymodiolus cf mauritanicus* from WAF seeps and *B*. *sp B* BAP (COI only), and *B*. *mauritanicus* from GOC (S4 Table). Hierarchical genetic structuring between different groupings within the data was estimated using Analysis of Molecular Variance (AMOVA) as implemented in the poppr and ade4 packages of R. Groups were defined hierarchically as species (*B*. *childressi* and *B*. *mauritanicus*), then regions, then sites within *B*. *childressi* (S4 Table). The ten sites from the GOM were defined as in [24]. For the Region/Site AMOVA, only sites that contained ten or more individuals were included. A “quasieuclid” correction method was applied to the distance matrix calculated from the raw pairwise distances (ade4 package, https://cran.r-project.org/web/packages/ade4/ade4.pdf). The strictest “farthest neighbor” algorithm was used to merge clusters based on maximum distance between points in either cluster. If five percent or more of nucleotides were missing at any given site, that position was excluded from the analysis. Significance testing was performed via 10000 random permutations of the data. Median joining haplotype networks [57] were created for COI and ND4 using PopART (Population Analysis with Reticulate Trees [58]), with ε = 0. If more than five percent of the sites across all sequences contained missing data, they were masked.

### Gill microbiome molecular methods

#### 16S metabarcode libraries

Ten mussels, three from a single NCS dive site and seven from three BCS dive sites, were selected for 16S metabarcoding of their gill microbiomes (S1 Table, ‘16S’). Mussel samples from CTS were obtained late in the study, so were not included in the microbial community analysis. DNA was extracted from gill tissue using the same protocol described above for mussels. To ensure that symbionts would be recovered from sequencing efforts, four overlapping primer sets that each amplify approximately 460–500 bp were used to capture the majority of the 16S gene (~1242 bp; S5 Table). Primer set 1 was equivalent to the universal primers used in the Illumina protocol (www.illumina.com, 16S Metagenomic Sequencing Library Preparation, Part# 15044223, revA). Three additional primer sets were designed along the 16S gene from 124 symbionts sequences from several *Bathymodiolus* species (S6 Table). The sequences were aligned via the SSU-ALIGN alignment pipeline (55) which utilizes the CRW database (http://www.rna.ccbb.utexas.edu; [59]). All 40 libraries, (10 mussel samples × 4 primer sets) were pooled to a 4nM concentration and five percent PhiX was added as a control. The final library was diluted to 12pM and run on an Illumina MiSeq at the USGS–Leetown Science Center.

#### Generation of 16S sequences from overlapping amplicons

We built full length consensus sequence models that spanned all four primer sets by stringently mapping reads from all primer sets to selected full-length 16S references to generate novel consensus sequences. Appropriate references were identified by aligning 50 randomly selected reads from ps1 and ps2 from a single sample (MASM22) to the GenBank nucleotide database (nt) with BLAST (S7 Table). This was done with two primer sets to compare the consistency of phylogenetic placements between gene fragments. Both primer sets had a large portion of reads that had best BLAST matches to a consistent set of closely related Gammaproteobacteria methanotrophs isolated from species of *Bathymodiolus*. One of these, Genbank accession AM236329, was chosen as a mapping reference. Reads from all four primer sets were mapped to this accession with Bowtie2 [60] v. 2.2.8. Reads mapping at less than 97% identity or with more than two indels were filtered from the resulting alignment file. The majority-rule consensus sequence (i.e. without ambiguity characters) was then generated with SAMtools v.1.3 [61].

In a subset of our samples, a notable portion of the reads matched to GenBank accessions from Campylobacterota, though the best BLAST matches were more variable across primer sets, making a suitable template for consensus generation difficult to choose. We therefore used an explicitly phylogenetic assessment rather than choosing a high-scoring match, by constructing *de novo* consensus sequences of the Campylobacterota reads from MASM22. These were mapped to known Campylobacterota sequences and the majority-rule consensus sequence for each group of different Campylobacterota found in our data was generated as described above. A neighbor-joining phylogeny was constructed in MEGA 7 [62] using the consensus sequences of the 50 most abundant Campylobacterota groups (based on read count) and a broad set of reference 16S sequences representing known bathymodiolin symbionts (S1 Fig). Trees for ps1 and ps2 amplicons were consistent, showing two clusters of consensus groups surrounding Genbank accessions KU573880, an uncultured Campylobacterota bacterium clone from *Bathymodiolus sp*. collected from off the coast of Pakistan [44] and FM994669, an uncultured Campylobacterota bacterium from the gills of *Pectinodonta* sp., a limpet host found on sunken wood [63]. These two accessions were therefore selected as references for mapping reads from all four primer sets, after culling the reads used to generate the methanotroph consensus. Alignments less than 97% identity or with more than two indels were again filtered and consensus sequences generated as above.

A final mapping of all reads simultaneously to the three consensus-sequence phylotypes was performed to assess their relative abundance by primer set. Reads that failed to map stringently (≥97% identity, no more than two indel positions, and at least 400-bp in length) were considered unclassified. The final Bowtie2 alignment was loaded in the alignment viewer Tablet [64] to calculate mismatch frequencies by position and confirm that no high-frequency alternative alleles were present that were discordant with the inferred consensus.

The accuracy of these consensus sequences was further confirmed by Sanger sequencing of targeted amplicons. These were generated with specific forward primers and a common reverse primer (BathySymR: 5’-AAGGGCCATGATGACTTGAC-3’). Primer BathyMethF (TCAATTGGGAGGAAAACAGG) targeted the Gammaproteobacteria methanotroph (“Phylotype M” hereafter), primer BathyCampKUF (TATACCAAGATTATGACGGTAG) targeted the Campylobacterota similar to KU573880 (“Phylotype C1”) and primer BathyCampFMF (TGTTAGAAGATAATGACGGTAT) targeted the Campylobacterota similar to FM994669 (“Phylotype C2”). The primers were tested in a subset of mussels: MASM5, MASM22, MASM30, MAS538, MAS562, and MASM34. The PCRs recipe and conditions used for amplifications are listed in S2A and S2B Table. PCR purification, Sanger sequencing, sequence editing and alignment were performed as above.

#### Phylogenetic analysis of symbiont 16S sequences

Both Bayesian and maximum likelihood (ML) phylogenetic analyses of the dominant symbionts were performed. A phylogeny of endosymbiont Gammaproteobacteria was constructed with 36 16S sequences from methanotrophic endosymbionts from bathymodiolins and Phylotype M. The Bayesian phylogenetic analyses were run as above, specifying the best model of sequence evolution (K2+G+I) determined in MEGA 6.06. The significance of each clade in the ML trees was determined with 500 bootstrap replications. A second phylogenetic analysis was performed for Campylobacterota, including phylotypes C1 and C2 as well as 69 sequences used in the phylogenetic analysis in [44] (see S8 Table for NCBI accession numbers). Trees were estimated with the best-fit model of sequence evolution (K2 + G) as well as the general time reversible (GTR) + G + I (nst = 6, rates = invgamma), for comparison with the analysis in [44]. Batch input files for both phylogenies can be found at Open Science Framework, DOI 10.17605/OSF.IO/GCWT2, File 2. Further details regarding metadata can be found at [65].

#### OTU generation and taxonomy per primer set

Reads from each primer set were run independently through the Mothur 1.39.5 pipeline [66, 67], mostly following the Mothur MiSeq SOP (https://www.mothur.org/wiki/MiSeq_SOP; accessed December 2016 –August 2018; [66]). Fastq files from both reads per primer set were merged and sequences trimmed, processed, aligned in Mothur version 1.39.5, and clustered into operational taxonomic units (OTUs; cut-off of 0.03), and classified in Mothur version 1.38.1.1. VSEARCH [68], was used to detect chimeric sequences in two ways. First, using the consensus sequences for phylotypes M, C1, and C2 as references, then in a *de novo* fashion. After the two chimeric removals, reads identified as putative chimeras were removed from downstream analyses. Taxonomic classification within the Mothur pipeline used the SILVA database (https://www.arb-silva.de; [69]), version 132 (released December 2017) as the reference database. We followed the reference curation protocol (http://blog.mothur.org/2018/01/10/SILVA-v132-reference-files/) to generate a reference database specific to each primer set region. Mothur input and output files can be found in Open Science Framework, DOI 10.17605/OSF.IO/GCWT2, File 3.

Mothur-generated OTU counts and taxonomy were imported into the Phyloseq package (v. 1.19) in R (v. 3.3.2) for diversity analysis. For each primer set, singleton OTUs were removed and counts were rarefied to the minimum sample size. Taxonomic proportions at each sampling site were also visualized hierarchically with Krona [70].

## Results

### Phylogenetic analysis of *Bathymodiolus* samples

The COI sequence alignment was 676 bp in length and included 162 MAS mussels (Genbank accession numbers MG519868-MG519983; MH723711-MH723755, S1 Table). The COI dataset contained 83 variable sites (with two sequences omitted due to missing data), three of which were predicted to result in amino acid changes. The ND4 sequence alignment was 626 bp, included 39 individuals (Genbank accession numbers MG519984-MG520022, S1 Table) and contained 146 variable sites, including 39 nonsynonymous variants. No nonsense or frameshift mutations (indicators of nuclear pseudogenes) were observed. The HRS035 ND4 sequence had three nucleotide insertions in the tRNA portion of the sequence compared to the remaining samples. The concatenated mtDNA dataset consisted of six partitions: the 1^st^, 2^nd^, and 3^rd^ nucleotide positions of codons in both genes, excluding sequence from tRNA-Val and tRNA-MetPhylogenetic analysis included 21 MAS samples with data from both loci and 35 samples with data retrieved from Genbank (S3 Table).

**Fig 2:**
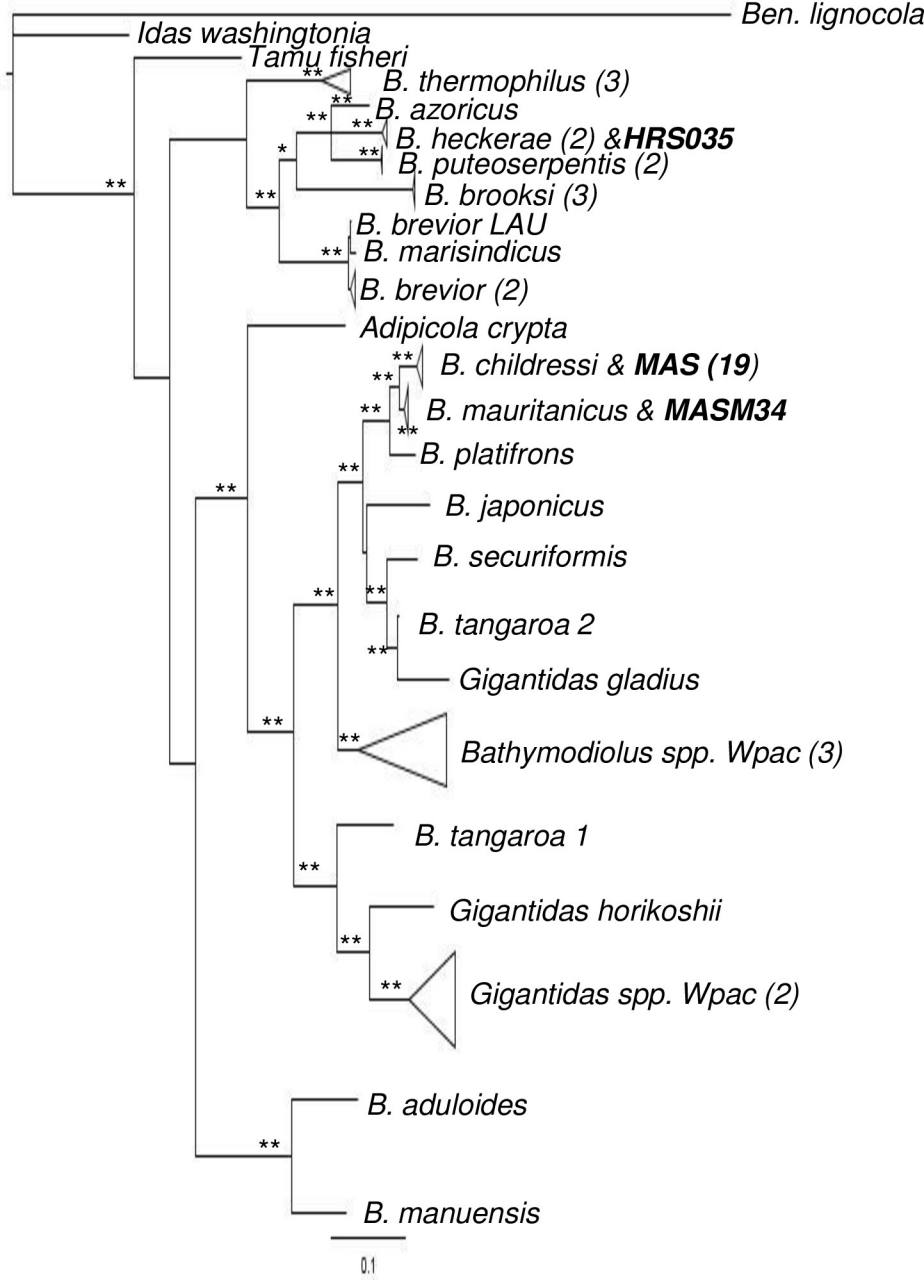
Bayesian phylogeny constructed from mitochondrial COI+ND4 including 21 mussels collected from Mid-Atlantic seep sites (MAS). Posterior probabilities above 0.90 = *; above 0.95 = **. S3 Table contains Genbank accession numbers of individuals not collected in this study.

The COI + ND4 phylogeny recovered *Bathymodiolus childressi* and *B*. *mauritanicus* as closely-related sister taxa with high statistical support. Most MAS individuals grouped with *B*. *childressi* accessions, except for MASM34 which grouped with *B*. *mauritanicus* (Fig 2; posterior probability = 1.0). There was also strong support for the “*childressi*” complex, including *B*. *platifrons*, *B*. *japonicus*, *B*. *securiformis*, *B*. *spp*. from the West Pacific, plus several *Gigantidas* species. Our phylogeny was mostly concordant with the results from [32] in which *B*. *platifrons* was the outgroup to the *B*. *childressi* + *B*. *mauritanicus* clades. Slight differences in placement of the two *B*. *tangaroa* accessions within the “*childressi*” complex were noted. The closest species outside of the “*childressi*” complex was *Adipicola crypta*, collected from the west Pacific, which was not included in the previous study. As above, HRS035 grouped with *B*. *heckerae* with high support (posterior probability = 1.0). Deeper branches in our phylogeny had low statistical support. Outside of the “childressi” complex, the *B*. *heckerae*, *B*. *brevior*, and *B*. *thermophilus* groups were similar between the two trees as well as their relationships to each other. A notable exception was the relationships between major clades within the *B*. *brooksi* group. In the former study, the *B*. *heckerae* group’s sister clade was the *B*. *brevior* group. In our phylogeny, the *B*. *brooksi* group is sister to the *B*. *heckerae* group (posterior probability = 0.94). The most basal group of the *B*. *boomerang/B*. *thermophilus* complex in [31] was the *B*. *brooksi* group, but our phylogeny puts *B*. *thermophilus* in the basal position (posterior probability = 0.98).

### Mitochondrial DNA genetic diversity

Summary statistics of nucleotide and haplotype diversity revealed similar levels of diversity at the BCS, NCS, and CTS (COI only) sites (Table 2). Haplotype diversity (*H*_*d*_), which accounts for different sample sizes, was similar among each site yet was slightly higher for ND4 (CTS excluded). For COI, the *B*. *mauritanicus* sequences included representatives from both sides of the Atlantic Ocean. Though *η*, *S*, and *h* were smaller than those from the *B*. *childressi* populations, *H*_*d*_ and *k* were comparable.

**Table 2:** Genetic diversity within different populations of *B*. *childressi* and *B*. *mauritanicus* at COI and ND4 mitochondrial genes.

Gene		Group	*N*	*nt*	*η*	*S*	*h*	*Hd*	*σ Hd*	*k*	*σk*	*π*	*Δaa*
COI	*	BCS	*46*	*344*	*16*	*14*	*17*	*0*.*84*	*0*.*0015*	*1*.*95*	*1*.*27*	*0*.*005*	*1*
		NCS	*79*	*446*	*22*	*20*	*26*	*0*.*83*	*0*.*0009*	*1*.*97*	*1*.*27*	*0*.*004*	*0*
		CTS	*35*	*355*	*13*	*12*	*14*	*0*.*85*	*0*.*0410*	*1*.*65*	*0*.*99*	*0*.*005*	*0*
		*B*. *chi* (GOM)	*106*	*331*	*31*	*29*	*35*	*0*.*83*	*0*.*0010*	*1*.*64*	*0*.*95*	*0*.*005*	*0*
		*B*. *mau* (w/MASM34)	*31*	*366*	*13*	*13*	*11*	*0*.*82*	*0*.*0025*	*1*.*81*	*1*.*15*	*0*.*005*	*0*
ND4	*	BCS	*13*	*597*	*14*	*14*	*11*	*0*.*96*	*0*.*0025*	*3*.*56*	*3*.*75*	*0*.*006*	*4*
		NCS	*21*	*596*	*12*	*12*	*14*	*0*.*93*	*0*.*0016*	*2*.*55*	*2*.*04*	*0*.*004*	*2*
	*	*B*. *chi* (GOM)	*76*	*623*	*49*	*47*	*38*	*0*.*95*	*0*.*0002*	*3*.*11*	*2*.*67*	*0*.*005*	*5*
		*B*. *mau* (w/ MASM34)	*6*	*456*	*7*	*7*	*6*	*1*.*00*	*0*.*0093*	*2*.*53*	*2*.*49*	*0*.*006*	*2*

BCS = Baltimore Canyon Seep, NCS = Norfolk Canyon Seep, CTS = Chincoteague Seep, GOM = Gulf of Mexico, MAS = Mid-Atlantic Seeps, *B*. *chi* = *Bathymodiolus childressi*, *B*. *mau* = *Bathymodiolus mauritanicus*

*These populations do not include MASM34.

*nt* = number of nucleotides used in the analyses, *η* = number of mutations *S* = number of segregating sites, *h* = number of haplotypes, *H*_*d*_ = haplotype diversity, *σH*_*d*_ = variance of *H*_*d*_, k = number of pairwise differences *σ*_*k*_ = stochastic and sampling variance of *k*, assuming no recombination, *π* = nucleotide diversity, Δaa = number of nucleotide changes that result in an amino acid change

**Fig 3:**
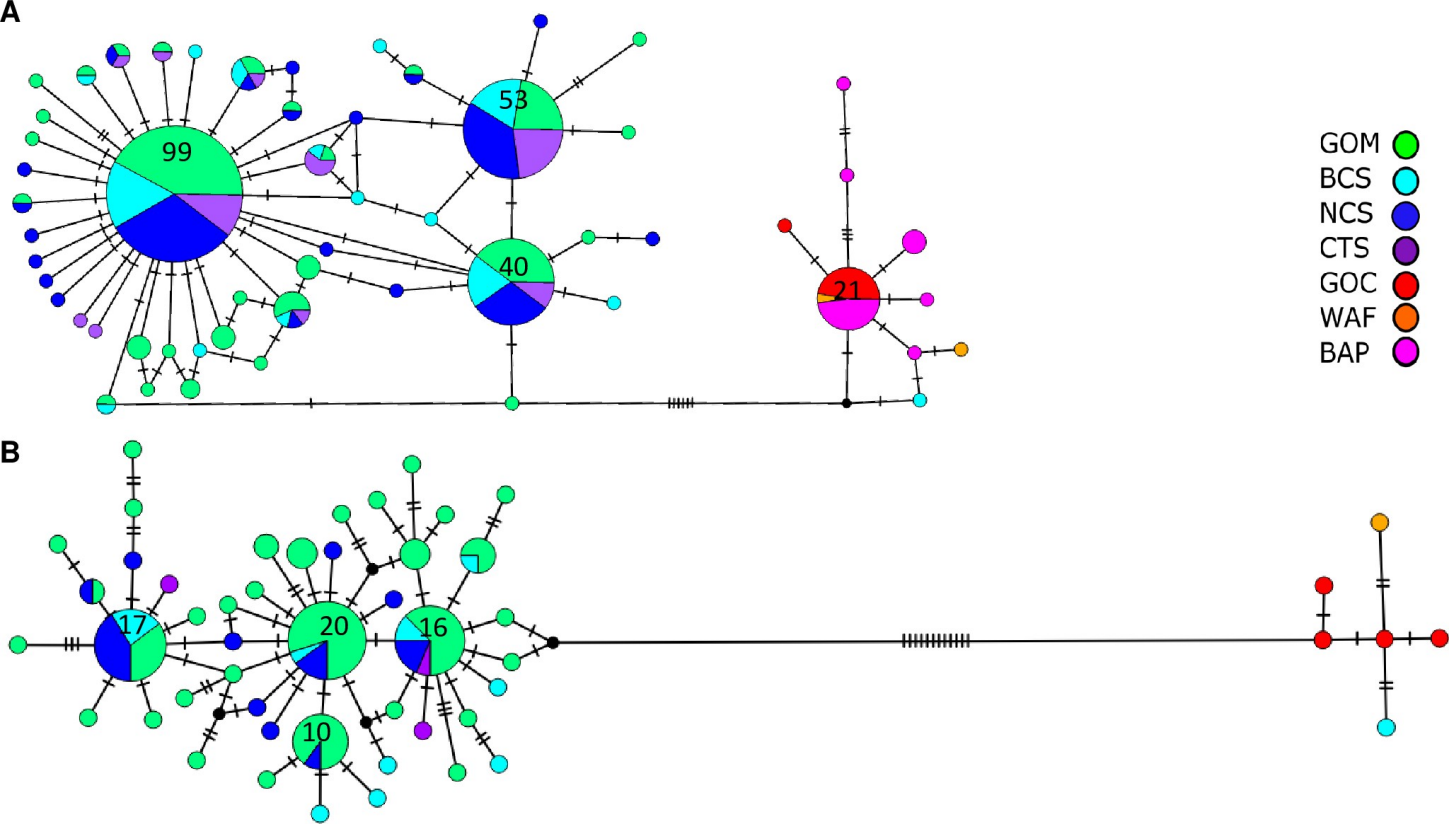
Minimum spanning networks created from A) COI and B) ND4 sequences generated from Mid-Atlantic seep mussels. Each circle represents a unique haplotype. Size is proportional to the number of mussels sharing the haplotype. Sample sizes ≥ 10 are reported inside the circles. Hash marks are mutational steps between haplotypes. GOM = *B*. *childressi* from several Gulf of Mexico sample sites, BCS = Baltimore Canyon Seep, NCS = Norfolk Canyon Seep, CTS = Chincoteague Seep, GOC = *B*. *mauritanicus* from Gulf of Cadiz, WAF = *B*. *cf*. *mauritanicus* from West Africa, BAP = *B*. *sp B* from the Barbados Accretionary Prism. See Table 1, S1, and S4 Tables for sample information.

Due to their close genetic relationship, sequences from *B*. *mauritanicus* and *B*. *childressi* were included in a minimum spanning COI haplotype network analysis (N = 297; Fig 3A, see S1 and S4 Tables). The network contained 56 unique haplotypes. Two main haplotype groups were separated by six mutational steps. The larger group contained all the *B*. *childressi* plus most of the MAS mussels, with three common haplotypes accounting for 64.6% of the sequences in the network. These common haplotypes were shared among the GOM, CTS, BCS, and NCS samples. Another grouping of haplotypes was separated from the main *B*. *childressi* haplotype groups by at least ten mutational steps. This smaller group was comprised of sequences from *B*. *cf mauritanicus*, *B*. *mauritanicus*, *B*. *sp B* BAP and MAS34 from BCS, and contained nine unique haplotypes. The MASM34 was a single mutational step away from a *B*. *sp B* BAP haplotype and was at least ten mutational steps from the nearest MAS haplotype.

An ND4 haplotype network incorporating 119 sequences (51 unique haplotypes) from *B*. *mauritanicus* and *B*. *childressi* was broadly concordant with the COI network (Fig 3B; see S1 and S4 Tables). The MASM34 sample grouped with *B*. *mauritanicus* individuals that were separated by at least 16 mutational steps from the larger *B*. *childressi* haplotype group containing the remaining MAS mussel haplotypes. Each of the six haplotypes within the *B*. *mauritanicus* group was found in a single individual. At least 21 mutational steps separated MASM34 from the next closest MAS haplotype. In the *B*. *childressi* group, the four most common haplotypes accounted for 52.9% of the ND4 sequences and were shared between the GOM and at least one of the MAS sites.

An AMOVA analysis was performed on the COI and ND4 data from MAS mussels and *Bathymodiolus childressi* and *B*. *mauritanicus* individuals from other studies (S4 Table). When considering only species level differentiation, the results were significant for both COI and ND4, with 83.4–85.5% of the variation within the data ascribed to between-species partitioning (Table 3). For the site by region AMOVAs, the COI analysis included five GOM sites and two MAS sites, based on a sample size of ten or greater (S1 and S4 Tables). For the ND4 AMOVA, four GOM and two MAS sites were included. These tests were not significant at the *p* = 0.05 level for either gene between regions and between sites within regions (Table 3). In both cases, 96–99% of the variation observed was attributed to within-site variation.

**Table 3:** Analysis of Molecular Variation (AMOVA) between *Bathymodiolus childressi* and *B*. *mauritanicus* species and within and between regions for *B*. *childressi*.

COI	*df*	*Φ*	*σ*	*%*	*p-val*
Between Species	1	0.834	4.626	82.973	1.0E-04
w/n Species	242		0.949	17.028	
Total	243		5.575	100	
Between Regions	1	0.010	0.016	1.609	0.053
w/n Region, btwn Sites	6	-0.0014	-0.003	-0.328	0.586
w/n Site	231	0.0084	0.972	98.719	0.260
Total	238		0.9849	100	
ND4	*df*	*Φ*	*σ*	*%*	*p-val*
Between Species	1	0.855	6.959	85.484	1.0E-04
w/n Species	117		1.182	14.516	
Total	118		8.141	100	
Between Regions	1	0.035	0.043	3.526	0.069
w/n Region, btwn Sites	4	0.004	0.004	0.339	0.375
w/n Site	80	0.039	1.169	96.135	0.119
Total	85		1.216	100	* *

Analyses were executed in poppr and ade4 packages of R. See S4 Table for definition of Regions and Sites and [23] for explanation of GOM sites. df = degrees of freedom, Φ = degree of differentiation analogous to F-statistics (68), σ = variance for each hierarchical level, % = percent of total variance partitioned to each level, p-val = p-value estimated from 10,000 permutations

### Gill microbiome

#### Sequencing QC

The total number of reads that passed filter and were successfully demultiplexed was 17,408,059. The overall per-base error rate estimated from the PhiX control spike was 2.45%. The average distribution of reads per sample was 2.21%, which is close to the targeted value of 2.5%. Libraries for primer set 3 performed poorly, with three samples falling below 1% of total reads. One sample from ps2 fell below one percent. The smallest libraries, MAS538_2 (ps2) and M36_3 (ps3), were excluded from downstream analyses. MAS538_4 and MAS562_4, both from ps4, resulted in much higher numbers of reads than the expected 2.5%. MAS538_4 accounted for 14.7% of passed read pairs (S2 Fig). Fastq sequences were deposited into the sequence read archive (SRA) in Genbank under BioProject PRJNA401268 with the following BioSample accessions: SAMN07601752–61.

#### Phylogenetic analysis of the consensus phylotypes

Full length 16S consensus sequences, phylotypes “M”, “C1”, and “C2”, were generated by stringently mapping reads to accessions AM236329, KU573880, and FM994669, respectively, which were identified as closely related by BLAST searches and phylogenetic analysis (see Methods for details). This approach assumes that each consensus sequence represents a single bacterial species and that full-length accessions can be identified that are similar enough to allow stringent read mapping. The accuracy of the reconstructed, consensus sequences was confirmed by targeted PCR and Sanger sequencing. Phylotype M was successfully sequenced in seven of the eight mussels screened, with 100% sequence identity (Genbank accession numbers MH984855-59; 719 bp). The sample MASM5 failed to amplify for all three primer sets and a subsequent PCR with universal primers, suggesting sample degradation in storage. Phylotypes C1 and C2 were successfully sequenced in MASM22, MASM30, and MAS538 (Genbank accession numbers MH938809-11; MH939150-52). Neither C1 nor C2 amplified in the remaining individuals, MASM34, MASM45, MAS100, and MAS562, which is concordant with our metabarcoding results (see below). All Campylobacterota-positive samples amplified both Campylobacterota phylotypes. The Sanger sequence of C1 had 99% sequence identity with the consensus model (713 aligned nucleotides; 81% coverage). The Sanger sequence of C2 also had 99% sequence identity with the consensus model (710 nucleotides; 58% query coverage). The two Campylobacterota consensus models shared 94% identity with each other.

**Fig 4:**
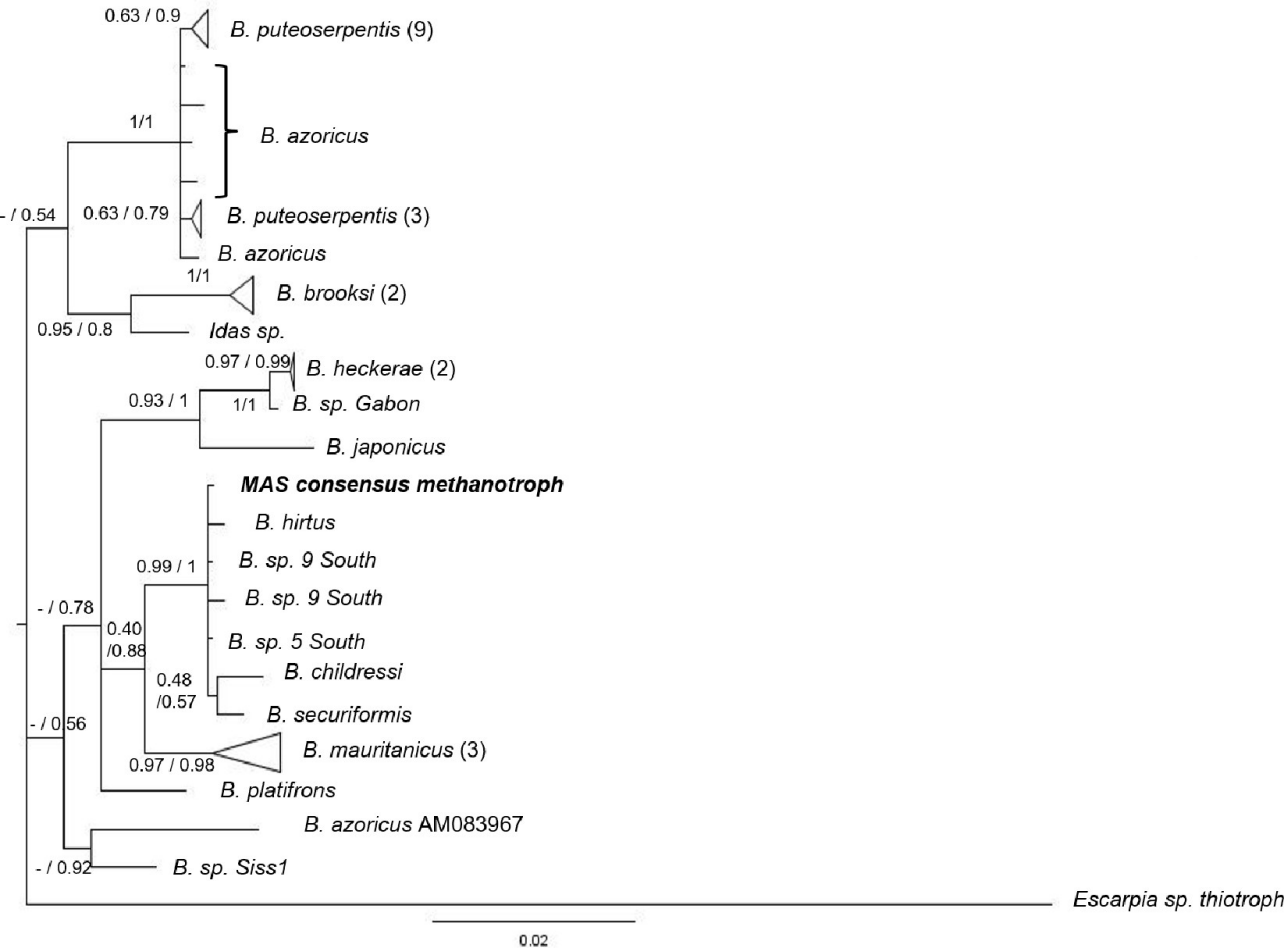
16S Bayesian phylogram based upon 16S sequences from known endosymbionts from bathymodiolins and other deep-sea hosts. The nodes are labeled with the ML probabilities based on 500 bootstrap replicates before the slash and Bayesian posterior probabilities after the slash. If the node placement did not agree between the two trees, a “-” is indicated before the slash. The branch tips are labeled with the name of the host species. If more than one sequence from that host is represented in that clade, the sample size is in parentheses after the name. A Gammaproteobacteria thiotroph from a hydrocarbon seep tubeworm, *Escarpia sp*. (JF969172), was used as an outgroup. Our consensus sequence, M, is in bold. S8 Table contains Genbank accession numbers of all individuals in the tree.

**Fig 5:**
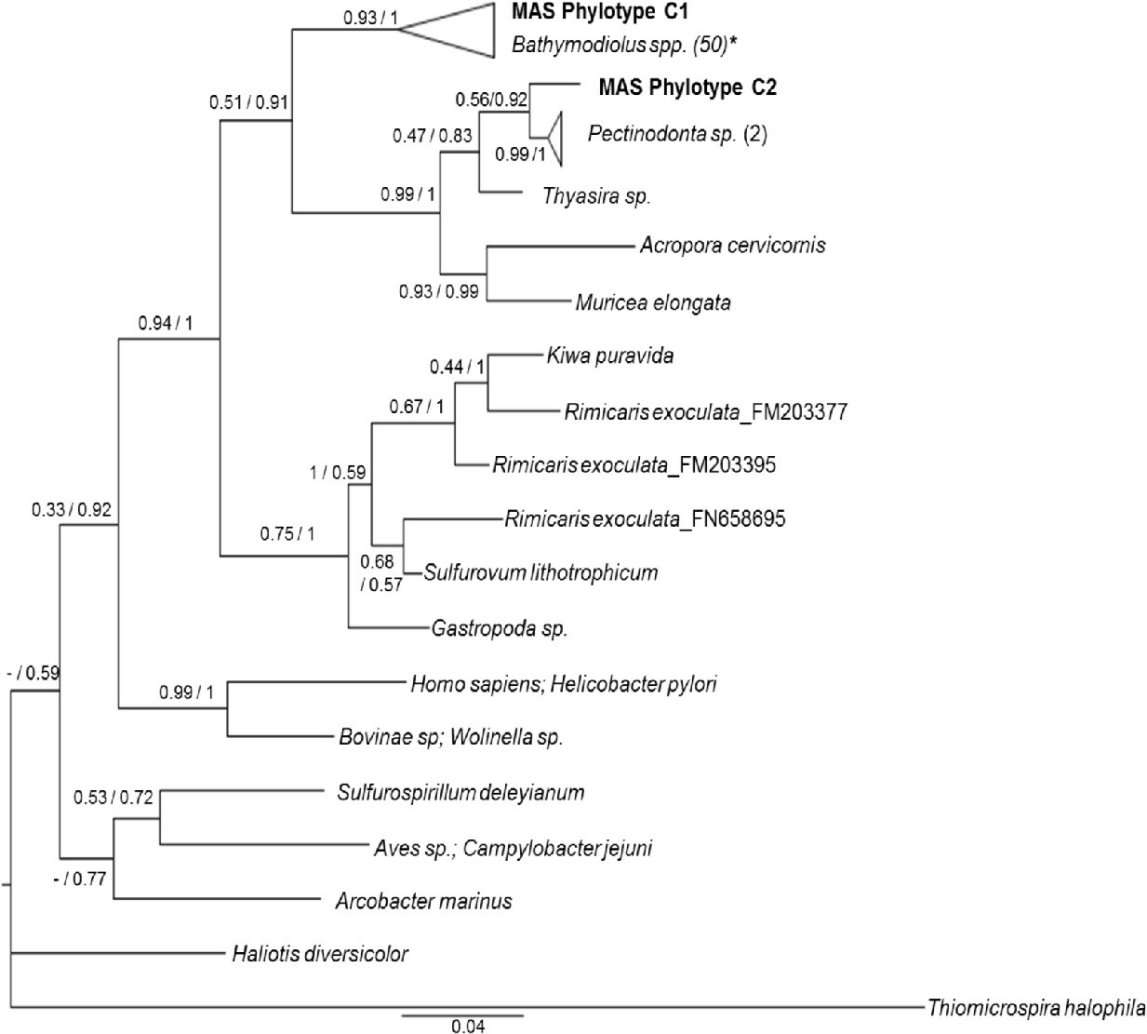
16S Bayesian phylogram based upon 16S sequences for Campylobacterota. The nodes are labeled with maximum likelihood bootstrap probabilities based on 500 bootstrap replicates before the slash and Bayesian posterior probabilities after the slash. If the node placement did not agree between the two trees, a “-” is indicated before the slash. The branch tips are labeled with the name of the host species. If more than one sequence from that host is represented, the samples size is in parentheses after the name. In cases where the host is not a marine organism, the host and symbiont are both listed, separated by a semi-colon. *Sulfurovum lithotrophicum*, *Arcobacter marinus*, and *Sulfurospirillum deleyianum* were collected from sediment. S8 Table contains Genbank accession numbers of individuals in the trees. Phylotypes C1 and C2 are in bold. *The clade including C1 also includes symbionts isolated from *Bathymodiolus azoricus*, *B*. *childressi*, *B*. *manuensis*, *B*. *mauritanicus*, *B*. *sp*. 9 South, and *B*. *sp*. Pakistan.

The Bayesian and ML phylogenies (Figs 4 and 5; S8 Table) containing our full length reconstructed phylotypes were mostly concordant. Phylotype M falls within a well-supported clade containing endosymbionts from *Bathymodiolus childressi*, two undescribed species from the southern MAR (*B*. *sp* 5 South and *B*. *sp* 9 South), and two species of *Bathymodiolus* from off the coast of Japan. In both trees, endosymbionts isolated from *B*. *mauritanicus* are the immediate outgroups of this clade. Across both phylogenies, shallower relationships tended to exhibit higher statistical support than deeper nodes. Discrepancies between the trees occurred with the placement of the clade containing *Idas sp*. and *B*. *brooksi* symbionts, a clade containing an aberrant *B*. *azoricus* sequence (AM083967), *B*. *sp*. Siss1, and *B*. *platifrons*.

Bayesian and ML trees agreed about the placement of C1 and C2. Phylotype C1 fell into a clade of 50 Campylobacterota accessions isolated from several species of *Bathymodiolus*, including *B*. *childressi*, (KU573846-80; KU644646-60: [44]). Phylotype C2 had a sister relationship to a symbiont from a deep-sea gastropod (*Pectinodonta* sp.). Related taxa included symbionts from a cold seep clam (*Thyasira* sp.), and two coral species (*Acropora cervicornis* and *Muricea elongata*). Among the more basal clades, discrepancies occurred between our trees and the Campylobacterota phylogeny in [44], regarding the placement and relationships of the most basal taxa. However, only one accession per genus was reported, such that we were not able to completely recreate their phylogenetic analysis with our added sequences. Adopting the same evolutionary model (GTR+G+I) used by [44] did not alter the structure of either of our phylogenies.

#### OTU generation and taxonomy assessed per primer set

**Fig 6:**
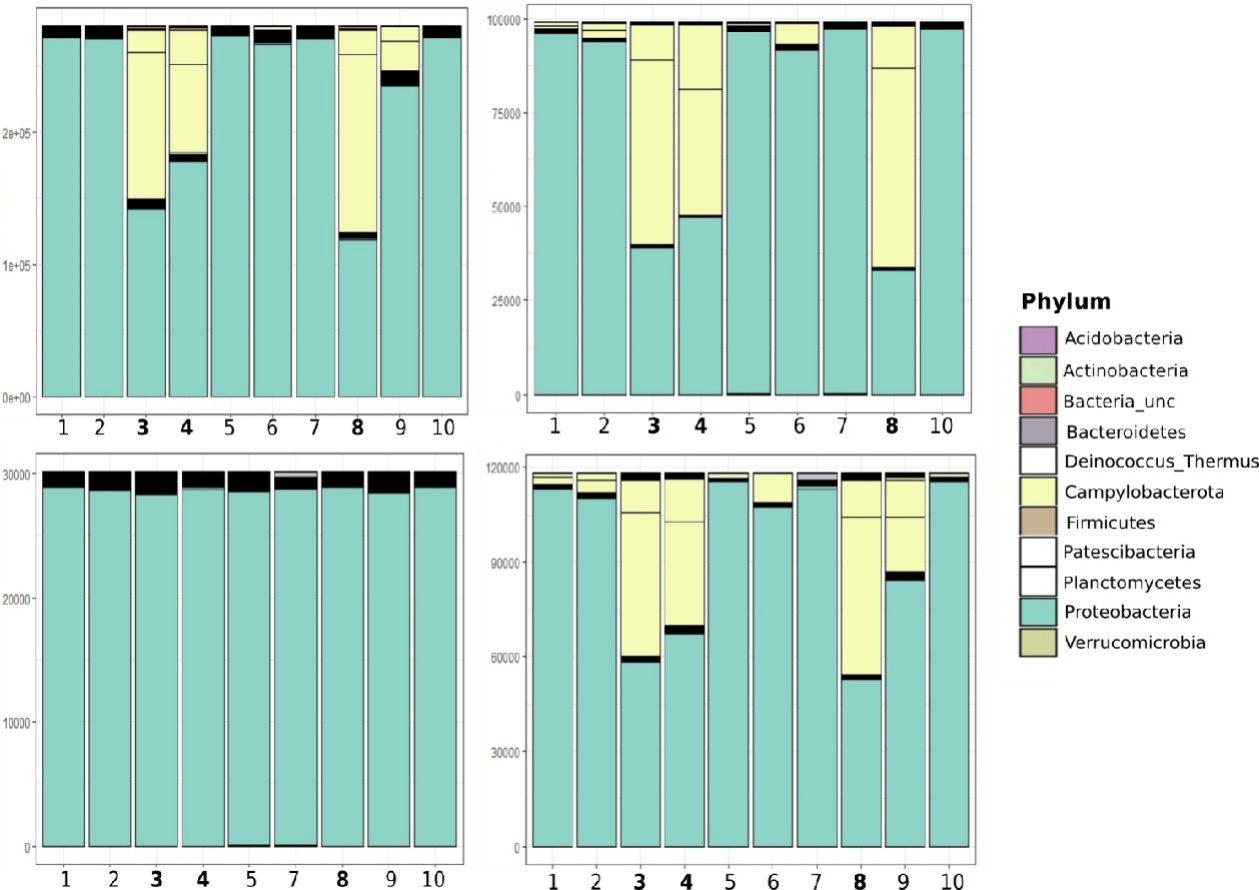
Phylum-level diversity per sample recovered by four primer sets. Phyla abundances as assigned with Mothur and the SILVA v132 reference database. Each panel represents a different primer set (ps1-ps4). The y-axis represents number of OTUs x1000. 1 = MAS100, 2 = MAS109, 3 = MASM22, 4 = MASM30, 5 = MASM34, 6 = MASM36, 7 = MASM45, 8 = MASM5, 9 = MAS538, 10 = MAS562. NCS samples are shown in bold on the x axis.

**Fig 7:**
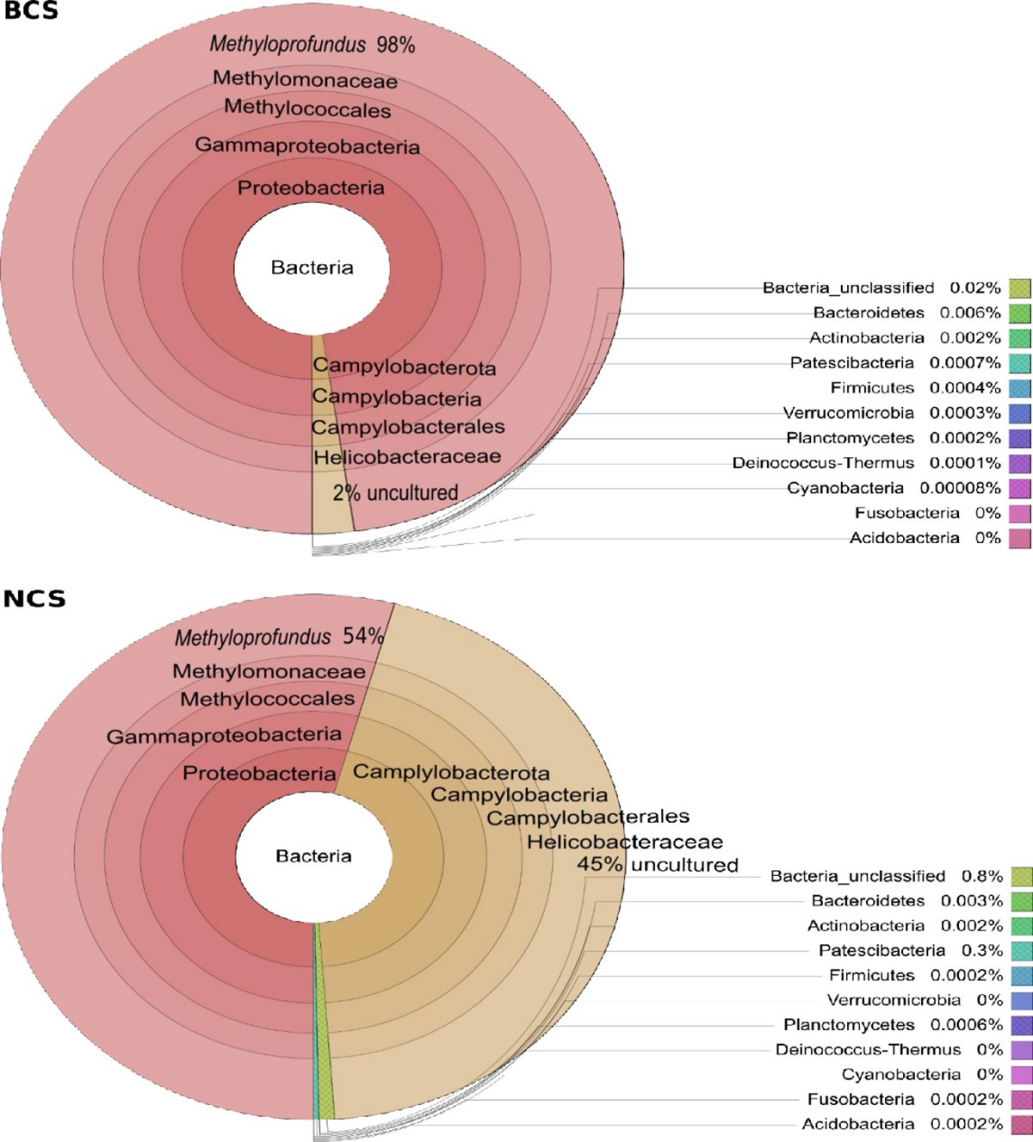
Hierarchical distribution of bacterial diversity at each site. The top circle represents Baltimore Canyon site (BCS) and the bottom circle represents Norfolk Canyon site (NCS). The taxonomic hierarchy proceeds outward. Primer set 1 (ps1) is shown. The results from ps2 and ps4 were similar, but ps3 lacked Campylobacterota due to substantial primer mismatches.

The reads from each primer set per sample were taken through the Mothur pipeline to access the performance of each primer set and examine the abundances of taxa per sample and site. The quality control, chimera removal, and singleton trimming resulted in a 30.7–72.5% reduction of reads depending on the primer set (S9 Table). In concordance with our findings above, the majority of the resultant OTUs from each primer set were assigned to Phylum Proteobacteria, with a notable minority assigned to Phylum Campylobacterota (Silva v132 assigned as Epsilonproteobacteraeota, since renamed as Campylobacterota [43]; Fig 6). Within Proteobacteria, the most abundant Class was Gammaproteobacteria (Fig 7; S10 Table). Those OTUs were assigned the Family Methylomonaceae within the Order Methylococcales. This taxonomic assignment accounted for 78.5–99.7% of the total reads across all samples and primer sets (S10 Table). Helicobacteraceae (Campylobacterota: Camplyobacteria; Campylobacterales) was the second most abundant Family and accounted for 13.20–21.4% of the reads for primer sets ps1,2, and 4, but were observed in all the NCS samples and one BCS sample. Although BCS samples all had at least trace amounts of Campylobacterota from the ps1, ps2, and ps4 primers, MAS538 (Fig 6, number 9) was the only BCS sample with substantial proportions, whereas comparable profiles were found in all NCS samples. The distribution of the two most abundant families were different between the two sampling sites, with 98% of the OTUs assigned to the genus *Methyloprofundus* (Family Methylomonaceae) in the BCS ps1 samples, but only 54% assigning to *Methyloprofundus* in NCS samples (Fig 7). Forty-five percent of OTUs from NCS ps1 were assigned to Helicobacteraceae.

The OTU diversity measured with Mothur and Phyloseq was similar to the proportion of reads mapping to the three consensus phylotypes from each sample and supported the consistency of the reconstructed full-length phylotypes with the Mothur analysis (S3 Fig; S11 Table). The known bathymodiolin endosymbiotic thiotrophs (e.g. Gammaproteobacteria from *B*. *mauritanicus* [41, 71] or *B*. *heckerae* [72, 73], belong to the Thioglobaceae Family (Order Thiomicrospirales). Reads from all four primer sets were assigned to Thiomicrospirales, but at low relative abundances (<0.0001–0.0014%; S10 Table). We observed only trace amounts of Thiomicrospirales in sample MASM34, which clustered with *B*. *mauritanicus*/*B*. *sp* B in phylogenetic analyses. While *B*. *mauritanicus* from GOC has been shown to harbor both methanotrophic and thiotrophic Gammaproteobacteria endosymbionts, the symbiotic profile of *B*. *sp* B BAP is not known.

The communities identified with each primer set were broadly similar but with some notable differences. Primer set 4 yielded higher Campylobacterota abundances in BCS samples than those recovered by other primer sets (S10 Table; Fig 6), whereas primer set 3 largely failed to recover this taxon. Only a single Campylobacterota bathymodiolin symbiont (AB259697; S6 Table) was included in an alignment of potential symbionts from which the novel primers in this study were generated. A review of this alignment revealed six mismatches in the ps3 forward primer and a single mismatch in the reverse primer for that accession. Thus, the ps3 primers probably failed to appreciably capture Campylobacterota due to poor primer design. Differences in the phylogenetic signal of each amplicon might also contribute to variability in taxonomic assignments.

## Discussion

### Presence of three *Bathymodiolus* species at MAS sites

Based on molecular evidence from two mitochondrial genes, most mussels sampled at the MAS were *Bathymodiolus childressi*. This finding expands both the geographic range of the species (known previously from the GOM and off the coast of Trinidad and Tobago [19]), and its upper margin of depth, to 362 m (BCS). Molecular data also revealed single individual likely conspecific with *B*. *mauritanicus*/*B*. *sp B* BAP at BCS and a single *B*. *heckerae* at NCS among our MAS samples. This is the first report of *B*. *mauritanicus/sp B* BAP above 1000m and north of the Caribbean in the northwestern Atlantic Ocean. Finding *B*. *heckerae* at 1494m at NCS expands its previous known depth range (2155m–3300m; [6, 9]). Though sympatry of bathymodiolins is common at GOM seep sites [24], this is the first reported co-occurrence of *B*. *childressi* with its sister species *B*. *mauritanicus/sp*. *B* BAP, or with *B*. *heckerae*. Considering the rarity of the latter two species in our sampling, co-occurrence may generally be more common than currently known, but easily overlooked when frequencies are skewed. Additional examples of sympatry among these species may be discovered with more intensive sampling at Atlantic seep sites. Local dominance of *B*. *childressi* has been reported elsewhere, even when other species occur in the vicinity. For example, a recent study reported extensive assemblages of *B*. *childressi* at four sites off the coast of Trinidad and Tobago [19], though *B*. *sp B* BAP has been reported at nearby seeps [22]. Competitive exclusion [74] and ecological limits (see refs 8–10 in [24]) remain potential ecological drivers of resource monopolization, and co-occurrence may be a transient rather than stable state.

The single individual of *B*. *heckerae* sampled at NCS may be a recruit from BRD (closest known occurrence) or from an undiscovered site closer to Norfolk Canyon. The bottom-water temperature at BRD, where *B*. *heckerae* was reported as the dominant seep community species, was 3.2°C [9], which is comparable to the temperature at NCS (3.9–4.1°C) and several degrees cooler than BCS (6.1–9.4°C) [13], perhaps making it intolerable for *B*. *heckerae* settlement and/or survival. On the other hand, *B*. *childressi*, whose previously documented depth ranged from 525m – 2284m, may tolerate a wider range of temperatures, explaining its abundance at BCS. The NCS site lies within the depth range of a turnover zone of seep fauna, identified between 1300-1700m in the GOM, where the dominant members of seep communities above 1300m are different from those found below 1700m [6]. Further exploration of deeper seep communities on the Atlantic margin is necessary to determine whether the pattern of species turnover at depth holds for seep communities outside the GOM.

Mitochondrial haplotype networks of the MAS mussels showed high genetic diversity and little geographic structuring of haplotypes including between MAS and GOM, similar to observations of *B*. *childressi* populations in the GOM throughout their depth and geographic range [24, 75]. The lack of genetic structuring over thousands of kilometers may reflect high dispersal ability. *B*. *childressi* larvae from the GOM have been projected to reach the Mid-Atlantic based upon ocean circulation and Lagrangian larval transport models [26]. Additionally, *B*. *childressi* larvae have been recovered in plankton tows and their larvae can survive up to a year in the water column [25]. Genetic connectivity across disjunct chemosynthetic ecosystems of the deep Atlantic Equatorial Belt has been demonstrated in other seep species as well, such as alvinocarid shrimp and vesicomyid clams from vents on the MAR (vent sites Lucky Strike to Clueless), and seeps from WAF, BAP, Mid-Cayman Ridge, BRD, and the GOM [76].

### Evidence of thiotrophy within MAS *Bathymodiolus childressi*

Since the discovery and characterization of the endosymbiont within the gills of *Bathymodiolus childressi*, the assumption has been that this species derives its nutrition solely from methanotrophy via a single Gammaproteobacteria methanotrophic phylotype [34, 73, 77] even though other sympatric and neighboring species of bathymodiolins harbor both methanotrophs and thiotrophs (i.e. *B*. *brooksi*, and *B*. *heckerae*). In accordance with these previous studies, we found a dominant Gammaproteobacteria methanotroph present in all ten MAS mussels (nine *B*. *childressi*, one *B cf*. *mauritanicus*) we analyzed. However, we also found two phylotypes of Campylobacterota present in four of the ten mussels, with both phylotypes co-occurring within mussels. Phylotype C1 belonged to the same phylogenetic clade as the Campylobacterota recovered from GOM *B*. *childressi* [44], but Phylotype C2 belonged to a clade shared by sulfur-oxidizing Campylobacterota (identified as Epsilonproteobacteria) recovered from a deep-sea, wood-feeding gastropod [78]. The fact that the MAS mussel Campylobacterota are closely related to other known sulfur-oxidizers from marine habitats lends compelling but indirect evidence (i.e. estimation of ecological roles based on phylogenetic relationships, [79]) that MAS and GOM *B*. *childressi* might be benefiting from thiotrophy to some degree via Campylobacterota epibionts living in dual symbiosis [44].

Given the commonality of specimens, results from this study can be directly compared with those presented in [13] whereby gill stable isotope values were used to evaluate the relative importance of methane and sulfur as energy sources. Based on their sulfur isotope (δ^34^S) results suggesting utilization of H_2_S as a potential energy source, we expected to see thiotrophs in larger abundance in BCS than in NCS. Instead, the mussels analyzed from NCS had abundances of Campylobacterota roughly equal to the Gammaproteobacteria methanotroph in their gills whereas most BCS mussels had only trace amounts of Campylobacterota, with one exception. Furthermore, the highest δ^34^S values from [13] came from mussels containing Campylobacterota. However, higher δ^34^S values do not preclude the presence of thiotrophs, as observed in *Bathymodiolus mauritanicus* from the GOC [41, 71]. In general, isotopic values for mytilids tend to be variable and dependent upon many factors such as nutrition, tissue turnover time, type of symbiont and relative abundance, ontogeny, and local environmental conditions [41]. For example, the almost complete lack of Campylobacterota in BCS mussels despite the isotopic evidence of a sulfide source could indicate bacterial turnover within the gill. *Bathymodiolus childressi* ingests its methanotrophic Gammaproteobacteria endosymbiont, contained in bacteriocytes within the gill, to acquire nutrition [17, 80]. If mussels digest symbionts and recapture new ones throughout their lifetime, or utilize resources from transient epibionts, then tissue isotope values may represent a time-integrated diet, which reflects assimilated sulfur-derived nutrients only when thiotrophs are present. Furthermore, Campylobacterota epibionts that are closely related to, if not synonymous with, our Campylobacterota Phylotype C2 (Fig 5), switch from autotrophy to mixotrophy and/or heterotrophy throughout their life cycle in their host, *Rimicaris exoculata* [81]. Given both the gill and periostracum of MAS mussels had variable δ^34^S values [13], mixotrophy including thiotrophy may be characteristic of *B*. *childressi* at MAS sites.

### Plasticity of epibionts

The fact that we recovered Campylobacterota from one of four sampling events from BCS and the single dive from NCS suggests a patchy distribution of the epibionts on a relatively small geographic scale. In contrast, all *Bathymodiolus childressi* from five GOM sampling locations contained Campylobacterota and all three *B*. *sp B* BAP (referred to as *B*. *mauritanicus* in the study) contained Campylobacterota [44]. We did not observe Campylobacterota nor a Gammaproteobacteria thiotroph in our single *B*. *cf*. *mauritanicus* sample from BCS.

Symbiont abundance plays a key role in adaptation to fluctuating environmental conditions [82]. Absolute and relative abundances of Gammaproteobacteria endosymbiotic methanotrophs and thiotrophs in bathymodiolin hosts have been shown to vary between sampling sites and within conspecific hosts from different locations [41]. In some species known to harbor both methanotrophs and thiotrophs, the symbiont phylogenetic patterns suggest that methanotrophic endosymbionts may be host-specific and thusly coevolving with their hosts whereas thiotrophic symbionts can be found in a wider range of hosts [83]. In aquaria, pulses of sulfur led to changes in abundance of sulfur oxidizers and densities of symbionts varied over time [84, 85], proving that this differential is due to direct, real-time responses of sulfur-oxidizers to changing environmental conditions. In fact, the variation in symbiont communities within host individuals may be a mechanism of adaptation to different microhabitats [72] or substrates [83] or a response to stress or nutritional shifts in the host, as seen in corals and insects [86, 87]. Plasticity extends to life history as well, with some Gammaproteobacteria thiotrophs, closely related to bathymodiolin endosymbionts, found to be extracellular [88, 89] and/or heterotrophic [88]. Regarding symbiont evolution, epibiotic life stages of microbes may be an intermediate between free-living and complete dependency [29, 90]. Perhaps the Campylobacterota found in the MAS mussels and others found globally are in the intermediate stages leading to a symbiotic lifestyle. Uncovering how symbionts are acquired, selected, or replaced during evolution may address questions of specificity and host/symbiont co-speciation over short time spans [41]. Furthermore, future comparisons between Campylobacterota found on hosts versus those isolated from surrounding seawater may provide insight into the life history adaptability of these microbes. Free-living Campylobacterota have been recognized as an ecologically significant group of bacteria in deep-sea hydrothermal environments [91] and cold seeps [92]. These recent findings that suggest close coupling between Campylobacterota and host fauna from chemosynthetic environments further demonstrate the ecological significance of these microbes.

Differences in benthic macrofauna abundances between the two sites were observed as well. Video surveys of NCS and BCS macrofaunal communities showed that only two macroinvertebrate species, one being *Bathymodiolus childressi*, were shared between sites and the distribution and cover of live mussels, considered to be a biological indicator of seepage activity, was patchy at BCS [13]. Similarly, NCS mussel beds were different from other habitats within NCS and all BCS habitats regarding macro-infaunal abundances [14]. In this case, habitat differences in quality and source of organic matter were posited as the drivers of the infaunal assemblage differences. These observed differences in species assemblages between BCS and NCS at macrofaunal and microbial trophic levels are intriguing. However, whether the mechanisms linking the differences among the trophic levels are temporally stable and/or more broadly geographically applicable remains to be seen.

### Use of metabarcoding to evaluate symbiont diversity

Microbial community profiling promises to better reveal the bacterial types present in host gills and may provide semi-quantitative estimates of their relative abundance. This approach could help researchers understand the permissiveness of hosts to different symbionts, intra-host dynamics, and the impact of nutrient availability on these interactions. Here we showed that short 16S amplicons can differentiate major clades of symbionts and were broadly consistent in the relative amounts of Campylobacterota identified in each mussel sample (excluding primer set 3, which was shown to be poorly designed for amplification of Campylobacterota, Fig 6). All primer pairs consistently amplified the predominant methanotroph clade within Gammaproteobacteria, and our workflow consistently identified this taxon as Methylomonaceae regardless of the fragment of the 16S gene that was examined. While this consistency across amplicons alleviated some concern about how amplification biases impact quantitative interpretations, sequencing of control mixtures should be used in future work to better detect potential biases.

Amplicon libraries are known to have higher rates of error than shotgun libraries on Illumina platforms, particularly the second read of read pairs [66]. Reads mapped to the Phylotype M reconstructed consensus sequence showed high mismatch rates at their 3’ ends (S4 Fig; S12 Table), sometimes exceeding 10%, as is typical of Illumina sequencing [93–95]. This high realized error rate suggests a higher PhiX control spike would be beneficial. Second, a curated reference database of known symbionts would likely achieve better resolution than a highly inclusive database like SILVA, and both databases could be used sequentially to limit false positives. The presence of putative chimeric reads was suggested by patterns of reads that failed to map to symbiont phylotypes: the number of reads in each sample that failed to map stringently was correlated with the haplotype diversity present in the samples (S5 Fig; S11 Table). This result suggested that samples with multiple symbionts at moderate frequency generated significant numbers of chimeras during PCR.

The longer 16S sequence models we hypothesized by consensus generation and verified with Sanger sequencing were essential for understanding the phylogenetic placement of MAS symbionts. Our ability to extract these 16S sequences hinged on the significant body of 16S sequences available and the expected low complexity of the symbiont community. For low-complexity communities, direct assembly of amplicons by overlapping may be feasible, as in this study, resulting in candidate 16S phylotypes than can be verified by traditional means. On the other hand, long 16S sequences were not needed to detect interpretable sample- and site-level variation among MAS symbionts.

## Conclusion

This study characterizes the foundational species from the first seep communities discovered on the U.S. Atlantic seaboard north of BRD. Three bathymodiolin species were present at the three seeps, with *Bathymodiolus childressi* being by far the most abundant. The presence of single individuals of other species (*B*. *mauritanicus* at BCS and *B*. *heckerae* at NCS) raises interesting questions regarding dispersal and drivers of distribution within the bathymodiolins. This study coupled with results from [13] provide indirect but compelling evidence that *B*. *childressi* utilizes sulfur for metabolism through thiotrophic Campylobacterota epibionts, though there is a contrasting pattern between abundance of the Campylobacterota and isotope signatures between sites. These results draw a complex picture of associations between mussels and symbiotic bacteria in the Northeast Atlantic, which may vary depending on local characteristics of the habitats and microbial interactions. Perhaps the mussels’ ability to take advantage of thiotrophic bacteria is transient, considering the observed variation in the periostracum δ^13^C as well as small changes in the δ^34^S [13], which is the best analog for changes over the mussels’ lifespan. Whether the variability observed in the thiotrophy signal between gill samples at this site is due to micro-spatial differential chemical signatures between sampled mussels, or temporal variability of the chemical signatures, remains to be seen. Future studies that couple environmental measurements of chemical species, samples of ambient water, and gill microbiomes in tandem will further elucidate the role that the Campylobacterota epibionts play in host nutrition as well as in oceanic carbon and sulfur cycling.

## Supporting information

S1 Fig Neighbor-joining 16S phylogenies with the consensus sequences of the 50 most abundant Campylobacterota OTUs. Abundance was based on read count (red and blue clades) from a single mussel sample, MASM22, and a broad set of reference sequences representing known bathymodiolin symbionts. Consensus sequences clustered around reference sequences KU573880 (red dot) or FM994669 (blue dot) in both A) primer set 1 B) primer set 2 trees. S7 Table contains the BLAST results used to construct the consensus sequences.
(TIF)

S2 Fig Percent reads recovered from the MiSeq run that passed filter (PF) per primer set (ps1-4) per sample. Dark blue sample names are from Norfolk Canyon Seep. Light blue sample names are from Baltimore Canyon Seep. Exp. Avg. = expected average.
(TIF)

S3 Fig Read counts by phylotype. See S11 Table for values used to make the figure.
(TIF)

S4 Fig Percent coverage and relative mismatch of reads from four primer sets. Reads mapped via Bowtie2 to a full length 16S reference sequence. The x-axis represents the length of the sequence in nucleotides. The relative coverage of mapped reads across the reference sequences is represented by the black line. Mismatch frequency between the mapped reads and the reference sequence is shown by the red line. See S12 Table for values used to make the figure.
(TIF)

S5 Fig Relationship between evenness of counts among reads that map to the three dominant 16S phylotypes, and the percent of unmapped reads. See S12 Table for values used to make the figure.
(TIF)

S1 Table Comprehensive sampling information of bathymodiolins from Mid-Atlantic seep sites. BCS = Baltimore Canyon Seep, NCS = Norfolk Canyon Seep, CTS = Chincoteague Seep. “x” indicates whether the mussel gill microbiome was sequenced at 16S and whether its COI+ND4 haplotype was used in the phylogeny (phy).
(DOCX)

S2 Table **A) PCR recipes and B) thermal cycler conditions for the two loci amplified in mussels and three bacterial phylotypes**. ^1^ Recipe used for amplification of COI in HRS samples. * GoTaq Flexi (Promega), **(Promega), ^§^GeneAmp (Thermofisher), ^†^New England Biolabs. See main text for primer references. ^2^Conditions used for amplification with BathCOIF/R primers.
(DOCX)

S3 Table Genbank sequences from *Bathymodiolus spp*. included in the Bayesian phylogeny. (DOCX)

S4 Table Genbank sequences from *Bathymodiolus childressi* (*B*. *chi*) and *B*. *mauritanicus* (*B*. *mau*) included in the COI or ND4 network and AMOVA datasets. COI = Cytochrome Oxidase I, ND4 = NADH dehydrogenase subunit 4. *B*. *chi* = *Bathymodiolus childressi*, *B*. *mau* = *Bathymodiolus mauritanicus*. Individuals whose “Site” is specified were used in the AMOVA analysis (see Table 3). Sites are from northern Gulf of Mexico sample sites and coded as in (23). *N* = the number of sequences per species per site; ^1^ Reported as *Bathymodiolus sp*. B Nigerian seep "short" ^2^ Reported as *Bathymodiolus sp*. B (BathDS) ^3^ Site locations inferred from latitude/longitude coordinates reported. GOM = Gulf of Mexico BAP = Barbados Accretionary Prism, GOC = Gulf of Cadiz, WAF = western Africa.
(DOCX)

S5 Table Primers used for metabarcoding. V1-V8 are the variable regions within 16S (104) that are amplified by the primer sets. The final column contains the relative nucleotide (nt) position to *E*.*coli* 16S. * ps1, the universal Illumina primers, correspond to Bakt341F and Bakt805R (105). **The forward primer of primer set ps2 is a modified version of Bact27bF, (106) and the reverse primer is a modified version of 534R (107).
(DOCX)

S6 Table Genbank accession numbers identifying 16S sequences from symbionts of *Bathymodiolus spp*. meth = methanotroph thio = thiotroph. (DOCX)

S7 Table BLASTn (discontinuous Megablast) results for 50 randomly selected contigs for ps1 and ps2 from sample MASM22. Contig = merged read pair from MiSeq amplicon data. ps1 = primer set 1, ps2 = primer set 2, Search database was NCBI nucleotide (nt) database, 7/11/17 download date. eval, bit, id%, Top Hit are outputs from Blast searches explained on NCBI’s website: https://www.ncbi.nlm.nih.gov/BLAST/tutorial/Altschul-1.html. Note that JQ844779 is 99% identical with 100% query coverage to AM236329.
(DOCX)

S8 Table Sequences included in the Methylomonaceae (Fig 4) and the Campylobacterota (Fig 5) phylogenies. (DOCX)

S9 Table Miseq read counts. Reported per primer set (ps1-4) per mussel, after processing by the Mothur pipeline, and after singletons were trimmed in R’s Phyloseq package.
(DOCX)

S10 Table Number of reads attributed to unique families per sample for all four primer sets. This dataset was created in the R package Phyloseq. NCS = Norfolk Canyon Site; BCS = Baltimore Canyon Site. If taxonomies could not be resolved to higher levels, then they were left blank. Only those families with a total of 10 or more reads were listed.
(DOCX)

S11 Table Values used to calculate reads per phylotype (S3 Fig) and correlations between Simpson’s diversity and % unmapped reads (S5 Fig). NCS samples are shown in bold. Number of reads are shown per sample per primer set (ps1-4). recon Phy = reconstructed phylotype. C1, C2, and M are the reconstructed phylotypes where the reference sequence KU573880.1, FM994669.1, or AM236329 was used as a scaffold, respectively. See text for details. C1% = # reads from recon Phy C1/ # reads from recon Phy C1 + # reads from recon Phy C2 + # reads from recon Phy M; C2% = # reads from recon Phy C2/ # reads from recon Phy C1 + # reads from recon Phy C2 + # reads from recon Phy M; M% = # reads from recon Phy M/ # reads from recon Phy C1 + # reads from recon Phy C2 + # reads from recon Phy M; Simpson’s Diversity Index (D) = 1- (C1%^2)—(C2%^2)-(M%^2).
(DOCX)

S12 Table Values used to calculate mismatch percentage and read coverage for S4 Fig. Nucleotide positions range from 1 to 1184, referring to the length of the 16S consensus sequence used as a reference. Mismatch percentage indicates how many nucleotide differences occurred at that nucleotide position. Coverage = the number of nucleotides from our reads that mapped to that nucleotide position.
(DOCX)
