# Supplementary figures and images for: Molecular characterization of Bathymodiolus mussels and gill symbionts associated with chemosynthetic habitats from the U.S. Atlantic margin
Source: PLoS One. 2019 Mar 14;14(3):e0211616. doi: 10.1371/journal.pone.0211616 (PMC6417655; doi:10.1371/journal.pone.0211616)

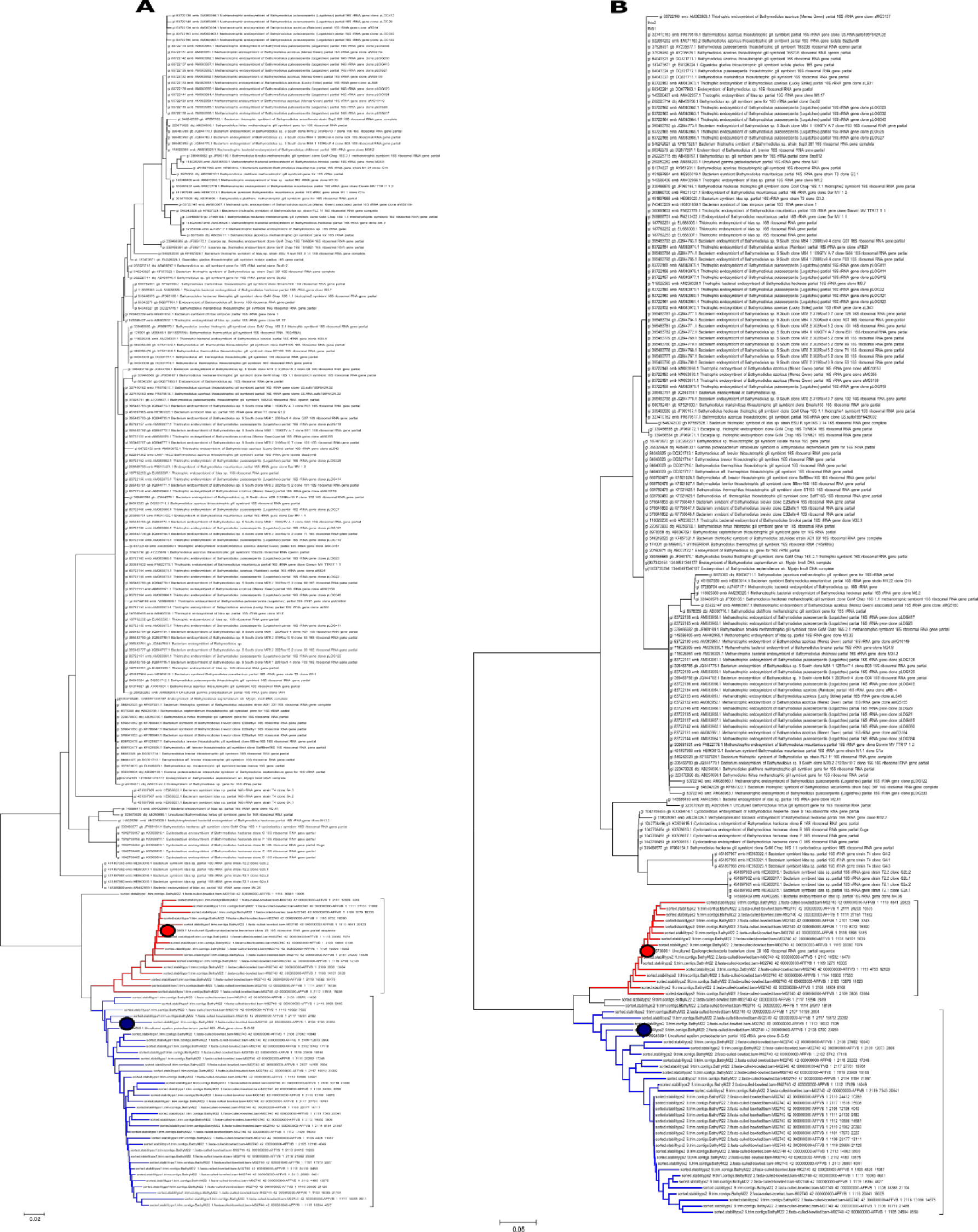

Supplement: S1 Fig — Abundance was based on read count (red and blue clades) from a single mussel sample, MASM22, and a broad set of reference sequences representing known bathymodiolin symbionts. Consensus sequences clustered around reference sequences KU573880 (red dot) or FM994669 (blue dot) in both A) primer set 1 B) primer set 2 trees. S7 Table contains the BLAST results used to construct the consensus sequences. (TIF) [file pone.0211616.s001.tif]

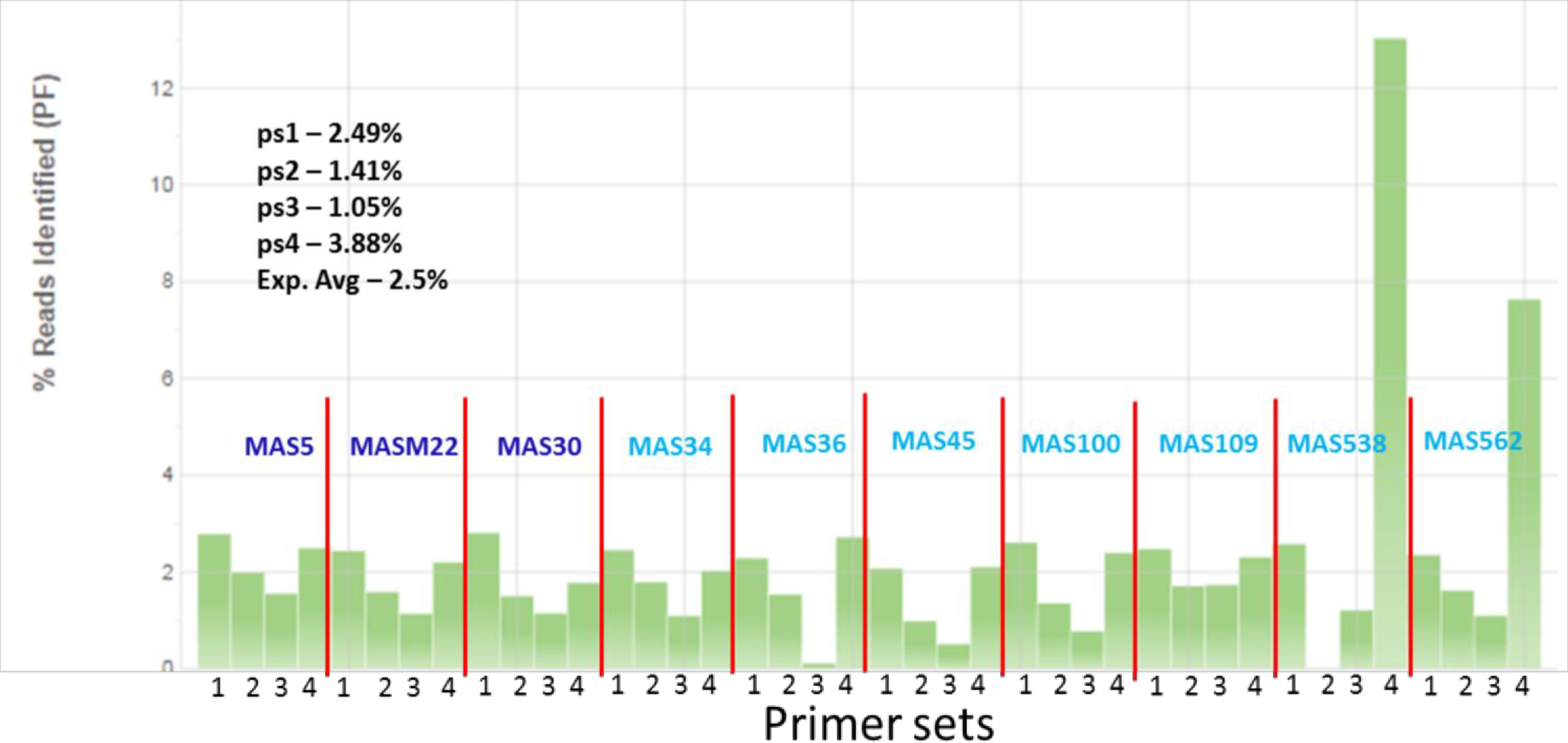

Supplement: S2 Fig — Dark blue sample names are from Norfolk Canyon Seep. Light blue sample names are from Baltimore Canyon Seep. Exp. Avg. = expected average. (TIF) [file pone.0211616.s002.tif]

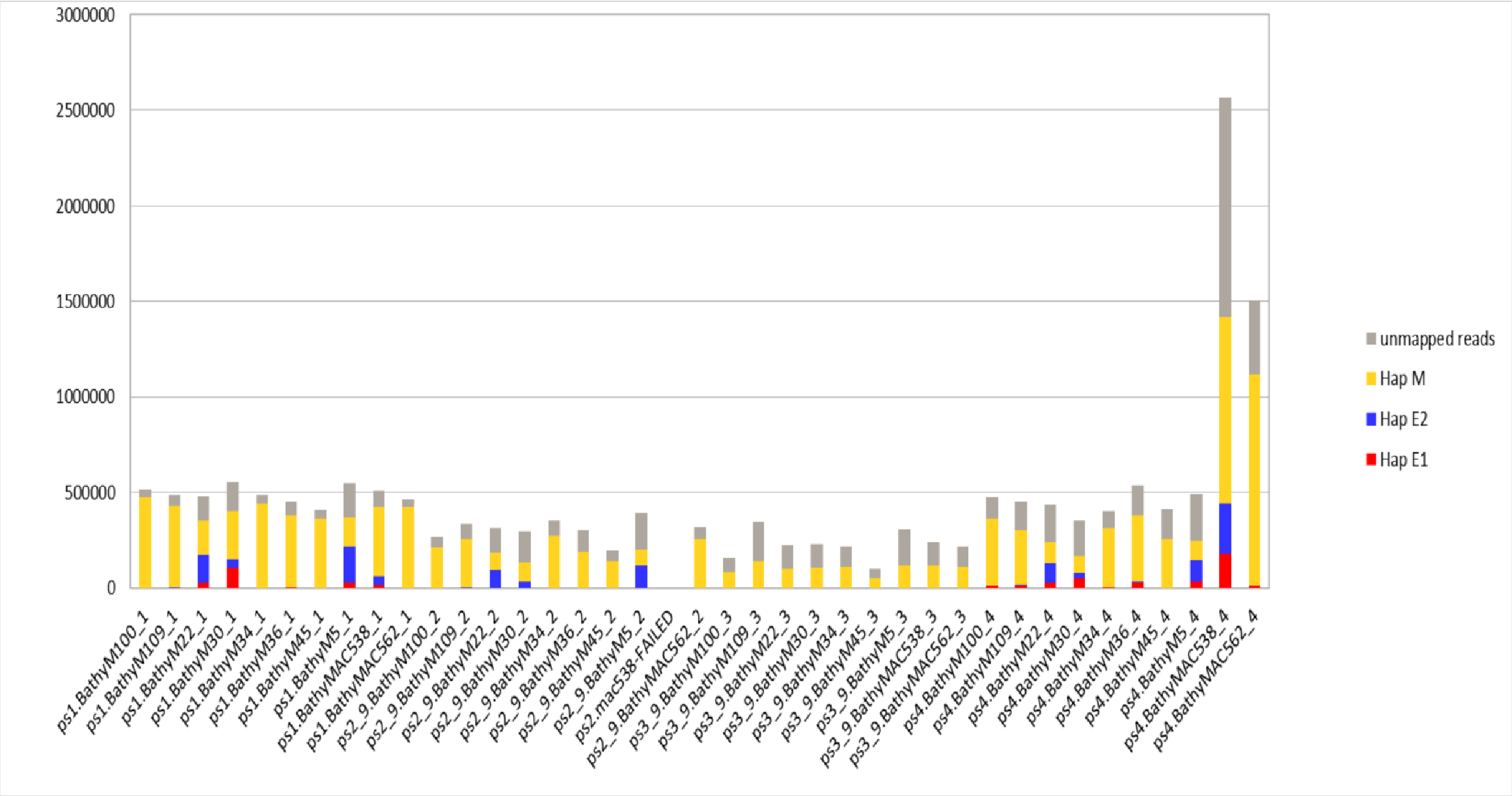

Supplement: S3 Fig — See S11 Table for values used to make the figure. (TIF) [file pone.0211616.s003.tif]

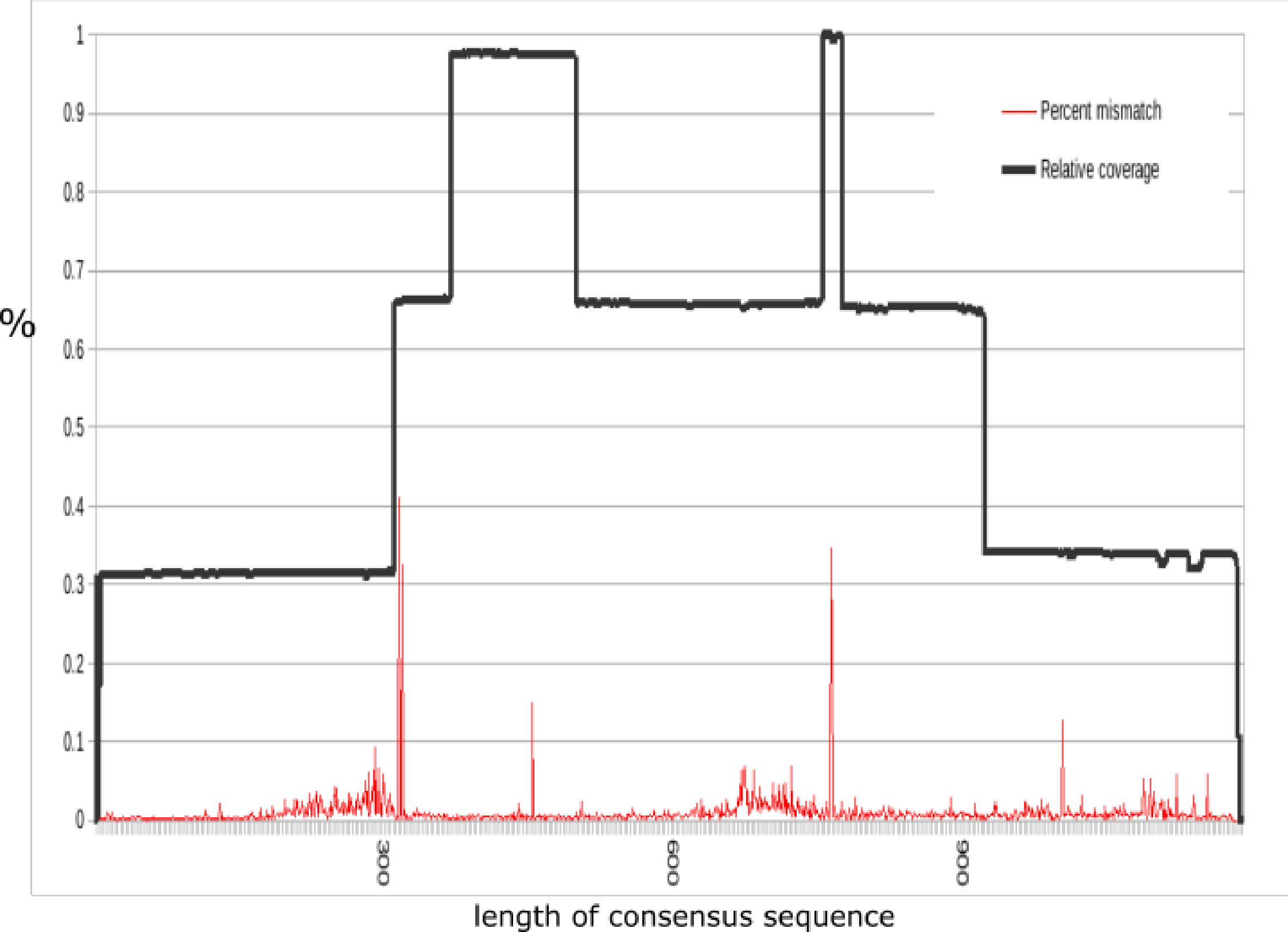

Supplement: S4 Fig — Reads mapped via Bowtie2 to a full length 16S reference sequence. The x-axis represents the length of the sequence in nucleotides. The relative coverage of mapped reads across the reference sequences is represented by the black line. Mismatch frequency between the mapped reads and the reference sequence is shown by the red line. See S12 Table for values used to make the figure. (TIF) [file pone.0211616.s004.tif]

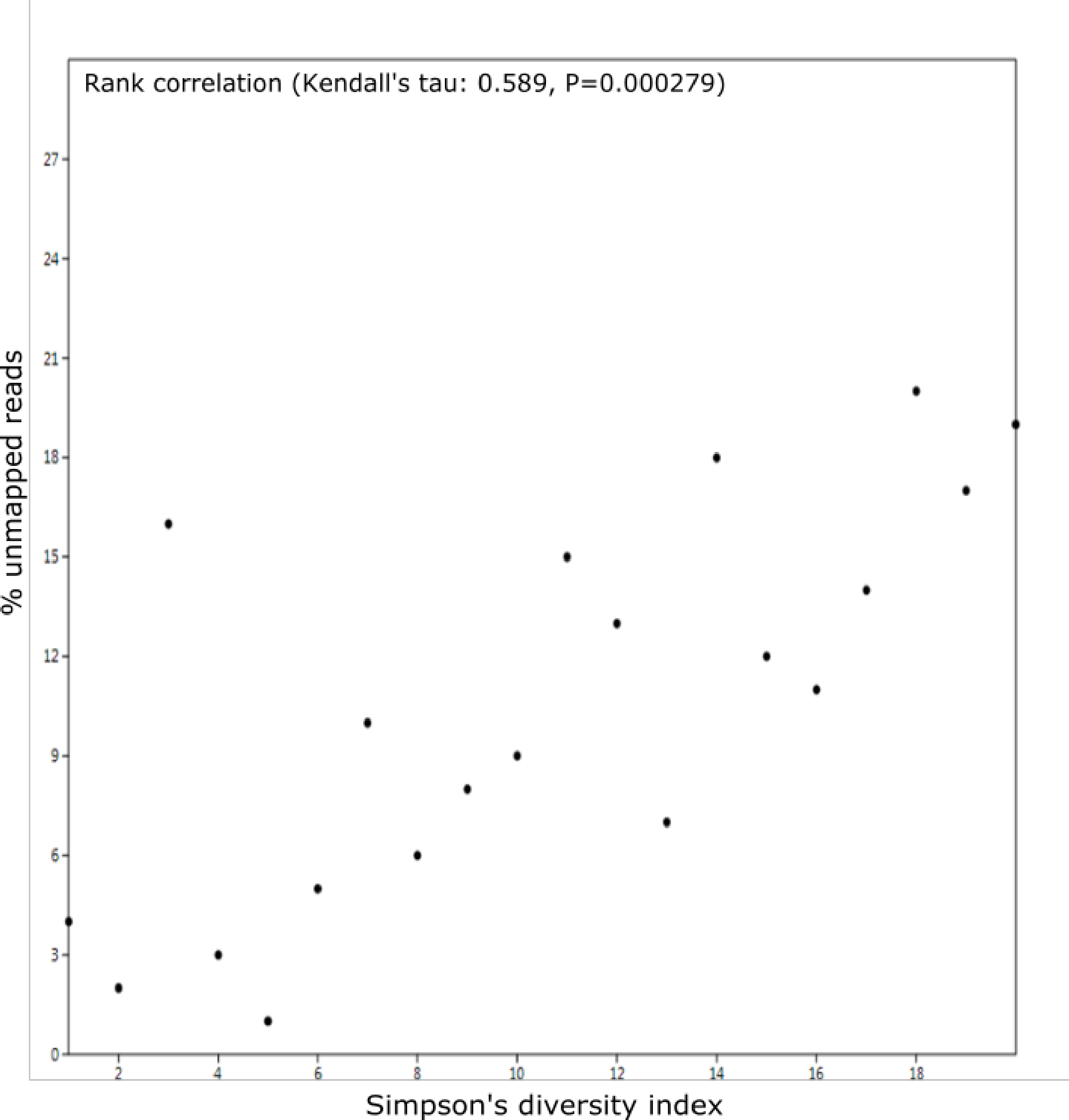

Supplement: S5 Fig — See S12 Table for values used to make the figure. (TIF) [file pone.0211616.s005.tif]
