# Supplementary material for: Molecular characterization of Bathymodiolus mussels and gill symbionts associated with chemosynthetic habitats from the U.S. Atlantic margin
Source: PLoS One. 2019 Mar 14;14(3):e0211616. doi: 10.1371/journal.pone.0211616 (PMC6417655; doi:10.1371/journal.pone.0211616)
Supplement: S1 Table — BCS = Baltimore Canyon Seep, NCS = Norfolk Canyon Seep, CTS = Chincoteague Seep. “x” indicates whether the mussel gill microbiome was sequenced at 16S and whether its COI+ND4 haplotype was used in the phylogeny (phy). (DOCX) [file pone.0211616.s006.docx]

Supplemental Table 1

| Sample # | Site | Dive/Station# | Date | COI | ND4 | 16S | Phy |
| --- | --- | --- | --- | --- | --- | --- | --- |
| HRS001 | NCS | GEX03-009 | 5/8/2017 | MG519967 |  |  |  |
| HRS004 | NCS | GEX03-009 | 5/8/2017 | MG519968 |  |  |  |
| HRS005 | NCS | GEX03-009 | 5/8/2017 | MG519969 |  |  |  |
| HRS007 | NCS | GEX03-009 | 5/8/2017 | MG519970 |  |  |  |
| HRS009 | NCS | GEX03-009 | 5/8/2017 | MG519971 |  |  |  |
| HRS011 | NCS | GEX03-009 | 5/8/2017 | MG519972 |  |  |  |
| HRS013 | NCS | GEX03-009 | 5/8/2017 | MG519973 |  |  |  |
| HRS015 | NCS | GEX03-011 | 5/8/2017 | MG519974 |  |  |  |
| HRS017 | NCS | GEX03-011 | 5/8/2017 | MG519975 |  |  |  |
| HRS019 | NCS | GEX03-011 | 5/8/2017 | MH723710 |  |  |  |
| HRS021 | NCS | GEX03-011 | 5/8/2017 |  | MG520015 |  |  |
| HRS023 | NCS | GEX03-011 | 5/8/2017 | MG519982 | MG520016 |  | x |
| HRS025 | NCS | GEX03-011 | 5/8/2017 |  | MG520017 |  |  |
| HRS028 | NCS | GEX03-011 | 5/8/2017 | MG519976 |  |  |  |
| HRS029 | NCS | GEX03-011 | 5/8/2017 | MG519977 |  |  |  |
| HRS031 | NCS | GEX03-011 | 5/8/2017 | MG519978 |  |  |  |
| HRS033 | NCS | GEX03-023 | 5/8/2017 | MG519979 |  |  |  |
| HRS035 | NCS | GEX03-023 | 5/8/2017 | MG519869 | MG520022 |  | x |
| HRS037 | NCS | GEX03-023 | 5/8/2017 | MG519980 |  |  |  |
| HRS039 | NCS | GEX03-023 | 5/8/2017 | MG519981 |  |  |  |
| HRS041 | NCS | GEX03-023 | 5/8/2017 | MH723711 |  |  |  |
| HRS043 | NCS | GEX03-023 | 5/8/2017 | MH723712 |  |  |  |
| HRS045 | NCS | GEX03-023 | 5/8/2017 | MH723713 |  |  |  |
| HRS047 | NCS | GEX03-023 | 5/8/2017 | MH723714 |  |  |  |
| HRS049 | NCS | GEX03-023 | 5/8/2017 | MH723715 |  |  |  |
| HRS051 | NCS | GEX03-023 | 5/8/2017 | MH723716 |  |  |  |
| HRS053 | NCS | GEX03-023 | 5/8/2017 | MH723717 |  |  |  |
| HRS055 | CTS | GEX04-032 | 5/9/2017 | MG519983 | MG520018 |  | x |
| HRS058 | CTS | GEX04-032 | 5/9/2017 | MH723718 |  |  |  |
| HRS059 | CTS | GEX04-032 | 5/9/2017 | MH723719 |  |  |  |
| HRS061 | CTS | GEX04-032 | 5/9/2017 | MH723720 |  |  |  |
| HRS063 | CTS | GEX04-032 | 5/9/2017 | MH723721 |  |  |  |
| HRS065 | CTS | GEX04-032 | 5/9/2017 | MH723722 |  |  |  |
| HRS067 | CTS | GEX04-032 | 5/9/2017 | MH723723 |  |  |  |
| HRS071 | CTS | GEX04-032 | 5/9/2017 | MH723724 |  |  |  |
| HRS073 | CTS | GEX04-032 | 5/9/2017 | MH723725 |  |  |  |
| HRS075 | CTS | GEX04-035 | 5/9/2017 | MH723726 |  |  |  |
| HRS077 | CTS | GEX04-035 | 5/9/2017 | MH723727 |  |  |  |
| HRS079 | CTS | GEX04-035 | 5/9/2017 | MH723728 |  |  |  |
| HRS081 | CTS | GEX04-035 | 5/9/2017 | MH723729 |  |  |  |
| HRS083 | CTS | GEX04-035 | 5/9/2017 | MH723730 |  |  |  |
| HRS085 | CTS | GEX04-035 | 5/9/2017 | MH723731 |  |  |  |
| HRS087 | CTS | GEX04-035 | 5/9/2017 | MH723732 |  |  |  |
| HRS089 | CTS | GEX04-035 | 5/9/2017 | MH723733 |  |  |  |
| HRS091 | CTS | GEX04-035 | 5/9/2017 | MH723734 |  |  |  |
| HRS093 | CTS | GEX04-035 | 5/9/2017 | MH723735 | MG520019 |  |  |
| HRS095 | CTS | GEX05-053 | 5/10/2017 | MH723736 | MG520020 |  |  |
| HRS097 | CTS | GEX05-053 | 5/10/2017 | MH723737 |  |  |  |
| HRS100 | CTS | GEX05-053 | 5/10/2017 | MH723738 |  |  |  |
| HRS101 | CTS | GEX05-053 | 5/10/2017 | MH723739 |  |  |  |
| HRS104 | CTS | GEX05-053 | 5/10/2017 | MH723740 |  |  |  |
| HRS106 | CTS | GEX05-053 | 5/10/2017 | MH723741 |  |  |  |
| HRS108 | CTS | GEX05-053 | 5/10/2017 | MH723742 |  |  |  |
| HRS110 | CTS | GEX05-053 | 5/10/2017 | MH723743 |  |  |  |
| HRS112 | CTS | GEX05-053 | 5/10/2017 | MH723744 |  |  |  |
| HRS114 | CTS | GEX05-053 | 5/10/2017 | MH723745 |  |  |  |
| HRS116 | CTS | GEX05-069 | 5/10/2017 | MH723746 |  |  |  |
| HRS118 | CTS | GEX05-069 | 5/10/2017 | MH723747 |  |  |  |
| HRS126 | CTS | GEX05-069 | 5/10/2017 | MH723748 |  |  |  |
| HRS128 | CTS | GEX05-069 | 5/10/2017 | MH723749 |  |  |  |
| HRS133 | CTS | GEX05-069 | 5/10/2017 | MH723750 |  |  |  |
| HRS134 | CTS | GEX05-069 | 5/10/2017 | MH723751 |  |  |  |
| HRS138 | BC | GEX05-075 | 5/11/2017 | MH723752 |  |  |  |
| HRS140 | BC | GEX05-075 | 5/11/2017 | MH723753 |  |  |  |
| HRS142 | BC | GEX05-075 | 5/11/2017 | MH723754 |  |  |  |
| HRS144 | BC | GEX05-075 | 5/11/2017 | MH723755 |  |  |  |
| MAS100 | BCS | B08 | 8/27/2012 |  |  | x |  |
| MAS101 | BCS | B08 | 8/27/2012 | MG519870 |  |  |  |
| MAS102 | BCS | B08 | 8/27/2012 | MG519871 |  |  |  |
| MAS103 | BCS | B08 | 8/27/2012 | MG519872 | MG520001 |  | x |
| MAS104 | BCS | B08 | 8/27/2012 | MG519873 |  |  |  |
| MAS105 | BCS | B08 | 8/27/2012 | MG519874 | MG520002 |  |  |
| MAS106 | BCS | B08 | 8/27/2012 | MG519875 |  |  |  |
| MAS107 | BCS | B08 | 8/27/2012 | MG519876 | MG520003 |  | x |
| MAS108 | BCS | B08 | 8/27/2012 | MG519877 |  |  |  |
| MAS109 | BCS | B08 | 8/27/2012 | MG519878 | MG520004 | x | x |
| MAS283 | NCS | N01 | 5/8/2013 | MG519879 |  |  |  |
| MAS284 | NCS | N01 | 5/8/2013 | MG519880 | MG520005 |  | x |
| MAS285 | NCS | N01 | 5/8/2013 | MG519881 | MG520006 |  | x |
| MAS286 | NCS | N01 | 5/8/2013 | MG519882 |  |  |  |
| MAS288 | NCS | N01 | 5/8/2013 | MG519883 |  |  |  |
| MAS289 | NCS | N01 | 5/8/2013 | MG519884 |  |  |  |
| MAS290 | NCS | N01 | 5/8/2013 | MG519885 |  |  |  |
| MAS291 | NCS | N01 | 5/8/2013 | MG519886 |  |  |  |
| MAS292 | NCS | N01 | 5/8/2013 | MG519887 |  |  |  |
| MAS293 | NCS | N01 | 5/8/2013 | MG519888 |  |  |  |
| MAS294 | NCS | N01 | 5/8/2013 | MG519889 |  |  |  |
| MAS295 | NCS | N01 | 5/8/2013 | MG519890 |  |  |  |
| MAS296 | NCS | N01 | 5/8/2013 | MG519891 |  |  |  |
| MAS297 | NCS | N01 | 5/8/2013 | MG519892 |  |  |  |
| MAS298 | NCS | N01 | 5/8/2013 | MG519893 |  |  |  |
| MAS299 | NCS | N01 | 5/8/2013 | MG519894 |  |  |  |
| MAS300 | BCS | B08 | 8/27/2012 | MG519895 |  |  |  |
| MAS301 | BCS | B08 | 8/27/2012 | MG519896 | MG520007 |  | x |
| MAS302 | BCS | B08 | 8/27/2012 | MG519897 | MG520008 |  |  |
| MAS303 | BCS | B08 | 8/27/2012 | MG519898 | MG519984 |  |  |
| MAS304 | BCS | B08 | 8/27/2012 | MG519899 |  |  |  |
| MAS306 | NCS | N01 | 5/8/2013 | MG519900 | MG519985 |  | x |
| MAS310 | NCS | N01 | 5/8/2013 | MG519901 |  |  |  |
| MAS311 | NCS | N01 | 5/8/2013 | MG519902 | MG519986 |  | x |
| MAS313 | NCS | N01 | 5/8/2013 | MG519903 |  |  |  |
| MAS314 | NCS | N01 | 5/8/2013 | MG519904 |  |  |  |
| MAS320 | NCS | N01 | 5/8/2013 | MG519905 |  |  |  |
| MAS321 | NCS | N01 | 5/8/2013 | MG519906 |  |  |  |
| MAS322 | NCS | N01 | 5/8/2013 | MG519907 |  |  |  |
| MAS323 | NCS | N01 | 5/8/2013 | MG519908 |  |  |  |
| MAS326 | NCS | N01 | 5/8/2013 | MG519909 |  |  |  |
| MAS327 | NCS | N01 | 5/8/2013 | MG519910 |  |  |  |
| MAS338 | NCS | J2683 | 5/9/2013 | MG519911 |  |  |  |
| MAS339 | NCS | J2683 | 5/9/2013 | MG519912 | MG519990 |  | x |
| MAS340 | NCS | J2683 | 5/9/2013 | MG519913 | MG519987 |  | x |
| MAS341 | NCS | J2683 | 5/9/2013 | MG519914 |  |  |  |
| MAS343 | NCS | J2683 | 5/9/2013 | MG519915 | MG520009 |  | x |
| MAS346 | NCS | J2683 | 5/9/2013 | MG519916 | MG520010 |  | x |
| MAS347 | NCS | J2683 | 5/9/2013 | MG519917 |  |  |  |
| MAS348 | NCS | J2683 | 5/9/2013 | MG519918 |  |  |  |
| MAS349 | NCS | J2683 | 5/9/2013 | MG519919 |  |  |  |
| MAS350 | NCS | J2683 | 5/9/2013 | MG519920 | MG519991 |  | x |
| MAS351 | NCS | J2683 | 5/9/2013 | MG519921 | MG520011 |  | x |
| MAS352 | NCS | J2683 | 5/9/2013 | MG519922 |  |  |  |
| MAS359 | NCS | J2683 | 5/9/2013 | MG519923 |  |  |  |
| MAS360 | NCS | J2683 | 5/9/2013 | MG519924 |  |  |  |
| MAS361 | NCS | J2683 | 5/9/2013 | MG519925 |  |  |  |
| MAS362 | NCS | J2683 | 5/9/2013 | MG519926 |  |  |  |
| MAS363 | NCS | J2683 | 5/9/2013 | MG519927 |  |  |  |
| MAS364 | NCS | J2683 | 5/9/2013 | MG519928 |  |  |  |
| MAS365 | NCS | J2683 | 5/9/2013 | MG519929 |  |  |  |
| MAS366 | NCS | J2683 | 5/9/2013 | MG519930 |  |  |  |
| MAS367 | NCS | J2683 | 5/9/2013 | MG519931 |  |  |  |
| MAS368 | NCS | J2683 | 5/9/2013 | MG519932 |  |  |  |
| MAS369 | NCS | J2683 | 5/9/2013 | MG519933 |  |  |  |
| MAS373 | NCS | J2683 | 5/9/2013 | MG519934 |  |  |  |
| MAS374 | NCS | J2683 | 5/9/2013 | MG519935 |  |  |  |
| MAS375 | NCS | J2683 | 5/9/2013 | MG519936 |  |  |  |
| MAS376 | NCS | J2683 | 5/9/2013 | MG519937 | MG520012 |  | x |
| MAS377 | NCS | J2683 | 5/9/2013 | MG519938 |  |  |  |
| MAS537/538 | BCS | B689 | 5/16/2013 | MG519939 | MG520013 | x |  |
| MAS539 | BCS | B689 | 5/16/2013 | MG519940 |  |  |  |
| MAS540 | BCS | B689 | 5/16/2013 | MG519941 |  |  |  |
| MAS541 | BCS | B689 | 5/16/2013 | MG519942 |  |  |  |
| MAS542 | BCS | B689 | 5/16/2013 | MG519943 |  |  |  |
| MAS543 | BCS | B689 | 5/16/2013 | MG519944 |  |  |  |
| MAS544 | BCS | B689 | 5/16/2013 | MG519945 |  |  |  |
| MAS545 | BCS | B689 | 5/16/2013 | MG519946 |  |  |  |
| MAS546 | BCS | B689 | 5/16/2013 | MG519947 |  |  |  |
| MAS547 | BCS | B689 | 5/16/2013 | MG519948 |  |  |  |
| MAS548 | BCS | B689 | 5/16/2013 | MG519949 |  |  |  |
| MAS549 | BCS | B689 | 5/16/2013 | MG519950 |  |  |  |
| MAS550 | BCS | B689 | 5/16/2013 | MG519951 |  |  |  |
| MAS551 | BCS | B689 | 5/16/2013 | MG519952 |  |  |  |
| MAS552 | BCS | B689 | 5/16/2013 | MG519953 |  |  |  |
| MAS553 | BCS | B689 | 5/16/2013 | MG519954 |  |  |  |
| MAS554 | BCS | B689 | 5/16/2013 | MG519955 | MG519988 |  | x |
| MAS555 | BCS | B689 | 5/16/2013 | MG519956 | MG519989 |  | x |
| MAS557 | BCS | B689 | 5/16/2013 | MG519957 |  |  |  |
| MAS561/562 | BCS | B689 | 5/16/2013 | MG519958 | MG520014 | x |  |
| MASm17 | NCS | N01 | 5/8/2013 |  | MG519996 |  |  |
| MASm18 | NCS | N01 | 5/8/2013 |  | MG519997 |  |  |
| MASm22 | NCS | N01 | 5/8/2013 |  |  | x |  |
| MASm24 | NCS | N01 | 5/8/2013 |  | MG519998 |  |  |
| MASm3 | NCS | N01 | 5/8/2013 |  | MG519992 |  |  |
| MASm30 | NCS | N01 | 5/8/2013 |  |  | x |  |
| MASm32 | BCS | B14 | 9/7/2012 | MG519959 | MG519999 |  |  |
| MASm33 | BCS | B14 | 9/7/2012 | MG519960 | MG520000 |  |  |
| MASm34 | BCS | B14 | 9/7/2012 | MG519868 | MG520021 | x | x |
| MASm35 | BCS | B14 | 9/7/2012 | MG519961 |  |  |  |
| MASm36 | BCS | B14 | 9/7/2012 | MG519962 |  | x |  |
| MASm39 | BCS | B14 | 9/7/2012 | MG519963 |  |  |  |
| MASm44 | BCS | B14 | 9/7/2012 | MG519964 |  |  |  |
| MASm45 | BCS | B14 | 9/7/2012 | MG519965 |  | x |  |
| MASm46 | BCS | B14 | 9/7/2012 | MG519966 |  |  |  |
| MASm5 | NCS | N01 | 5/8/2013 |  |  | x |  |
| MASm7 | NCS | N01 | 5/8/2013 |  | MG519993 |  |  |
| MASm8 | NCS | N01 | 5/8/2013 |  | MG519994 |  |  |
| MASm9 | NCS | N01 | 5/8/2013 |  | MG519995 |  |  |
|  |  |  | TOTAL | 162 | 39 | 10 | 21 |
