# Supplementary material for: Molecular characterization of Bathymodiolus mussels and gill symbionts associated with chemosynthetic habitats from the U.S. Atlantic margin
Source: PLoS One. 2019 Mar 14;14(3):e0211616. doi: 10.1371/journal.pone.0211616 (PMC6417655; doi:10.1371/journal.pone.0211616)
Supplement: S2 Table — A) PCR recipes and B) thermal cycler conditions for the two loci amplified in mussels and three bacterial phylotypes. 1 Recipe used for amplification of COI in HRS samples. * GoTaq Flexi (Promega), **(Promega), §GeneAmp (Thermofisher), †New England Biolabs. See main text for primer references. 2Conditions used for amplification with BathCOIF/R primers. (DOCX) [file pone.0211616.s007.docx]

Supplemental Table 2A

|  | Locus | [ buffer]* | [MgCl_2_]*  mM | [dNTPs]^§^  mM | [BSA]^†^ | [P1]  µM | [P2]  µM | *Taq**  U/µl | [DNA]  ng | PCR vol  µl | Primer1 | Primer2 |  |
| --- | --- | --- | --- | --- | --- | --- | --- | --- | --- | --- | --- | --- | --- |
| mussel | COI | 1X | 2.5 | 2.0 | 4X | 0.5 | 0.5 | 4.0 | 2-200 | 50 | HCO2198 | LCO1490 | |
|  | COI^1^ | 1X | 2.0 | 0.12 | na | 0.2 | 0.2 | 0.5 | 2-200 | 25 | BathCOIF | BathCOIR | |
|  | ND4 | 1X | 2.5 | 2.0 | na | 0.4 | 0.4 | 1.25 | 2-200 | 25 | ArgBL | NAP2H | |
| symbiont | 16S | 0.5X | 2.0 | 2.0 | na | 0.5 | 0.5 | 0.1 | 13-600 | 25 | BathymethF | BathysymR | |
|  | 16S | 0.5X | 2.0 | 2.0 | na | 0.5 | 0.5 | 0.1 | 13-600 | 25 | BathyEpsilonKUF | BathysymR | |
|  | 16S | 0.5X | 2.0 | 2.0 | na | 0.5 | 0.5 | 0.1 | 13-600 | 25 | BathyEpsilonFMF | BathysymR | |

Supplemental Table 2B

| locus | initial denaturation | denaturation | annealing | extension | # cycles | denaturation | annealing | extension | final ext | # cycles |
| --- | --- | --- | --- | --- | --- | --- | --- | --- | --- | --- |
| COI | 94ºC; 3m | 94ºC; 60s | 50ºC; 60s | 72ºC; 60s | 35 |  |  |  | 72ºC; 7m |  |
| COI^2^ | 94ºC; 2m | 94ºC; 35s | 48ºC; 35s | 72ºC; 70s | 5 | 94ºC; 35s | 52ºC; 35s | 72ºC; 70s | 72ºC; 10m | 35 |
| ND4 | 96ºC; 10m | 96ºC; 30s | 50ºC; 30s | 72ºC; 30s | 35 |  |  |  | 72ºC; 7m |  |
| 16S | 95ºC;3m | 95ºC;30s | 55ºC; 30s | 72ºC; 30s | 35 |  |  |  | 72ºC; 5m |  |
