# Supplementary material for: Molecular characterization of Bathymodiolus mussels and gill symbionts associated with chemosynthetic habitats from the U.S. Atlantic margin
Source: PLoS One. 2019 Mar 14;14(3):e0211616. doi: 10.1371/journal.pone.0211616 (PMC6417655; doi:10.1371/journal.pone.0211616)
Supplement: S3 Table — (DOCX) [file pone.0211616.s008.docx]

Supplemental Table 3

| Species | code | ND4 | COI | Reference |
| --- | --- | --- | --- | --- |
| *Benthomodiolus lignicola* | Ben.lig | AY649817 | AY275545 | [1] |
| *Idas washingtonia* | I.was | AY649815 | AY275546 | [1] |
| *Tamu fisheri* | T.fis | AY649814 | AY649803 | [1] |
| *A. crypta* | A.cry | HF545182 | HF545105 | [2] |
| *B. aff thermophilus* | B.a.the | AY649809 | AF456317 | [1] |
| *B. aduloides* | B.adu | HF545200 | HF545118 | [2] |
| *B. azoricus* | B.azo | AF128534 | AY649795 | [1] |
| *B. brevior* | B.breLau | AY046277 | AY275544 | [1] |
| *B. brevior* | B.breMT | AY649806 | AY649799 | [1] |
| *B. brevior* | B.bre | HF545187 | HF545111 | [2] |
| *B. brooksi* | B.broGOM | AY130247 | AY649797 | [1] |
| *B. brooksi* | B.broWFE | AY649805 | AY649798 | [1] |
| *B. brooksi* | B.bro | HF545178 | HF545110 | [2] |
| *B. childressi* | B.chiAC | AY130248 | AY649800 | [1] |
| *B. heckerae* | B.hecBR | AY130245 | AY649793 | [1] |
| *B. heckerae* | B.hecWFE | AY130246 | AY649794 | [1] |
| *B. japonicus* | B.jap | HF545185 | HF545108 | [2] |
| *B. manuensis* | B.man | HF545184 | HF545107 | [2] |
| *B. marisindicus* | B.mar | AY046279 | AY275543 | [1] |
| *B. mauritanicus* | B.mau | AY649810 | AY649801 | [1] |
| *B. platifrons* | B.pla | HF545183 | HF545106 | [2] |
| *B. puteoserpentis* | B.put1 | AF128533 | AY649796 | [1] |
| *B. puteoserpentis* | B.put2 | HF545176 | HF545102 | [2] |
| *B. securiformis* | B.sec | HF545186 | HF545109 | [2] |
| *B. sp.* | B.sp.Kik | HF545188 | HF545112 | [2] |
| *B. sp.* | B.sp.Siss2 | HF545204 | HF545122 | [2] |
| *B. sp.* | B.sp.Siss1 | HF545217 | HF545125 | [2] |
| *B. tangaroa* | B.tan1 | AY649811 | AY608439 | [1] |
| *B. tangaroa* | B.tan2 | HF545203 | HF545121 | [2] |
| *B. thermophilus* | B.theA | AY649807 | AF456285 | [1] |
| *B. thermophilus* | B.theB | AY649808 | AF456303 | [1] |
| *G. gladius* | G.gla | AY649813 | AY649802 | [1] |
| *G. horikoshii* | G.hor | HF545190 | HF545113 | [2] |
| *G. sp.* | G.sp.Ait | HF545201 | HF545119 | [2] |
| *G. sp.* | G.sp.Ash | HF545202 | HF545120 | [2] |
| *B. sp.* | MAS103 | MG519872 | MG520001 | this study |
| *B. sp.* | MAS107 | MG519876 | MG520003 | this study |
| *B. sp.* | MAS109 | MG519878 | MG520004 | this study |
| *B. sp.* | MAS284 | MG519880 | MG520005 | this study |
| *B. sp.* | MAS285 | MG519881 | MG520006 | this study |
| *B. sp.* | MAS301 | MG519896 | MG520007 | this study |
| *B. sp.* | MAS306 | MG519900 | MG519985 | this study |
| *B. sp.* | MAS311 | MG519902 | MG519986 | this study |
| *B. sp.* | MAS339 | MG519912 | MG519990 | this study |
| *B. sp.* | MAS340 | MG519913 | MG519987 | this study |
| *B. sp.* | MAS343 | MG519915 | MG520009 | this study |
| *B. sp.* | MAS346 | MG519916 | MG520010 | this study |
| *B. sp.* | MAS350 | MG519920 | MG519991 | this study |
| *B. sp.* | MAS351 | MG519921 | MG520011 | this study |
| *B. sp.* | MAS376 | MG519937 | MG520012 | this study |
| *B. sp.* | MAS554 | MG519955 | MG519988 | this study |
| *B. sp.* | MAS555 | MG519956 | MG519989 | this study |
| *B. sp.* | MAS34 | MG519868 | MG520021 | this study |
| *B. sp.* | HRS023 | MG519982 | MG520016 | this study |
| *B. sp.* | HRS035 | MG519869 | MG520022 | this study |
| *B. sp.* | HRS055 | MG519983 | MG520018 | this study |

1. Jones WJ, Won YJ, Maas PAY, Smith PJ, Lutz RA, Vrijenhoek RC. Evolution of habitat use by deep-sea mussels. Mar Biol. 2006;148(4):841-51. doi: 10.1007/s00227-005-0115-1. PubMed PMID: ISI:000235058600015.

2. Lorion J, Kiel S, Faure B, Kawato M, Ho SY, Marshall B, et al. Adaptive radiation of chemosymbiotic deep-sea mussels. Proc Biol Sci. 2013;280(1770):20131243. doi: 10.1098/rspb.2013.1243. PubMed PMID: 24048154; PubMed Central PMCID: PMCPMC3779325.
