# Supplementary material for: Molecular characterization of Bathymodiolus mussels and gill symbionts associated with chemosynthetic habitats from the U.S. Atlantic margin
Source: PLoS One. 2019 Mar 14;14(3):e0211616. doi: 10.1371/journal.pone.0211616 (PMC6417655; doi:10.1371/journal.pone.0211616)
Supplement: S4 Table — COI = Cytochrome Oxidase I, ND4 = NADH dehydrogenase subunit 4. B. chi = Bathymodiolus childressi, B. mau = Bathymodiolus mauritanicus. Individuals whose “Site” is specified were used in the AMOVA analysis (see Table 3). Sites are from northern Gulf of Mexico sample sites and coded as in (23). N = the number of sequences per species per site; 1 Reported as Bathymodiolus sp. B Nigerian seep "short" 2 Reported as Bathymodiolus sp. B (BathDS) 3 Site locations inferred from latitude/longitude coordinates reported. GOM = Gulf of Mexico BAP = Barbados Accretionary Prism, GOC = Gulf of Cadiz, WAF = western Africa. (DOCX) [file pone.0211616.s009.docx]

Supplemental Table 4

| Gene | species | Region | Site | N | GB accession number | Reference |
| --- | --- | --- | --- | --- | --- | --- |
| COI | *B. chi* | GOM | GC234 | 10 | KM024170-179 | [1] |
|  |  |  | MC929 | 15 | KM024180-194 | [1] |
|  |  |  | GC233 | 10 | KM024195-204 | [1] |
|  |  |  | GC204 | 15 | KM024205-219 | [1] |
|  |  |  | GB647 | 6 | KM024220-225 | [1] |
|  |  |  | GB697 | 4 | KM024226-229 | [1] |
|  |  |  | MC853 | 8 | KM024230-237 | [1] |
|  |  |  | MC640 | 3 | KM024238-240 | [1] |
|  |  |  | AC645 | 25 | KM024241-265 | [1] |
|  |  |  | AC601 | 3 | KM024266-268 | [1] |
|  |  | GOM |  | 2 | AB257532-33 | [2] |
|  |  |  | AC645 | 1 | AY649800^3^ | [3] |
|  |  |  |  | 1 | EF051246 | [4] |
|  |  |  | GC233 | 3 | EU288173-175^3^ | [5] |
|  | *B. mau* | WAF |  | 1 | AY649801 | [3] |
|  |  |  |  | 1 | EF051241^1^ | [4] |
|  |  | GOC |  | 11 | EU288159,161-165,167-168,170-172 | [5] |
|  |  | BAP |  | 16 | DQ513425-440^2^ | [6] |
|  |  |  |  | 1 | FJ890502 | [7] |
| ND4 | *B. chi* | GOM | GC234 | 10 | KM044648-657 | [1] |
|  |  |  | MC929 | 15 | KM044658-672 | [1] |
|  |  |  | GC233 | 10 | KM044673-682 | [1] |
|  |  |  | GC204 | 9 | KM044683-691 | [1] |
|  |  |  | GB697 | 4 | KM044692-695 | [1] |
|  |  |  | MC853 | 7 | KM044696-702 | [1] |
|  |  |  | MC640 | 2 | KM044703-704 | [1] |
|  |  |  | AC645 | 17 | KM044705-721 | [1] |
|  | *B. mau* |  | AC601 | 2 | KM044722-723 | [1] |
|  |  | WAF |  | 1 | AY649810 | [3] |
|  |  | GOC |  | 4 | EU288176-179 | [5] |

1. Faure B, Schaeffer SW, Fisher CR. Species Distribution and Population Connectivity of Deep-Sea Mussels at Hydrocarbon Seeps in the Gulf of Mexico. PLOS ONE. 2015;10(4). doi: ARTN e0118460 10.1371/journal.pone.0118460. PubMed PMID: ISI:000352590300004.

2. Miyazaki JI, Martins LD, Fujita Y, Matsumoto H, Fujiwara Y. Evolutionary Process of Deep-Sea *Bathymodiolus* Mussels. PLOS ONE. 2010;5(4). doi: ARTN e10363 10.1371/journal.pone.0010363. PubMed PMID: WOS:000277079700011.

3. Jones WJ, Won YJ, Maas PAY, Smith PJ, Lutz RA, Vrijenhoek RC. Evolution of habitat use by deep-sea mussels. Mar Biol. 2006;148(4):841-51. doi: 10.1007/s00227-005-0115-1. PubMed PMID: ISI:000235058600015.

4. Cordes EE, Carney SL, Hourdez S, Carney RS, Brooks JM, Fisher CR. Cold seeps of the deep Gulf of Mexico: Community structure and biogeographic comparisons to Atlantic equatorial belt seep communities. Deep-Sea Res Pt I. 2007;54(4):637-53. doi: DOI 10.1016/j.dsr.2007.01.001. PubMed PMID: ISI:000246382900010.

5. Genio L, Johnson SB, Vrijenhoek RC, Cunha MR, Tyler PA, Kiel S, et al. New record of "*Bathymodiolus*" *mauritanicus* Cosel 2002 from the Gulf of Cadiz (NE Atlantic) mud volcanoes. J Shellfish Res. 2008;27(1):53-61. doi: Doi 10.2983/0730-8000(2008)27[53:Nrobmc]2.0.Co;2. PubMed PMID: ISI:000254768000006.

6. Olu-Le Roy K, Cosel Rv, Hourdez S, Carney SL, Jollivet D. Amphi-Atlantic cold-seep *Bathymodiolus* species complexes across the equatorial belt. Deep-Sea Res Pt I. 2007;54(11):1890-911. doi: 10.1016/j.dsr.2007.07.004. PubMed PMID: ISI:000251665400003.

7. Lorion J, Buge B, Cruaud C, Samadi S. New insights into diversity and evolution of deep-sea Mytilidae (Mollusca: Bivalvia). Mol Phylogenet Evol. 2010;57(1):71-83. doi: 10.1016/j.ympev.2010.05.027. PubMed PMID: ISI:00028199290
