# Supplementary material for: Molecular characterization of Bathymodiolus mussels and gill symbionts associated with chemosynthetic habitats from the U.S. Atlantic margin
Source: PLoS One. 2019 Mar 14;14(3):e0211616. doi: 10.1371/journal.pone.0211616 (PMC6417655; doi:10.1371/journal.pone.0211616)
Supplement: S5 Table — V1-V8 are the variable regions within 16S (104) that are amplified by the primer sets. The final column contains the relative nucleotide (nt) position to E.coli 16S. * ps1, the universal Illumina primers, correspond to Bakt341F and Bakt805R (105). **The forward primer of primer set ps2 is a modified version of Bact27bF, (106) and the reverse primer is a modified version of 534R (107). (DOCX) [file pone.0211616.s010.docx]

Supplemental Table 5

| Primer Set | Forward | Reverse | V1 | V2 | V3 | V4 | V5 | V6 | V7 | V8 | Relative nt position |
| --- | --- | --- | --- | --- | --- | --- | --- | --- | --- | --- | --- |
| ps1* | CCTACGGGNGGCWGCAG | GACTACHVGGGTATCTAATCC |  |  | x | x |  |  |  |  | 341F, 805R |
| ps2** | AGAGTTTGATCMTGGCTCAGAKTG | CCGCGGCTGCTGGCACG | x | x | x |  |  |  |  |  | 27F, 534R |
| ps3 | CCGCGTGTGTGAAGAAGGC | CCGTCAATTCCTTTRAGTTT |  |  | x | x | x |  |  |  | 414F, 907R |
| ps4 | GGATTAGATACCCYGGTAGTCCAC | CRTTGTARCACGTGTGTAGCCC |  |  |  |  | x | x | x | x | 807F, 1269R |
