# Supplementary material for: Molecular characterization of Bathymodiolus mussels and gill symbionts associated with chemosynthetic habitats from the U.S. Atlantic margin
Source: PLoS One. 2019 Mar 14;14(3):e0211616. doi: 10.1371/journal.pone.0211616 (PMC6417655; doi:10.1371/journal.pone.0211616)
Supplement: S6 Table — (DOCX) [file pone.0211616.s011.docx]

Supplemental Table 6

| host species | GB accession # | type of symbiont | References |
| --- | --- | --- | --- |
| *B. aduloides* | KF657321.1 | thio | [1] |
| *B. aff. brevior* | DQ077891.1 | thio | [2] |
| *B. aff. brevior* | DQ321713.1 | thio | [3] |
| *B. aff. brevior* | KF521926.1 | thio | [1] |
| *B. aff. thermophilus* | DQ321717.1 | thio | [3] |
| *B. aff. thermophilus* | KF521929.1 | thio | [1] |
| *B. azoricus* | AY235676.1 | thio | [4] |
| *B. azoricus* | AY951931.1 | thio |  |
| *B. azoricus* | DQ321711.1 | thio | [3] |
| *B. azoricus* | DQ899960.1 | thio | [5] |
| *B. azoricus* | DQ899961.1 | thio | [5] |
| *B. azoricus* | DQ899962.1 | thio | [5] |
| *B. azoricus* | DQ899963.1 | thio | [5] |
| *B. azoricus* | DQ899964.1 | thio | [5] |
| *B. azoricus* | DQ899965.1 | thio | [5] |
| *B. azoricus* | DQ899966.1 | thio | [5] |
| *B. azoricus* | DQ899967.1 | thio | [5] |
| *B. azoricus* | DQ899968.1 | thio | [5] |
| *B. azoricus* | DQ899969.1 | thio | [5] |
| *B. azoricus* | DQ899970.1 | thio | [5] |
| *B. azoricus* | DQ899971.1 | thio | [5] |
| *B. azoricus* | DQ899972.1 | thio | [5] |
| *B. azoricus* | DQ899973.1 | thio | [5] |
| *B. azoricus* | DQ899974.1 | thio | [5] |
| *B. azoricus* | DQ899975.1 | thio | [5] |
| *B. azoricus* | DQ899976.1 | thio | [5] |
| *B. azoricus* | DQ899977.1 | thio | [5] |
| *B. azoricus* | DQ899978.1 | thio | [5] |
| *B. azoricus* | DQ899979.1 | thio | [5] |
| *B. azoricus* | DQ899980.1 | thio | [5] |
| *B. azoricus* | DQ899981.1 | thio | [5] |
| *B. azoricus* | DQ899982.1 | thio | [5] |
| *B. azoricus* | DQ899983.1 | thio | [5] |
| *B. azoricus* | DQ899984.1 | thio | [5] |
| *B. azoricus* | DQ899985.1 | thio | [5] |
| *B. azoricus* | DQ899986.1 | thio | [5] |
| *B. azoricus* | DQ899987.1 | thio | [5] |
| *B. azoricus* | DQ899988.1 | thio | [5] |
| *B. azoricus* | DQ899989.1 | thio | [5] |
| *B. azoricus* | DQ899990.1 | thio | [5] |
| *B. azoricus* | DQ899991.1 | thio | [5] |
| *B. azoricus* | DQ899992.1 | thio | [5] |
| *B. azoricus* | DQ899995.1 | thio | [5] |
| *B. azoricus* | DQ899996.1 | thio | [5] |
| *B. azoricus* | DQ899997.1 | thio | [5] |
| *B. azoricus* | DQ899998.1 | thio | [5] |
| *B. azoricus* | DQ899999.1 | thio | [5] |
| *B. azoricus* | DQ900005.1 | thio | [5] |
| *B. azoricus* | DQ900006.1 | thio | [5] |
| *B. azoricus* | DQ900007.1 | thio | [5] |
| *B. azoricus* | DQ900008.1 | thio | [5] |
| *B. azoricus* | DQ900009.1 | thio | [5] |
| *B. azoricus* | DQ900015.1 | thio | [5] |
| *B. azoricus* | DQ900016.1 | thio | [5] |
| *B. azoricus* | DQ900017.1 | thio | [5] |
| *B. azoricus* | DQ900018.1 | thio | [5] |
| *B. azoricus* | FR670517.1 | thio | [6] |
| *B. azoricus* | FR670518.1 | thio | [6] |
| *B. azoricus* | AY235678.1 | thio | [4] |
| *B. brevior* | DQ321714.1 | thio | [3] |
| *B. brevior* | KF521927.1 | thio | [1] |
| *B. brevior* | KF780847.1 | thio | [1] |
| *B. brevior* | KF780848.1 | thio | [1] |
| *B. brevior* | KF780849.1 | thio | [1] |
| *B. brooksi* | JF969168.1 | *Psychromonas* | [7] |
| *B. brooksi* | JF969169.1 | meth | [7] |
| *B. brooksi* | JF969170.1 | thio | [7] |
| *B. childressi* | AM236329.1 | meth | [8] |
| *B. heckerae* | JF969164.1 | *Cycloclasticus* | [7] |
| *B. heckerae* | JF969165.1 | meth | [7] |
| *B. heckerae* | JF969166.1 | thio | [7] |
| *B. heckerae* | JF969167.1 | thio | [7] |
| *B. hirtus* | AB250698.1 | meth |  |
| *B. hirtus* | AB250699.1 | unk |  |
| *B. hirtus* | AB250700.1 | thio |  |
| *B. japonicus* | AB036711.1 | meth | [9] |
| *B. marisindicus* | DQ321715.1 | thio | [3] |
| *B. marisindicus* | KF521930.1 | thio | [1] |
| *B. mauritanicus* | HE963013.1 | meth | [10] |
| *B. mauritanicus* | HE963014.1 | meth | [10] |
| *B. mauritanicus* | HE963019.1 | thio | [10] |
| *B. platifrons* | AB036710.1 | meth | [9] |
| *B. platifrons* | AB250696.1 | meth |  |
| *B. platifrons* | AB250697.1 | Campylobacterota |  |
| *B. puteoserpentis* | AY235677.1 | thio | [4] |
| *B. puteoserpentis* | AY235679.1 | thio | [4] |
| *B. puteoserpentis* | AY235680.1 | thio | [4] |
| *B. puteoserpentis* | DQ321712.1 | thio | [3] |
| *B. puteoserpentis* | DQ900010.1 | thio | [5] |
| *B. puteoserpentis* | DQ900011.1 | thio | [5] |
| *B. puteoserpentis* | DQ900012.1 | thio | [5] |
| *B. puteoserpentis* | DQ900013.1 | thio | [5] |
| *B. puteoserpentis* | DQ900014.1 | thio | [5] |
| *B. puteoserpentis* | DQ900019.1 | thio | [5] |
| *B. puteoserpentis* | DQ900020.1 | thio | [5] |
| *B. puteoserpentis* | DQ900021.1 | thio | [5] |
| *B. puteoserpentis* | DQ900022.1 | thio | [5] |
| *B. puteoserpentis* | DQ900023.1 | thio | [5] |
| *B. securiformis* | KF657322.1 | thio | [1] |
| *B. septemdierum* | AB036709.1 | thio | [9] |
| *B. septemdierum* | AB514582.1 | thio | [11] |
| *B. septemdierum* | AB514583.1 | thio | [11] |
| *B. septemdierum* | AB514584.1 | thio | [11] |
| *B. septemdierum* | AB598130.1 | thio | [12] |
| *B. thermophilus* | DQ321716.1 | thio | [3] |
| *B. thermophilus* | KF521928.1 | thio | [1] |
| *B. thermophilus* | M99445.1 | thio | [13] |
| *B. sp.* | AB056868.3 | meth | [14] |
| *B. sp.* | AB499796.1 | thio | [15] |
| *B. sp.* | AB499797.1 | thio | [15] |
| *B. sp.* | AM888203.1 | thio | [16] |
| *B. sp.* | AM888204.1 | meth | [16] |
| *B. sp.* | DQ899993.1 | thio | [5] |
| *B. sp.* | DQ899994.1 | thio | [5] |
| *B. sp.* | DQ900000.1 | thio | [5] |
| *B. sp.* | DQ900001.1 | thio | [5] |
| *B. sp.* | DQ900002.1 | thio | [5] |
| *B. sp.* | DQ900003.1 | thio | [5] |
| *B. sp.* | DQ900004.1 | thio | [5] |
| *B. sp.* | KF657323.1 | thio | [1] |
| *B. sp.* | KF657324.1 | thio | [1] |
| *B. sp.* | U05595.1 | meth | [17] |
| *B._sp.* | EU326223.1 | thio | [3] |
| *B._sp.* | FM244838.1 | Candidatus parasite | [18] |

1. Fontanez KM, Cavanaugh CM. Evidence for horizontal transmission from multilocus phylogeny of deep-sea mussel (Mytilidae) symbionts. Environ Microbiol. 2014;16(12):3608-21. doi: 10.1111/1462-2920.12379. PubMed PMID: 24428587.

2. McKiness ZP, Cavanaugh CM. The ubiquitous mussel: *Bathymodiolus aff. brevior* symbiosis at the Central Indian Ridge hydrothermal vents. Mar Ecol Prog Ser. 2005;295:183-90. doi: DOI 10.3354/meps295183. PubMed PMID: WOS:000230671800016.

3. Won Y-J, Jones WJ, Vrijenhoek RC. Absence of Cospeciation Between Deep-Sea Mytilids and Their Thiotrophic Endosymbionts. J Shellfish Res. 2008;27(1):129-38. doi: 10.2983/0730-8000(2008)27[129:aocbdm]2.0.co;2.

4. Won YJ, Hallam SJ, O'Mullan GD, Pan IL, Buck KR, Vrijenhoek RC. Environmental Acquisition of Thiotrophic Endosymbionts by Deep-Sea Mussels of the Genus *Bathymodiolus*. Appl Environ Microb. 2003;69(11):6785-92. doi: 10.1128/aem.69.11.6785-6792.2003.

5. DeChaine EG, Bates AE, Shank TM, Cavanaugh CM. Off-axis symbiosis found: Characterization and biogeography of bacterial symbionts of *Bathymodiolus* mussels from Lost City hydrothermal vents. Environ Microbiol. 2006;8(11):1902-12. doi: 10.1111/j.1462-2920.2005.01113.x. PubMed PMID: 17014490.

6. Crepeau V, Cambon Bonavita MA, Lesongeur F, Randrianalivelo H, Sarradin PM, Sarrazin J, et al. Diversity and function in microbial mats from the Lucky Strike hydrothermal vent field. FEMS Microbiol Ecol. 2011;76(3):524-40. doi: 10.1111/j.1574-6941.2011.01070.x. PubMed PMID: 21348883.

7. Raggi L, Schubotz F, Hinrichs KU, Dubilier N, Petersen JM. Bacterial symbionts of *Bathymodiolus* mussels and *Escarpia* tubeworms from Chapopote, an asphalt seep in the southern Gulf of Mexico. Environ Microbiol. 2013;15(7):1969-87. doi: 10.1111/1462-2920.12051. PubMed PMID: WOS:000328955900005.

8. Duperron S, Fiala-Medioni A, Caprais JC, Olu K, Sibuet M. Evidence for chemoautotrophic symbiosis in a Mediterranean cold seep clam (Bivalvia : Lucinidae): comparative sequence analysis of bacterial 16S rRNA, APS reductase and RubisCO genes. Fems Microbiology Ecology. 2007;59(1):64-70. doi: 10.1111/j.1574-6941.2006.00194.x. PubMed PMID: WOS:000242784700007.

9. Fujiwara Y, Kawato M, Noda C, Kinoshita G, Yamanaka T, Fujita Y, et al. Extracellular and mixotrophic symbiosis in the whale-fall mussel *Adipicola pacifica*: a trend in evolution from extra- to intracellular symbiosis. Plos One. 2010;5(7):e11808. doi: 10.1371/journal.pone.0011808. PubMed PMID: 20676405; PubMed Central PMCID: PMCPMC2910738.

10. Rodrigues CF, Hilário A, Cunha MR. Chemosymbiotic species from the Gulf of Cadiz (NE Atlantic): distribution, life styles and nutritional patterns. Biogeosciences. 2013;10(4):2569-81. doi: 10.5194/bg-10-2569-2013.

11. Fujinoki M, Koito T, Nemoto S, Kitada M, Yamaguchi Y, Hyodo S, et al. Comparison of the amount of thiotrophic symbionts in the deep-sea mussel *Bathymodiolus septemdierum* under different sulfide levels using fluorescent in situ hybridization. Fisheries Sci. 2012;78(1):139-46. doi: 10.1007/s12562-011-0419-7. PubMed PMID: WOS:000299038500016.

12. Kuwahara H, Takaki Y, Shimamura S, Yoshida T, Maeda T, Kunieda T, et al. Loss of genes for DNA recombination and repair in the reductive genome evolution of thioautotrophic symbionts of *Calyptogena* clams. Bmc Evol Biol. 2011;11. doi: Artn 285 10.1186/1471-2148-11-285. PubMed PMID: WOS:000296283600001.

13. Distel DL, Lane DJ, Olsen GJ, Giovannoni SJ, Pace B, Pace NR, et al. Sulfur-Oxidizing Bacterial Endosymbionts - Analysis of Phylogeny and Specificity by 16s Ribosomal-Rna Sequences. J Bacteriol. 1988;170(6):2506-10. PubMed PMID: WOS:A1988N699900014.

14. Elsaied HE, Kaneko R, Naganuma T. Molecular characterization of a deep-sea methanotrophic mussel symbiont that carries a RuBisCO gene. Mar Biotechnol (NY). 2006;8(5):511-20. doi: 10.1007/s10126-005-6135-5. PubMed PMID: 16761196.

15. Nishijima M, Lindsay DJ, Hata J, Nakamura A, Kasai H, Ise Y, et al. Association of Thioautotrophic Bacteria with Deep-Sea Sponges. Mar Biotechnol. 2010;12(3):253-60. doi: 10.1007/s10126-009-9253-7. PubMed PMID: WOS:000278095400002.

16. Cambon-Bonavita MA, Nadalig T, Roussel E, Delage E, Duperron S, Caprais JC, et al. Diversity and distribution of methane-oxidizing microbial communities associated with different faunal assemblages in a giant pockmark of the Gabon continental margin. Deep-Sea Res Pt Ii. 2009;56(23):2248-58. doi: 10.1016/j.dsr2.2009.04.007. PubMed PMID: WOS:000272366000009.

17. Distel DL, Cavanaugh CM. Independent Phylogenetic Origins of Methanotrophic and Chemoautotrophic Bacterial Endosymbioses in Marine Bivalves. J Bacteriol. 1994;176(7):1932-8. PubMed PMID: WOS:A1994ND18300016.

18. Zielinski FU, Pernthaler A, Duperron S, Raggi L, Giere O, Borowski C, et al. Widespread occurrence of an intranuclear bacterial parasite in vent and seep bathymodiolin mussels. Environ Microbiol. 2009;11(5):1150-67. doi: 10.1111/j.1462-2920.2008.01847.x. PubMed PMID: WOS:000265481600011.
