# Supplementary material for: Molecular characterization of Bathymodiolus mussels and gill symbionts associated with chemosynthetic habitats from the U.S. Atlantic margin
Source: PLoS One. 2019 Mar 14;14(3):e0211616. doi: 10.1371/journal.pone.0211616 (PMC6417655; doi:10.1371/journal.pone.0211616)
Supplement: S7 Table — Contig = merged read pair from MiSeq amplicon data. ps1 = primer set 1, ps2 = primer set 2, Search database was NCBI nucleotide (nt) database, 7/11/17 download date. eval, bit, id%, Top Hit are outputs from Blast searches explained on NCBI’s website: https://www.ncbi.nlm.nih.gov/BLAST/tutorial/Altschul-1.html. Note that JQ844779 is 99% identical with 100% query coverage to AM236329. (DOCX) [file pone.0211616.s012.docx]

Supplemental Table 7

| contig | primer | eval | bit | id% | Top hit |
| --- | --- | --- | --- | --- | --- |
| M02740_42_000000000-AFFVB_1_1102_18288_20930 | ps1 | 0 | 810 | 99% | gb\|JQ844779 |
| M02740_42_000000000-AFFVB_1_1105_19728_10088 | ps1 | 0 | 810 | 99% | gb\|JQ844779 |
| M02740_42_000000000-AFFVB_1_1103_28572_18526 | ps1 | 0 | 792 | 98% | gb\|JQ844779 |
| M02740_42_000000000-AFFVB_1_1106_7540_2357 | ps1 | 0 | 796 | 98% | gb\|JQ844779 |
| M02740_42_000000000-AFFVB_1_1107_17216_9595 | ps1 | 0 | 783 | 98% | gb\|JQ844779 |
| M02740_42_000000000-AFFVB_1_1108_15243_2887 | ps1 | 0 | 798 | 98% | gb\|JQ844779 |
| M02740_42_000000000-AFFVB_1_1109_5861_17372 | ps1 | 0 | 792 | 98% | gb\|JQ844779 |
| M02740_42_000000000-AFFVB_1_1110_24910_12496 | ps1 | 0 | 801 | 98% | gb\|JQ844779 |
| M02740_42_000000000-AFFVB_1_1111_6229_7098 | ps1 | 0 | 796 | 98% | gb\|JQ844779 |
| M02740_42_000000000-AFFVB_1_1111_23071_16604 | ps1 | 0 | 803 | 98% | gb\|JQ844779 |
| M02740_42_000000000-AFFVB_1_1112_26942_21097 | ps1 | 0 | 778 | 98% | gb\|JQ844779 |
| M02740_42_000000000-AFFVB_1_1114_14908_19255 | ps1 | 0 | 801 | 98% | gb\|JQ844779 |
| M02740_42_000000000-AFFVB_1_1115_25376_13822 | ps1 | 0 | 791 | 98% | gb\|JQ844779 |
| M02740_42_000000000-AFFVB_1_1116_7519_8942 | ps1 | 0 | 785 | 98% | gb\|JQ844779 |
| M02740_42_000000000-AFFVB_1_1117_14098_2597 | ps1 | 0 | 798 | 98% | gb\|JQ844779 |
| M02740_42_000000000-AFFVB_1_1117_19536_20323 | ps1 | 0 | 791 | 98% | gb\|JQ844779 |
| M02740_42_000000000-AFFVB_1_1119_5706_21853 | ps1 | 0 | 792 | 98% | gb\|JQ844779 |
| M02740_42_000000000-AFFVB_1_2101_17183_18774 | ps1 | 0 | 796 | 98% | gb\|JQ844779 |
| M02740_42_000000000-AFFVB_1_2103_25287_21978 | ps1 | 0 | 791 | 98% | gb\|JQ844779 |
| M02740_42_000000000-AFFVB_1_2105_18188_13971 | ps1 | 0 | 801 | 98% | gb\|JQ844779 |
| M02740_42_000000000-AFFVB_1_2108_14255_20108 | ps1 | 0 | 805 | 98% | gb\|JQ844779 |
| M02740_42_000000000-AFFVB_1_2109_9460_9025 | ps1 | 0 | 796 | 98% | gb\|JQ844779 |
| M02740_42_000000000-AFFVB_1_2109_11523_18554 | ps1 | 0 | 801 | 98% | gb\|JQ844779 |
| M02740_42_000000000-AFFVB_1_2110_24332_5680 | ps1 | 0 | 801 | 98% | gb\|JQ844779 |
| M02740_42_000000000-AFFVB_1_2110_19804_18666 | ps1 | 0 | 801 | 98% | gb\|JQ844779 |
| M02740_42_000000000-AFFVB_1_2111_29002_8911 | ps1 | 0 | 796 | 98% | gb\|JQ844779 |
| M02740_42_000000000-AFFVB_1_2112_6151_10761 | ps1 | 0 | 801 | 98% | gb\|JQ844779 |
| M02740_42_000000000-AFFVB_1_2113_24920_3627 | ps1 | 0 | 792 | 98% | gb\|JQ844779 |
| M02740_42_000000000-AFFVB_1_2113_12469_13431 | ps1 | 0 | 796 | 98% | gb\|JQ844779 |
| M02740_42_000000000-AFFVB_1_2113_23962_22885 | ps1 | 0 | 801 | 98% | gb\|JQ844779 |
| M02740_42_000000000-AFFVB_1_2114_6583_12353 | ps1 | 0 | 801 | 98% | gb\|JQ844779 |
| M02740_42_000000000-AFFVB_1_2114_6273_22363 | ps1 | 0 | 792 | 98% | gb\|JQ844779 |
| M02740_42_000000000-AFFVB_1_1112_22730_4213 | ps1 | 0 | 776 | 97% | gb\|JQ844779 |
| M02740_42_000000000-AFFVB_1_1113_7991_12764 | ps1 | 0 | 774 | 97% | gb\|JQ844779 |
| M02740_42_000000000-AFFVB_1_1114_5023_5664 | ps1 | 0 | 769 | 97% | gb\|JQ844779 |
| M02740_42_000000000-AFFVB_1_1119_11964_9001 | ps1 | 0 | 774 | 97% | gb\|JQ844782 |
| M02740_42_000000000-AFFVB_1_2103_22485_8427 | ps1 | 0 | 773 | 97% | gb\|JQ844779 |
| M02740_42_000000000-AFFVB_1_2106_18144_5763 | ps1 | 0 | 769 | 97% | gb\|JQ844779 |
| M02740_42_000000000-AFFVB_1_2106_22027_16162 | ps1 | 0 | 765 | 97% | gb\|JQ844779 |
| M02740_42_000000000-AFFVB_1_2107_26146_18483 | ps1 | 0 | 773 | 97% | gb\|JQ844779 |
| M02740_42_000000000-AFFVB_1_2111_19525_21687 | ps1 | 0 | 783 | 97% | gb\|JQ844779 |
| M02740_42_000000000-AFFVB_1_1104_2388_14028 | ps1 | 0 | 742 | 96% | gb\|JQ844779 |
| M02740_42_000000000-AFFVB_1_2104_25559_18355 | ps1 | 0 | 742 | 96% | gb\|JQ844779 |
| M02740_42_000000000-AFFVB_1_2107_26002_7201 | ps1 | 0 | 747 | 96% | gb\|JQ844779 |
| M02740_42_000000000-AFFVB_1_1118_9314_14643 | ps1 | 0 | 733 | 95% | gb\|JQ844779 |
| M02740_42_000000000-AFFVB_1_2102_14248_14278 | ps1 | 0 | 713 | 95% | gb\|JQ844779 |
| M02740_42_000000000-AFFVB_1_2108_25701_9198 | ps1 | 0 | 729 | 95% | gb\|JQ844779 |
| M02740_42_000000000-AFFVB_1_1101_12672_19567 | ps1 | 0 | 688 | 94% | gb\|JQ844779 |
| M02740_42_000000000-AFFVB_1_1106_25131_18520 | ps1 | 0 | 715 | 94% | gb\|JQ844779 |
| M02740_42_000000000-AFFVB_1_1108_23818_20637 | ps1 | 0 | 691 | 94% | emb\|AM236329 |
| M02740_42_000000000-AFFVB_1_1101_7507_23206 | ps2 | 0 | 902 | 99% | emb\|AM236329 |
| M02740_42_000000000-AFFVB_1_1102_17474_19813 | ps2 | 0 | 902 | 99% | emb\|AM236329 |
| M02740_42_000000000-AFFVB_1_1105_7905_12899 | ps2 | 0 | 902 | 99% | emb\|AM236329 |
| M02740_42_000000000-AFFVB_1_1107_25819_7770 | ps2 | 0 | 908 | 99% | emb\|AM236329 |
| M02740_42_000000000-AFFVB_1_1108_27197_20900 | ps2 | 0 | 902 | 99% | emb\|AM236329 |
| M02740_42_000000000-AFFVB_1_1113_26682_9727 | ps2 | 0 | 908 | 99% | emb\|AM236329 |
| M02740_42_000000000-AFFVB_1_1114_23408_4768 | ps2 | 0 | 902 | 99% | emb\|AM236329 |
| M02740_42_000000000-AFFVB_1_1114_20259_20438 | ps2 | 0 | 902 | 99% | emb\|AM236329 |
| M02740_42_000000000-AFFVB_1_2101_25078_17778 | ps2 | 0 | 902 | 99% | emb\|AM236329 |
| M02740_42_000000000-AFFVB_1_2109_4712_16726 | ps2 | 0 | 899 | 99% | emb\|AM236329 |
| M02740_42_000000000-AFFVB_1_2111_22475_16830 | ps2 | 0 | 908 | 99% | emb\|AM236329 |
| M02740_42_000000000-AFFVB_1_2112_24393_8380 | ps2 | 0 | 908 | 99% | emb\|AM236329 |
| M02740_42_000000000-AFFVB_1_2112_26643_18170 | ps2 | 0 | 908 | 99% | emb\|AM236329 |
| M02740_42_000000000-AFFVB_1_2113_23963_9327 | ps2 | 0 | 908 | 99% | emb\|AM236329 |
| M02740_42_000000000-AFFVB_1_2113_19963_17319 | ps2 | 0 | 902 | 99% | emb\|AM236329 |
| M02740_42_000000000-AFFVB_1_1106_27582_10014 | ps2 | 0 | 893 | 98% | emb\|AM236329 |
| M02740_42_000000000-AFFVB_1_1110_9939_17002 | ps2 | 0 | 893 | 98% | emb\|AM236329 |
| M02740_42_000000000-AFFVB_1_1111_3076_10829 | ps2 | 0 | 899 | 98% | emb\|AM236329 |
| M02740_42_000000000-AFFVB_1_1111_22049_19686 | ps2 | 0 | 881 | 98% | emb\|AM236329 |
| M02740_42_000000000-AFFVB_1_1112_14928_17616 | ps2 | 0 | 893 | 98% | emb\|AM236329 |
| M02740_42_000000000-AFFVB_1_1116_9846_11486 | ps2 | 0 | 893 | 98% | emb\|AM236329 |
| M02740_42_000000000-AFFVB_1_1117_6236_7727 | ps2 | 0 | 899 | 98% | emb\|AM236329 |
| M02740_42_000000000-AFFVB_1_1117_18082_24234 | ps2 | 0 | 895 | 98% | emb\|AM236329 |
| M02740_42_000000000-AFFVB_1_2101_22654_6740 | ps2 | 0 | 899 | 98% | emb\|AM236329 |
| M02740_42_000000000-AFFVB_1_2103_11392_8801 | ps2 | 0 | 899 | 98% | emb\|AM236329 |
| M02740_42_000000000-AFFVB_1_2104_23631_17184 | ps2 | 0 | 893 | 98% | emb\|AM236329 |
| M02740_42_000000000-AFFVB_1_2105_8771_12804 | ps2 | 0 | 899 | 98% | emb\|AM236329 |
| M02740_42_000000000-AFFVB_1_2107_17429_18912 | ps2 | 0 | 890 | 98% | emb\|AM236329 |
| M02740_42_000000000-AFFVB_1_2108_22198_13708 | ps2 | 0 | 890 | 98% | emb\|AM236329 |
| M02740_42_000000000-AFFVB_1_2109_6710_6374 | ps2 | 0 | 872 | 98% | emb\|AM236329 |
| M02740_42_000000000-AFFVB_1_2110_12458_7875 | ps2 | 0 | 893 | 98% | emb\|AM236329 |
| M02740_42_000000000-AFFVB_1_2110_9377_18840 | ps2 | 0 | 899 | 98% | emb\|AM236329 |
| M02740_42_000000000-AFFVB_1_2111_9645_8386 | ps2 | 0 | 890 | 98% | emb\|AM236329 |
| M02740_42_000000000-AFFVB_1_2114_8320_6473 | ps2 | 0 | 884 | 98% | emb\|AM236329 |
| M02740_42_000000000-AFFVB_1_2115_9910_6179 | ps2 | 0 | 893 | 98% | emb\|AM236329 |
| M02740_42_000000000-AFFVB_1_2115_16033_14815 | ps2 | 0 | 892 | 98% | emb\|AM236329 |
| M02740_42_000000000-AFFVB_1_2116_10099_2479 | ps2 | 0 | 899 | 98% | emb\|AM236329 |
| M02740_42_000000000-AFFVB_1_2116_21483_12978 | ps2 | 0 | 884 | 98% | emb\|AM236329 |
| M02740_42_000000000-AFFVB_1_1103_8049_17817 | ps2 | 0 | 852 | 97% | emb\|AM236329 |
| M02740_42_000000000-AFFVB_1_1104_29655_13723 | ps2 | 0 | 850 | 97% | emb\|AM236329 |
| M02740_42_000000000-AFFVB_1_1108_8258_5450 | ps2 | 0 | 872 | 97% | emb\|AM236329 |
| M02740_42_000000000-AFFVB_1_1109_25244_19969 | ps2 | 0 | 868 | 97% | emb\|AM236329 |
| M02740_42_000000000-AFFVB_1_1118_23044_18252 | ps2 | 0 | 857 | 97% | emb\|AM236329 |
| M02740_42_000000000-AFFVB_1_1119_8087_14370 | ps2 | 0 | 875 | 97% | emb\|AM236329 |
| M02740_42_000000000-AFFVB_1_2102_20473_12597 | ps2 | 0 | 740 | 97% | emb\|AM236329 |
| M02740_42_000000000-AFFVB_1_2104_21797_3337 | ps2 | 0 | 859 | 97% | emb\|AM236329 |
| M02740_42_000000000-AFFVB_1_2106_11108_11163 | ps2 | 0 | 854 | 97% | emb\|AM236329 |
| M02740_42_000000000-AFFVB_1_2107_21482_8058 | ps2 | 0 | 868 | 97% | emb\|AM236329 |
| M02740_42_000000000-AFFVB_1_1115_9875_16656 | ps2 | 0 | 830 | 96% | emb\|AM236329 |
| M02740_42_000000000-AFFVB_1_2114_23375_16789 | ps2 | 0 | 782 | 93% | emb\|AM236329 |
