# Supplementary material for: Molecular characterization of Bathymodiolus mussels and gill symbionts associated with chemosynthetic habitats from the U.S. Atlantic margin
Source: PLoS One. 2019 Mar 14;14(3):e0211616. doi: 10.1371/journal.pone.0211616 (PMC6417655; doi:10.1371/journal.pone.0211616)
Supplement: S8 Table — (DOCX) [file pone.0211616.s013.docx]

Supplemental Table 8

| GB Accession # | Figure | Host/environment | Reference |
| --- | --- | --- | --- |
| AM083958 | 4 | *B. puteoserpentis* | [1] |
| AM083965 | 4 | *B. puteoserpentis* | [1] |
| AM083961 | 4 | *B. puteoserpentis* | [1] |
| AM083959 | 4 | *B. puteoserpentis* | [1] |
| AM083956 | 4 | *B. puteoserpentis* | [1] |
| AM083966 | 4 | *B. puteoserpentis* | [1] |
| AM083957 | 4 | *B. puteoserpentis* | [1] |
| AM083960 | 4 | *B. puteoserpentis* | [1] |
| AM083963 | 4 | *B. puteoserpentis* | [1] |
| AM083951 | 4 | *B. azoricus* | [1] |
| AM083955 | 4 | *B. puteoserpentis* | [1] |
| AM083962 | 4 | *B. puteoserpentis* | [1] |
| AM083964 | 4 | *B. puteoserpentis* | [1] |
| AM083952 | 4 | *B. azoricus* | [1] |
| AM083953 | 4 | *B. azoricus* | [1] |
| AM083954 | 4 | *B. azoricus* | [1] |
| AM083950 | 4 | *B. azoricus* | [1] |
| JF969169 | 4 | *B. brooksi* | [2] |
| AM236330 | 4 | *B. brooksi* | [3] |
| AM402955 | 4 | *Idas sp.* | [4] |
| AM083967 | 4 | *B. azoricus* | [1] |
| KF657324 | 4 | *B sp. Sissano1* | [5] |
| JF969165 | 4 | *B. heckerae* | [2] |
| AM236325 | 4 | *B. heckerae* | [3] |
| AJ745717 | 4 | *B. sp. Gabon* | [6] |
| AB036711 | 4 | *B. japonicus* | [7] |
| AB250696 | 4 | *B. platifrons* |  |
| AM236329 | 4 | *B. childressi* | [3] |
| KF657322 | 4 | *B. securiformis* | [5] |
| JQ844775 | 4 | *B. sp. 9 South* | [8] |
| JQ844782 | 4 | *B. sp. 9 South* | [8] |
| JQ844779 | 4 | *B. sp. 9 South* | [8] |
| AB250698 | 4 | *B. hirtus* |  |
| FN822778 | 4 | *B. mauritanicus* | [9] |
| HE963013 | 4 | *B. mauritanicus* | [10] |
| HE963014 | 4 | *B. mauritanicus* | [10] |
| KU573851 | 5 | *B. azoricus* | [11] |
| KU573852 | 5 | *B. azoricus* | [11] |
| KU573853 | 5 | *B. childressi* | [11] |
| KU573854 | 5 | *B. childressi* | [11] |
| KU573855 | 5 | *B. childressi* | [11] |
| KU573856 | 5 | *B. childressi* | [11] |
| KU573857 | 5 | *B. childressi* | [11] |
| KU573858 | 5 | *B. childressi* | [11] |
| KU573859 | 5 | *B. childressi* | [11] |
| KU573860 | 5 | *B. childressi* | [11] |
| KU573861 | 5 | *B. childressi* | [11] |
| KU573862 | 5 | *B. childressi* | [11] |
| KU573863 | 5 | *B. childressi* | [11] |
| KU573864 | 5 | *B. childressi* | [11] |
| KU573848 | 5 | *B. azoricus* | [11] |
| KU573865 | 5 | *B. childressi* | [11] |
| KU573850 | 5 | *B. azoricus* | [11] |
| KU573866 | 5 | *B. childressi* | [11] |
| KU573879 | 5 | *B. sp. Pakistan* | [11] |
| KU573880 | 5 | *B. sp. Pakistan* | [11] |
| KU573870 | 5 | *B. manuensis* | [11] |
| KU573871 | 5 | *B. manuensis* | [11] |
| KU573872 | 5 | *B. manuensis* | [11] |
| KU573873 | 5 | *B. manuensis* | [11] |
| AB250697 | 5 | *B. platifrons* |  |
| KU573874 | 5 | *B. mauritanicus* | [11] |
| KU573875 | 5 | *B. mauritanicus* | [11] |
| KU573876 | 5 | *B. mauritanicus* | [11] |
| KU644660 | 5 | *B. sp. 9 South* | [11] |
| KU573849 | 5 | *B. azoricus* | [11] |
| KU573867 | 5 | *B. childressi* | [11] |
| KU573868 | 5 | *B. childressi* | [11] |
| KU573869 | 5 | *B. childressi* | [11] |
| KU573877 | 5 | *B. azoricus* | [11] |
| KU573878 | 5 | *B. childressi* | [11] |
| KU573847 | 5 | *B. azoricus* | [11] |
| KU644654 | 5 | *B. childressi* | [11] |
| KU644649 | 5 | *B. childressi* | [11] |
| KU573846 | 5 | *B. azoricus* | [11] |
| KU644651 | 5 | *B. childressi* | [11] |
| KU644650 | 5 | *B. childressi* | [11] |
| KU644652 | 5 | *B. azoricus* | [11] |
| KU644658 | 5 | *B. sp. 9 South* | [11] |
| KU644648 | 5 | *B. childressi* | [11] |
| KU644647 | 5 | *B. azoricus* | [11] |
| KU644655 | 5 | *B. sp. 9 South* | [11] |
| KU644659 | 5 | *B. sp. 9 South* | [11] |
| KU644656 | 5 | *B. sp. 9 South* | [11] |
| KU644653 | 5 | *B. childressi* | [11] |
| KU644646 | 5 | *B. azoricus* | [11] |
| KU644657 | 5 | *B. sp. 9 South* | [11] |
| FM994659 | 5 | *Pectinodonta sp.* | [12] |
| FM994669 | 5 | *Pectinodonta sp.* | [12] |
| FN600361 | 5 | *Thyasira flexuosa* | [13] |
| DQ917867 | 5 | *Muricea elongata* |  |
| GU117948 | 5 | *Acropora cervicornis* | [14] |
| FM203377 | 5 | *Rimicaris exoculata* | [15] |
| HQ393439 | 5 | *Haliotis diversicolor* |  |
| JN255994 | 5 | *Kiwa puravida* | [16] |
| FM203395 | 5 | *Rimicaris exoculata* | [15] |
| FN658695 | 5 | *Rimicaris exoculata* | [17] |
| AY531574 | 5 | vent gastropod | [18] |
| NR_024802 | 5 | hydrothermal vent sediment | [19] |
| NR_119304 | 5 | human | [20] |
| BX571660 | 5 | bovine | [21] |
| NR_116342 | 5 | seawater, starfish, seaweed | [22] |
| NR_117760 | 5 | birds |  |
| NR_026422 | 5 | marine surface sediment | [23] |
| NR_043780 | 5 | hypersaline lake | [24] |

1. Duperron S, Bergin C, Zielinski F, Blazejak A, Pernthaler A, McKiness ZP, et al. A dual symbiosis shared by two mussel species, *Bathymodiolus azoricus* and *Bathymodiolus puteoserpentis* (Bivalvia : Mytilidae), from hydrothermal vents along the northern Mid-Atlantic Ridge. Environ Microbiol. 2006;8(8):1441-7. doi: 10.1111/j.1462-2920.2006.01038.x. PubMed PMID: WOS:000238885300012.

2. Raggi L, Schubotz F, Hinrichs KU, Dubilier N, Petersen JM. Bacterial symbionts of *Bathymodiolus* mussels and *Escarpia* tubeworms from Chapopote, an asphalt seep in the southern Gulf of Mexico. Environ Microbiol. 2013;15(7):1969-87. doi: 10.1111/1462-2920.12051. PubMed PMID: WOS:000328955900005.

3. Duperron S, Fiala-Medioni A, Caprais JC, Olu K, Sibuet M. Evidence for chemoautotrophic symbiosis in a Mediterranean cold seep clam (Bivalvia : Lucinidae): comparative sequence analysis of bacterial 16S rRNA, APS reductase and RubisCO genes. Fems Microbiology Ecology. 2007;59(1):64-70. doi: 10.1111/j.1574-6941.2006.00194.x. PubMed PMID: WOS:000242784700007.

4. Duperron S, Halary S, Lorion J, Sibuet M, Gaill F. Unexpected co-occurrence of six bacterial symbionts in the gills of the cold seep mussel *Idas sp* (Bivalvia : Mytilidae). Environ Microbiol. 2008;10(2):433-45. doi: 10.1111/j.1462-2920.2007.01465.x. PubMed PMID: WOS:000252320800014.

5. Lorion J, Kiel S, Faure B, Kawato M, Ho SY, Marshall B, et al. Adaptive radiation of chemosymbiotic deep-sea mussels. Proc Biol Sci. 2013;280(1770):20131243. doi: 10.1098/rspb.2013.1243. PubMed PMID: 24048154; PubMed Central PMCID: PMCPMC3779325.

6. Duperron S, Nadalig T, Caprais JC, Sibuet M, Fiala-Medioni A, Amann R, et al. Dual symbiosis in a *Bathymodiolus sp* mussel from a methane seep on the gabon continental margin (southeast Atlantic): 16S rRNA phylogeny and distribution of the symbionts in gills. Appl Environ Microb. 2005;71(4):1694-700. doi: Doi 10.1128/Aem.71.4.1694-1700.2005. PubMed PMID: WOS:000228338000003.

7. Fujiwara Y, Kawato M, Noda C, Kinoshita G, Yamanaka T, Fujita Y, et al. Extracellular and mixotrophic symbiosis in the whale-fall mussel *Adipicola pacifica*: a trend in evolution from extra- to intracellular symbiosis. Plos One. 2010;5(7):e11808. doi: 10.1371/journal.pone.0011808. PubMed PMID: 20676405; PubMed Central PMCID: PMCPMC2910738.

8. van der Heijden K, Petersen JM, Dubilier N, Borowski C. Genetic Connectivity between North and South Mid-Atlantic Ridge Chemosynthetic Bivalves and Their Symbionts. Plos One. 2012;7(7). doi: ARTN e39994 10.1371/journal.pone.0039994. PubMed PMID: ISI:000306461800030.

9. Rodrigues CF, Webster G, Cunha MR, Duperron S, Weightman AJ. Chemosynthetic bacteria found in bivalve species from mud volcanoes of the Gulf of Cadiz. Fems Microbiology Ecology. 2010;73(3):486-99. doi: 10.1111/j.1574-6941.2010.00913.x. PubMed PMID: WOS:000280633000007.

10. Rodrigues CF, Hilário A, Cunha MR. Chemosymbiotic species from the Gulf of Cadiz (NE Atlantic): distribution, life styles and nutritional patterns. Biogeosciences. 2013;10(4):2569-81. doi: 10.5194/bg-10-2569-2013.

11. Assie A, Borowski C, van der Heijden K, Raggi L, Geier B, Leisch N, et al. A specific and widespread association between deep-sea *Bathymodiolus* mussels and a novel family of Epsilonproteobacteria. Env Microbiol Rep. 2016;8(5):805-13. doi: 10.1111/1758-2229.12442. PubMed PMID: WOS:000395002300035.

12. Zbinden M, Pailleret M, Ravaux J, Gaudron SM, Hoyoux C, Lambourdiere J, et al. Bacterial communities associated with the wood-feeding gastropod *Pectinodonta sp* (Patellogastropoda, Mollusca). Fems Microbiology Ecology. 2010;74(2):450-63. doi: 10.1111/j.1574-6941.2010.00959.x. PubMed PMID: WOS:000282883200017.

13. Brissac T, Rodrigues CF, Gros O, Duperron S. Characterization of bacterial symbioses in *Myrtea* sp. (Bivalvia: Lucinidae) and *Thyasira* sp. (Bivalvia: Thyasiridae) from a cold seep in the Eastern Mediterranean. Mar Ecol-Evol Persp. 2011;32(2):198-210. doi: 10.1111/j.1439-0485.2010.00413.x. PubMed PMID: WOS:000289728800007.

14. Sunagawa S, Woodley CM, Medina M. Threatened Corals Provide Underexplored Microbial Habitats. Plos One. 2010;5(3). doi: ARTN e9554 10.1371/journal.pone.0009554. PubMed PMID: WOS:000275197200009.

15. Petersen JM, Ramette A, Lott C, Cambon-Bonavita MA, Zbinden M, Dubilier N. Dual symbiosis of the vent shrimp *Rimicaris exoculata* with filamentous gamma- and epsilonproteobacteria at four Mid-Atlantic Ridge hydrothermal vent fields. Environ Microbiol. 2010;12(8):2204-18. doi: 10.1111/j.1462-2920.2009.02129.x. PubMed PMID: WOS:000280652500014.

16. Thurber AR, Jones WJ, Schnabel K. Dancing for Food in the Deep Sea: Bacterial Farming by a New Species of Yeti Crab. Plos One. 2011;6(11). PubMed PMID: WOS:000298168100003.

17. Hugler M, Petersen JM, Dubilier N, Imhoff JF, Sievert SM. Pathways of Carbon and Energy Metabolism of the Epibiotic Community Associated with the Deep-Sea Hydrothermal Vent Shrimp *Rimicaris exoculata*. Plos One. 2011;6(1). doi: ARTN e16018 10.1371/journal.pone.0016018. PubMed PMID: WOS:000286512900018.

18. Goffredi SK, Waren A, Orphan VJ, Van Dover CL, Vrijenhoek RC. Novel forms of structural integration between microbes and a hydrothermal vent gastropod from the Indian Ocean. Appl Environ Microb. 2004;70(5):3082-90. doi: 10.1128/Aem.70.5.3082-3090.2004. PubMed PMID: ISI:000221340400066.

19. Inagaki F, Takai K, Nealson KH, Horikoshi K. *Sulfurovum lithotrophicum* gen. nov., sp nov., a novel sulfur-oxidizing chemolithoautotroph within the epsilon-Proteobacteria isolated from Okinawa Trough hydrothermal sediments. International Journal of Systematic and Evolutionary Microbiology. 2004;54:1477-82. doi: 10.1099/ijs.0.03042-0. PubMed PMID: WOS:000224259100008.

20. Khan MMA, Stoker NG, Drasar BS. Sequence diversity of a fragment of the 16S RNA gene from *Helicobacter pylori*. Microbios. 2000;103(406):139-50. PubMed PMID: WOS:000165102200001.

21. Baar C, Eppinger M, Raddatz G, Simon J, Lanz C, Klimmek O, et al. Complete genome sequence and analysis of *Wolinella succinogenes*. P Natl Acad Sci USA. 2003;100(20):11690-5. doi: 10.1073/pnas.1932838100. PubMed PMID: WOS:000185685700092.

22. Kim HM, Hwang CY, Cho BC. *Arcobacter marinus* sp nov. International Journal of Systematic and Evolutionary Microbiology. 2010;60:531-6. doi: 10.1099/ijs.0.007740-0. PubMed PMID: WOS:000276328700010.

23. Finster K, Liesack W, Tindall BJ. *Sulfurospirillum arcachonense* sp. nov., a new-microaerophilic sulfur-reducing bacterium. Int J Syst Bacteriol. 1997;47(4):1212-7. PubMed PMID: WOS:A1997YB18300045.

24. Sorokin DY, Tourova TP, Kolganova TV, Spiridonova EM, Berg IA, Muyzer G. *Thiomicrospira halophila* sp nov., a moderately halophilic, obligately chemolithoautotrophic, sulfur-oxidizing bacterium from hypersaline lakes. International Journal of Systematic and Evolutionary Microbiology. 2006;56:2375-80. doi: 10.1099/ijs.0.64445-0. PubMed PMID: WOS:000241445700022.
