# Supplementary material for: Molecular characterization of Bathymodiolus mussels and gill symbionts associated with chemosynthetic habitats from the U.S. Atlantic margin
Source: PLoS One. 2019 Mar 14;14(3):e0211616. doi: 10.1371/journal.pone.0211616 (PMC6417655; doi:10.1371/journal.pone.0211616)
Supplement: S9 Table — Reported per primer set (ps1-4) per mussel, after processing by the Mothur pipeline, and after singletons were trimmed in R’s Phyloseq package. (DOCX) [file pone.0211616.s014.docx]

Supplemental Table 9

|  |  |  |  |  |  |  |  |  |  |  |  |  |  |
| --- | --- | --- | --- | --- | --- | --- | --- | --- | --- | --- | --- | --- | --- |
|  |  | MASm22 | MASm30 | MASm5 | MAS538 | MAS562 | MAS100 | MAS109 | MASm34 | MASm36 | MASm45 | Total | % reduction |
| ps1 | total reads | 481647 | 555733 | 550210 | 509925 | 465579 | 515294 | 488730 | 486194 | 451977 | 409421 | 4914710 |  |
|  | after_Mothur | 289136 | 340299 | 297209 | 368641 | 381764 | 421178 | 380944 | 394120 | 330914 | 330007 | 3534212 | 0.281 |
|  | trimmed | 280446 | 330254 | 289834 | 353837 | 366473 | 406418 | 366245 | 379941 | 314567 | 316761 | 3404776 | 0.307 |
| ps2 | total reads | 314157 | 298363 | 391994 | n/a | 319872 | 268116 | 338215 | 354651 | 304200 | 195682 | 2785250 |  |
|  | after_Mothur | 162027 | 149669 | 179991 | n/a | 192181 | 160637 | 182481 | 206598 | 148509 | 106523 | 1488616 | 0.466 |
|  | trimmed | 155294 | 143533 | 173894 | n/a | 180159 | 149825 | 170199 | 194471 | 134507 | 98956 | 1400838 | 0.497 |
| ps3 | total reads | 225891 | 228436 | 308481 | 239702 | 218673 | 154435 | 344383 | 217379 | n/a | 103068 | 2040448 |  |
|  | after_Mothur | 85590 | 84581 | 96163 | 93523 | 89737 | 67176 | 116573 | 91748 | n/a | 40814 | 765905 | 0.625 |
|  | trimmed | 59651 | 63382 | 70105 | 67305 | 67543 | 49351 | 85692 | 68798 | n/a | 30162 | 561989 | 0.725 |
| ps4 | total reads | 434794 | 352530 | 494374 | 2568659 | 1506832 | 473447 | 455565 | 401099 | 538374 | 416170 | 7641844 |  |
|  | after_Mothur | 186605 | 122390 | 189671 | 1138648 | 963564 | 311414 | 260917 | 269834 | 328368 | 231976 | 4003387 | 0.476 |
|  | trimmed | 180559 | 118142 | 183588 | 1097216 | 927837 | 299693 | 251343 | 261573 | 314586 | 224537 | 3859074 | 0.495 |
