# Supplementary material for: Molecular characterization of Bathymodiolus mussels and gill symbionts associated with chemosynthetic habitats from the U.S. Atlantic margin
Source: PLoS One. 2019 Mar 14;14(3):e0211616. doi: 10.1371/journal.pone.0211616 (PMC6417655; doi:10.1371/journal.pone.0211616)
Supplement: S10 Table — This dataset was created in the R package Phyloseq. NCS = Norfolk Canyon Site; BCS = Baltimore Canyon Site. If taxonomies could not be resolved to higher levels, then they were left blank. Only those families with a total of 10 or more reads were listed. (DOCX) [file pone.0211616.s015.docx]

Supplemental Table 10

|  |  |  | NCS | | | BCS | | | | | | |  |  |
| --- | --- | --- | --- | --- | --- | --- | --- | --- | --- | --- | --- | --- | --- | --- |
|  | Phylum | Family | MASm22 | MASm30 | MASm5 | MAS538 | MAS562 | MAS100 | MAS109 | MASm34 | MASm36 | MASm45 | Total | %total |
| ps1 | Proteobacteria | Methylomonaceae | 149321 | 216145 | 127537 | 310123 | 366246 | 404747 | 362958 | 379446 | 310683 | 315567 | 2942773 | 0.8643 |
|  | Campylobacterota | Helicobacteraceae | 128844 | 109367 | 159273 | 43297 | 194 | 1604 | 2884 | 348 | 3677 | 26 | 449514 | 0.1320 |
|  | Bacteria_unc |  | 2012 | 3343 | 1894 | 347 | 14 | 40 | 66 | 12 | 17 | 93 | 7838 | 0.0023 |
|  | Patescibacteria |  | 198 | 1168 | 1071 | 0 | 0 | 2 | 9 | 0 | 0 | 0 | 2448 | 0.0007 |
|  | Proteobacteria |  | 0 | 1 | 0 | 1 | 0 | 1 | 11 | 25 | 0 | 761 | 800 | 0.0002 |
|  | Proteobacteria | Thioglobaceae | 6 | 106 | 5 | 1 | 7 | 0 | 229 | 0 | 0 | 0 | 354 | 0.0001 |
|  | Proteobacteria | Beijerinckiaceae | 13 | 32 | 20 | 12 | 1 | 13 | 32 | 10 | 58 | 11 | 202 | 0.0001 |
|  | Proteobacteria | Rhodobacteraceae | 0 | 0 | 0 | 0 | 0 | 0 | 6 | 28 | 32 | 117 | 183 | 0.0001 |
|  | Proteobacteria |  | 13 | 8 | 8 | 21 | 3 | 4 | 13 | 17 | 16 | 42 | 145 | 0.0000 |
|  | Bacteroidetes | Flavobacteriaceae | 6 | 8 | 4 | 5 | 5 | 0 | 9 | 13 | 9 | 59 | 118 | 0.0000 |
|  | Campylobacterota | Arcobacteraceae | 12 | 30 | 0 | 7 | 0 | 0 | 0 | 1 | 3 | 9 | 62 | 0.0000 |
|  | Bacteroidetes | Cryomorphaceae | 0 | 0 | 0 | 0 | 0 | 0 | 0 | 4 | 8 | 18 | 30 | 0.0000 |
|  | Proteobacteria | Xanthobacteraceae | 1 | 1 | 9 | 1 | 1 | 0 | 2 | 0 | 13 | 0 | 28 | 0.0000 |
|  | Patescibacteria |  | 4 | 18 | 0 | 3 | 0 | 0 | 1 | 1 | 0 | 0 | 27 | 0.0000 |
|  | Proteobacteria |  | 0 | 4 | 0 | 1 | 0 | 0 | 0 | 0 | 4 | 9 | 18 | 0.0000 |
|  | Bacteroidetes | Saprospiraceae | 1 | 2 | 0 | 1 | 0 | 0 | 2 | 0 | 8 | 4 | 18 | 0.0000 |
|  | Actinobacteria | Micrococcaceae | 0 | 0 | 0 | 0 | 0 | 0 | 2 | 7 | 6 | 0 | 15 | 0.0000 |
|  | Campylobacterota |  | 3 | 2 | 4 | 3 | 0 | 0 | 0 | 0 | 0 | 0 | 12 | 0.0000 |
|  | Planctomycetes | Pirellulaceae | 0 | 2 | 0 | 0 | 0 | 0 | 2 | 3 | 2 | 3 | 12 | 0.0000 |
|  | Proteobacteria | Methylophagaceae | 1 | 0 | 0 | 5 | 0 | 0 | 0 | 0 | 0 | 5 | 11 | 0.0000 |
|  | Proteobacteria | Sphingomonadaceae | 0 | 1 | 0 | 0 | 0 | 0 | 1 | 2 | 7 | 0 | 11 | 0.0000 |
|  | Campylobacterota | Sulfurovaceae | 0 | 0 | 0 | 7 | 0 | 0 | 2 | 0 | 0 | 1 | 10 | 0.0000 |
| ps2 | Proteobacteria | Methylomonaceae | 62707 | 69110 | 59182 | n/a | 179378 | 147435 | 163506 | 192822 | 126635 | 98273 | 1099048 | 0.7846 |
|  | Campylobacterota | Helicobacteraceae | 92437 | 73881 | 114299 | n/a | 757 | 2384 | 6609 | 1410 | 7724 | 32 | 299533 | 0.2138 |
|  | Bacteria_unc |  | 35 | 321 | 309 | n/a | 0 | 2 | 2 | 0 | 6 | 3 | 678 | 0.0005 |
|  | Verrucomicrobia | Rubritaleaceae | 0 | 0 | 2 | n/a | 0 | 0 | 1 | 179 | 30 | 333 | 545 | 0.0004 |
|  | Proteobacteria |  | 22 | 60 | 13 | n/a | 0 | 2 | 1 | 2 | 0 | 134 | 234 | 0.0002 |
|  | Campylobacterota |  | 44 | 67 | 51 | n/a | 0 | 0 | 0 | 0 | 0 | 0 | 162 | 0.0001 |
|  | Proteobacteria |  | 25 | 36 | 17 | n/a | 9 | 2 | 45 | 4 | 8 | 13 | 159 | 0.0001 |
|  | Campylobacterota | Arcobacteraceae | 22 | 45 | 18 | n/a | 0 | 0 | 0 | 0 | 7 | 11 | 103 | 0.0001 |
|  | Bacteroidetes |  | 0 | 0 | 0 | n/a | 0 | 0 | 0 | 13 | 9 | 79 | 101 | 0.0001 |
|  | Bacteroidetes | Flavobacteriaceae | 0 | 6 | 2 | n/a | 4 | 0 | 11 | 5 | 26 | 31 | 85 | 0.0001 |
|  | Proteobacteria | Burkholderiaceae | 0 | 1 | 0 | n/a | 2 | 0 | 3 | 3 | 25 | 1 | 35 | 0.0000 |
|  | Planctomycetes | Pirellulaceae | 0 | 0 | 0 | n/a | 5 | 0 | 3 | 11 | 0 | 11 | 30 | 0.0000 |
|  | Proteobacteria | Cellvibrionaceae | 0 | 0 | 0 | n/a | 0 | 0 | 0 | 4 | 6 | 12 | 22 | 0.0000 |
|  | Campylobacterota | Sulfurovaceae | 0 | 4 | 0 | n/a | 0 | 0 | 5 | 0 | 0 | 8 | 17 | 0.0000 |
|  | Bacteroidetes | Weeksellaceae | 1 | 1 | 1 | n/a | 1 | 0 | 1 | 0 | 11 | 0 | 16 | 0.0000 |
|  | Bacteroidetes | Saprospiraceae | 0 | 0 | 0 | n/a | 2 | 0 | 2 | 0 | 8 | 0 | 12 | 0.0000 |
| ps3 | Proteobacteria | Methylomonaceae | 59567 | 62778 | 69998 | 67253 | 67484 | 49341 | 85308 | 68685 | n/a | 29643 | 560057 | 0.9966 |
|  | Proteobacteria | Thioglobaceae | 21 | 421 | 41 | 4 | 25 | 0 | 249 | 1 | n/a | 0 | 762 | 0.0014 |
|  | Chlamydiae |  | 0 | 0 | 0 | 22 | 26 | 5 | 0 | 0 | n/a | 336 | 389 | 0.0007 |
|  | Proteobacteria |  | 37 | 167 | 12 | 7 | 6 | 0 | 115 | 11 | n/a | 12 | 367 | 0.0007 |
|  | Verrucomicrobia | Rubritaleaceae | 1 | 4 | 11 | 0 | 1 | 0 | 0 | 86 | n/a | 92 | 195 | 0.0003 |
|  | Bacteria_unc |  | 0 | 0 | 3 | 1 | 0 | 3 | 10 | 0 | n/a | 30 | 47 | 0.0001 |
|  | Proteobacteria |  | 1 | 2 | 0 | 0 | 0 | 0 | 1 | 5 | n/a | 24 | 33 | 0.0001 |
|  | Proteobacteria | Methylophagaceae | 2 | 0 | 0 | 10 | 1 | 0 | 0 | 0 | n/a | 7 | 20 | 0.0000 |
|  | Proteobacteria | Pseudomonadaceae | 2 | 0 | 5 | 0 | 0 | 0 | 2 | 0 | n/a | 2 | 11 | 0.0000 |
|  | Proteobacteria | Psychromonadaceae | 0 | 2 | 2 | 5 | 0 | 0 | 0 | 0 | n/a | 2 | 11 | 0.0000 |
|  | Proteobacteria | Moraxellaceae | 3 | 0 | 3 | 1 | 0 | 1 | 1 | 0 | n/a | 1 | 10 | 0.0000 |
|  | Proteobacteria | Neisseriaceae | 0 | 0 | 9 | 0 | 0 | 0 | 0 | 1 | n/a | 0 | 10 | 0.0000 |
| ps4 | Proteobacteria | Methylomonaceae | 91780 | 69507 | 84063 | 805493 | 916389 | 289844 | 237571 | 257445 | 288747 | 218158 | 3258997 | 0.8445 |
|  | Campylobacterota | Helicobacteraceae | 88204 | 47896 | 98826 | 289558 | 9951 | 9570 | 12891 | 3427 | 25171 | 152 | 585646 | 0.1518 |
|  | Chlamydiae | Simkaniaceae | 0 | 1 | 0 | 175 | 694 | 43 | 1 | 2 | 0 | 4133 | 5049 | 0.0013 |
|  | Bacteria_unc |  | 465 | 338 | 600 | 1148 | 26 | 44 | 36 | 47 | 46 | 23 | 2773 | 0.0007 |
|  | Proteobacteria | Rhodobacteraceae | 0 | 0 | 0 | 7 | 11 | 3 | 12 | 186 | 135 | 963 | 1317 | 0.0003 |
|  | Proteobacteria |  | 40 | 107 | 13 | 265 | 210 | 72 | 103 | 62 | 119 | 175 | 1166 | 0.0003 |
|  | Proteobacteria | Thioglobaceae | 11 | 105 | 15 | 18 | 266 | 1 | 515 | 1 | 6 | 2 | 940 | 0.0002 |
|  | Bacteroidetes | Flavobacteriaceae | 0 | 3 | 0 | 113 | 60 | 12 | 31 | 107 | 12 | 463 | 801 | 0.0002 |
|  | Proteobacteria |  | 14 | 157 | 8 | 45 | 100 | 59 | 45 | 94 | 12 | 13 | 547 | 0.0001 |
|  | Proteobacteria | Beijerinckiaceae | 12 | 12 | 25 | 46 | 9 | 13 | 26 | 21 | 152 | 39 | 355 | 0.0001 |
|  | Proteobacteria |  | 0 | 0 | 0 | 0 | 1 | 0 | 10 | 36 | 2 | 96 | 145 | 0.0000 |
|  | Campylobacterota | Sulfurovaceae | 0 | 0 | 0 | 108 | 0 | 0 | 5 | 1 | 0 | 5 | 119 | 0.0000 |
|  | Proteobacteria | Methylophagaceae | 0 | 0 | 0 | 72 | 0 | 2 | 1 | 3 | 2 | 22 | 102 | 0.0000 |
|  | Proteobacteria | Psychromonadaceae | 0 | 1 | 0 | 64 | 3 | 0 | 0 | 0 | 2 | 5 | 75 | 0.0000 |
|  | Firmicutes | Streptococcaceae | 3 | 0 | 6 | 8 | 0 | 0 | 5 | 0 | 33 | 14 | 69 | 0.0000 |
|  | Proteobacteria | Cellvibrionaceae | 0 | 0 | 0 | 0 | 0 | 0 | 0 | 5 | 7 | 40 | 52 | 0.0000 |
|  | Proteobacteria | Thiotrichaceae | 2 | 0 | 0 | 34 | 0 | 1 | 3 | 0 | 0 | 2 | 42 | 0.0000 |
|  | Firmicutes | Family_XII | 0 | 0 | 0 | 0 | 0 | 0 | 0 | 16 | 21 | 5 | 42 | 0.0000 |
|  | Proteobacteria | Burkholderiaceae | 0 | 2 | 0 | 1 | 5 | 1 | 13 | 7 | 11 | 0 | 40 | 0.0000 |
|  | Proteobacteria | Rhizobiaceae | 0 | 1 | 0 | 0 | 5 | 0 | 1 | 6 | 8 | 13 | 34 | 0.0000 |
|  | Firmicutes | Aerococcaceae | 0 | 0 | 0 | 0 | 0 | 0 | 3 | 0 | 30 | 0 | 33 | 0.0000 |
|  | Bacteroidetes | Cryomorphaceae | 0 | 0 | 0 | 0 | 0 | 0 | 0 | 3 | 3 | 26 | 32 | 0.0000 |
|  | Firmicutes | Paenibacillaceae | 0 | 0 | 0 | 0 | 10 | 0 | 0 | 4 | 16 | 0 | 30 | 0.0000 |
|  | Proteobacteria |  | 0 | 1 | 0 | 6 | 13 | 5 | 0 | 0 | 1 | 3 | 29 | 0.0000 |
|  | Proteobacteria | Bacteriovoracaceae | 0 | 0 | 0 | 7 | 0 | 0 | 0 | 0 | 3 | 15 | 25 | 0.0000 |
|  | Spirochaetes | Spirochaetaceae | 0 | 2 | 0 | 4 | 0 | 4 | 1 | 13 | 0 | 0 | 24 | 0.0000 |
|  | Firmicutes | Bacillaceae | 1 | 0 | 9 | 0 | 0 | 4 | 2 | 3 | 0 | 4 | 23 | 0.0000 |
|  | Chlamydiae |  | 0 | 0 | 0 | 3 | 0 | 0 | 1 | 0 | 0 | 19 | 23 | 0.0000 |
|  | Firmicutes |  | 0 | 0 | 0 | 0 | 0 | 0 | 6 | 0 | 0 | 17 | 23 | 0.0000 |
|  | Bacteroidetes |  | 0 | 0 | 0 | 0 | 4 | 0 | 6 | 2 | 0 | 11 | 23 | 0.0000 |
|  | Proteobacteria | Moraxellaceae | 2 | 0 | 4 | 0 | 0 | 0 | 0 | 4 | 2 | 7 | 19 | 0.0000 |
|  | Firmicutes | Staphylococcaceae | 0 | 0 | 2 | 0 | 0 | 0 | 3 | 0 | 10 | 4 | 19 | 0.0000 |
|  | Proteobacteria | Hyphomonadaceae | 2 | 2 | 4 | 0 | 0 | 0 | 0 | 7 | 0 | 3 | 18 | 0.0000 |
|  | Firmicutes | Family_XI | 0 | 0 | 0 | 0 | 0 | 0 | 11 | 0 | 0 | 6 | 17 | 0.0000 |
|  | Proteobacteria | Oligoflexaceae | 0 | 0 | 0 | 0 | 15 | 0 | 0 | 0 | 0 | 1 | 16 | 0.0000 |
|  | Bacteroidetes | uncultured | 7 | 0 | 0 | 0 | 0 | 0 | 0 | 7 | 0 | 0 | 14 | 0.0000 |
|  | Proteobacteria | Desulfobacteraceae | 0 | 0 | 0 | 3 | 0 | 2 | 2 | 0 | 6 | 0 | 13 | 0.0000 |
|  | Proteobacteria | Colwelliaceae | 0 | 0 | 0 | 2 | 0 | 0 | 0 | 3 | 5 | 2 | 12 | 0.0000 |
|  | Proteobacteria | Desulfobulbaceae | 0 | 0 | 0 | 0 | 0 | 0 | 3 | 8 | 0 | 1 | 12 | 0.0000 |
|  | Proteobacteria | uncultured | 0 | 0 | 0 | 9 | 0 | 2 | 0 | 1 | 0 | 0 | 12 | 0.0000 |
|  | Proteobacteria | Moritellaceae | 0 | 0 | 0 | 0 | 0 | 0 | 0 | 0 | 9 | 2 | 11 | 0.0000 |
|  | Bacteroidetes | Saprospiraceae | 0 | 0 | 0 | 0 | 11 | 0 | 0 | 0 | 0 | 0 | 11 | 0.0000 |
|  | Firmicutes | Family_XII | 0 | 0 | 9 | 0 | 0 | 0 | 0 | 0 | 0 | 2 | 11 | 0.0000 |
|  | Nitrospirae | Nitrospiraceae | 0 | 0 | 0 | 0 | 0 | 0 | 5 | 0 | 0 | 5 | 10 | 0.0000 |
|  | Bacteroidetes | Porphyromonadaceae | 8 | 0 | 0 | 0 | 2 | 0 | 0 | 0 | 0 | 0 | 10 | 0.0000 |
|  | Proteobacteria | Pseudomonadaceae | 0 | 0 | 0 | 0 | 2 | 0 | 0 | 0 | 0 | 8 | 10 | 0.0000 |
|  | Proteobacteria | Woeseiaceae | 0 | 0 | 0 | 1 | 1 | 0 | 0 | 4 | 0 | 4 | 10 | 0.0000 |
|  | Proteobacteria | Unknown_Family | 2 | 0 | 0 | 0 | 2 | 0 | 4 | 0 | 0 | 2 | 10 | 0.0000 |
