# Supplementary material for: Molecular characterization of Bathymodiolus mussels and gill symbionts associated with chemosynthetic habitats from the U.S. Atlantic margin
Source: PLoS One. 2019 Mar 14;14(3):e0211616. doi: 10.1371/journal.pone.0211616 (PMC6417655; doi:10.1371/journal.pone.0211616)
Supplement: S11 Table — NCS samples are shown in bold. Number of reads are shown per sample per primer set (ps1-4). recon Phy = reconstructed phylotype. C1, C2, and M are the reconstructed phylotypes where the reference sequence KU573880.1, FM994669.1, or AM236329 was used as a scaffold, respectively. See text for details. C1% = # reads from recon Phy C1/ # reads from recon Phy C1 + # reads from recon Phy C2 + # reads from recon Phy M; C2% = # reads from recon Phy C2/ # reads from recon Phy C1 + # reads from recon Phy C2 + # reads from recon Phy M; M% = # reads from recon Phy M/ # reads from recon Phy C1 + # reads from recon Phy C2 + # reads from recon Phy M; Simpson’s Diversity Index (D) = 1- (C1%^2)—(C2%^2)-(M%^2). (DOCX) [file pone.0211616.s016.docx]

Supplemental Table 11

| ps1 | sample | MASM100 | MASM109 | **MASM22** | **MASM30** | MASM34 | MASM36 | MASM45 | **MASM5** | MAS538 | MAS562 |
| --- | --- | --- | --- | --- | --- | --- | --- | --- | --- | --- | --- |
|  | Campylobacterota OTUs | 2111 | 3856 | 180726 | 157567 | 483 | 4844 | 43 | 226662 | 59843 | 255 |
|  | recon Phy M | 471749 | 425706 | 177778 | 253653 | 439973 | 375322 | 365216 | 152913 | 365601 | 425592 |
|  | unmapped reads | 41434 | 59168 | 123143 | 144513 | 45738 | 71811 | 44162 | 170635 | 84481 | 39732 |
|  | total reads | 515294 | 488730 | 481647 | 555733 | 486194 | 451977 | 409421 | 550210 | 509925 | 465579 |
|  | recons Phy C1 | 1086 | 1890 | 26220 | 107971 | 474 | 4988 | 16 | 30022 | 19584 | 187 |
|  | recon Phy C2 | 1057 | 2056 | 148407 | 43460 | 34 | 118 | 29 | 186876 | 39762 | 82 |
|  | recon Phy M | 471749 | 425706 | 177778 | 253653 | 439973 | 375322 | 365216 | 152913 | 365601 | 425592 |
|  | unmapped reads | 41402 | 59078 | 129242 | 150649 | 45713 | 71549 | 44160 | 180399 | 84978 | 39718 |
|  | C1% | 0.002 | 0.004 | 0.074 | 0.267 | 0.001 | 0.013 | 0 | 0.081 | 0.046 | 0 |
|  | C2% | 0.002 | 0.005 | 0.421 | 0.107 | 0 | 0 | 0 | 0.505 | 0.094 | 0 |
|  | M% | 0.995 | 0.991 | 0.504 | 0.626 | 0.999 | 0.987 | 1 | 0.413 | 0.86 | 0.999 |
|  | Simpsons | 0.009 | 0.018 | **0.563** | **0.525** | 0.002 | 0.026 | 0 | **0.567** | 0.249 | 0.001 |
|  | unmapped reads % | 0.08 | 0.121 | **0.268** | **0.271** | 0.094 | 0.158 | 0.108 | **0.328** | 0.167 | 0.085 |
| ps2 | sample | 1 | 2 | 3 | 4 | 5 | 6 | 7 | 8 | 9 | 10 |
|  | Campylobacterota OTUs | 2947 | 8155 | 118462 | 95750 | 1737 | 9275 | 45 | 147370 | n/a | 926 |
|  | recon Phy M | 213637 | 251424 | 90538 | 99935 | 275827 | 188666 | 138214 | 86257 | n/a | 257378 |
|  | unmapped reads | 51532 | 78636 | 105157 | 102678 | 77087 | 106259 | 57423 | 158367 | n/a | 61568 |
|  | total reads | 268116 | 338215 | 314157 | 298363 | 354651 | 304200 | 195682 | 391994 | n/a | 319872 |
|  | recons Phy C1 | 0 | 1 | 0 | 7 | 0 | 4 | 0 | 2 | n/a | 0 |
|  | recon Phy C2 | 1560 | 4196 | 96985 | 32763 | 27 | 278 | 31 | 117917 | n/a | 220 |
|  | recon Phy M | 213637 | 251424 | 90538 | 99935 | 275827 | 188666 | 138214 | 86257 | n/a | 257378 |
|  | unmapped reads | 52919 | 82594 | 126634 | 165658 | 78797 | 115252 | 57437 | 187818 | n/a | 62274 |
|  | C1% | 0 | 0 | 0 | 0 | 0 | 0 | 0 | 0 | n/a | 0 |
|  | C2% | 0.007 | 0.016 | 0.517 | 0.247 | 0 | 0.001 | 0 | 0.578 | n/a | 0.001 |
|  | M% | 0.993 | 0.984 | 0.483 | 0.753 | 1 | 0.999 | 1 | 0.422 | n/a | 0.999 |
|  | Simpsons | 0.014 | 0.032 | **0.499** | **0.372** | 0 | 0.003 | 0 | **0.488** | n/a | 0.002 |
|  | unmapped reads % | 0.197 | 0.244 | **0.403** | **0.555** | 0.222 | 0.379 | 0.294 | **0.479** | n/a | 0.195 |
| ps3 | sample | 1 | 2 | 3 | 4 | 5 | 6 | 7 | 8 | 9 | 10 |
|  | Campylobacterota OTUs | 1 | 1 | 7 | 6 | 0 | n/a | 0 | 11 | 16 | 2 |
|  | recon Phy M | 81988 | 142891 | 102864 | 104547 | 112995 | n/a | 49228 | 119316 | 116192 | 112912 |
|  | unmapped reads | 72446 | 201491 | 123020 | 123883 | 104384 | n/a | 53840 | 189154 | 123494 | 105759 |
|  | total reads | 154435 | 344383 | 225891 | 228436 | 217379 | n/a | 103068 | 308481 | 239702 | 218673 |
|  | recons Phy C1 | 0 | 0 | 1 | 2 | 0 | n/a | 0 | 1 | 9 | 0 |
|  | recon Phy C2 | 0 | 1 | 211 | 17 | 0 | n/a | 0 | 271 | 30 | 0 |
|  | recon Phy M | 81988 | 142891 | 102864 | 104547 | 112995 | n/a | 49228 | 119316 | 116192 | 112912 |
|  | unmapped reads | 72447 | 201491 | 122815 | 123870 | 104384 | n/a | 53840 | 188893 | 123471 | 105761 |
|  | C1% | 0 | 0 | 0 | 0 | 0 | n/a | 0 | 0 | 0 | 0 |
|  | C2% | 0 | 0 | 0.002 | 0 | 0 | n/a | 0 | 0.002 | 0 | 0 |
|  | M% | 1 | 1 | 0.998 | 1 | 1 | n/a | 1 | 0.998 | 1 | 1 |
|  | Simpsons | 0 | 0 | **0.004** | **0** | 0 | n/a | 0 | **0.005** | 0.001 | 0 |
|  | unmapped reads % | 0.469 | 0.585 | **0.544** | **0.542** | 0.48 | n/a | 0.522 | **0.612** | 0.515 | 0.484 |
| ps4 | sample | 1 | 2 | 3 | 4 | 5 | 6 | 7 | 8 | 9 | 10 |
|  | Campylobacterota OTUs | 21379 | 26941 | 144130 | 89744 | 6693 | 40843 | 311 | 158300 | 555860 | 19507 |
|  | recon Phy M | 348319 | 285725 | 112946 | 91718 | 308841 | 350310 | 259094 | 105102 | 978760 | 1107835 |
|  | unmapped reads | 103749 | 142899 | 177718 | 171068 | 85565 | 147221 | 156765 | 230972 | 1034039 | 379490 |
|  | total reads | 473447 | 455565 | 434794 | 352530 | 401099 | 538374 | 416170 | 494374 | 2568659 | 1506832 |
|  | recons Phy C1 | 8828 | 11216 | 28250 | 52167 | 4347 | 31071 | 122 | 32641 | 181928 | 11024 |
|  | recon Phy C2 | 4799 | 7048 | 99764 | 24687 | 148 | 1019 | 106 | 111239 | 261349 | 2131 |
|  | recon Phy M | 348319 | 285725 | 112946 | 91718 | 308841 | 350310 | 259094 | 105102 | 978760 | 1107835 |
|  | unmapped reads | 111501 | 151576 | 193834 | 183958 | 87763 | 155974 | 156848 | 245392 | 1146622 | 385842 |
|  | C1% | 0.024 | 0.037 | 0.117 | 0.309 | 0.014 | 0.081 | 0 | 0.131 | 0.128 | 0.01 |
|  | C2% | 0.013 | 0.023 | 0.414 | 0.146 | 0 | 0.003 | 0 | 0.447 | 0.184 | 0.002 |
|  | M% | 0.962 | 0.94 | 0.469 | 0.544 | 0.986 | 0.916 | 0.999 | 0.422 | 0.688 | 0.988 |
|  | Simpsons | 0.073 | 0.115 | **0.595** | **0.587** | 0.028 | 0.154 | 0.002 | **0.605** | 0.476 | 0.023 |
|  | unmapped reads % | 0.236 | 0.333 | **0.446** | **0.522** | 0.219 | 0.29 | 0.377 | **0.496** | 0.446 | 0.256 |
