# Supplementary material for: Molecular characterization of Bathymodiolus mussels and gill symbionts associated with chemosynthetic habitats from the U.S. Atlantic margin
Source: PLoS One. 2019 Mar 14;14(3):e0211616. doi: 10.1371/journal.pone.0211616 (PMC6417655; doi:10.1371/journal.pone.0211616)
Supplement: S12 Table — Nucleotide positions range from 1 to 1184, referring to the length of the 16S consensus sequence used as a reference. Mismatch percentage indicates how many nucleotide differences occurred at that nucleotide position. Coverage = the number of nucleotides from our reads that mapped to that nucleotide position. (DOCX) [file pone.0211616.s017.docx]

Supplemental Table 12

| Position | Mismatch (%) | Coverage |
| --- | --- | --- |
| 1 | 0.00000 | 87303 |
| 2 | 0.00000 | 87306 |
| 3 | 0.00616 | 87326 |
| 4 | 0.00226 | 87340 |
| 5 | 0.00690 | 87344 |
| 6 | 0.00589 | 87347 |
| 7 | 0.01204 | 87356 |
| 8 | 0.00614 | 87360 |
| 9 | 0.00526 | 87363 |
| 10 | 0.00614 | 87363 |
| 11 | 0.00614 | 87364 |
| 12 | 0.00852 | 87365 |
| 13 | 0.00426 | 87365 |
| 14 | 0.00426 | 87365 |
| 15 | 0.00526 | 87365 |
| 16 | 0.00876 | 87366 |
| 17 | 0.00776 | 87366 |
| 18 | 0.01939 | 87366 |
| 19 | 0.01338 | 87366 |
| 20 | 0.00838 | 87366 |
| 21 | 0.00488 | 87366 |
| 22 | 0.00363 | 87366 |
| 23 | 0.00388 | 87368 |
| 24 | 0.00775 | 87368 |
| 25 | 0.00475 | 87368 |
| 26 | 0.00562 | 87369 |
| 27 | 0.00500 | 87370 |
| 28 | 0.01611 | 87370 |
| 29 | 0.00699 | 87370 |
| 30 | 0.00687 | 87372 |
| 31 | 0.00974 | 87372 |
| 32 | 0.00437 | 87373 |
| 33 | 0.00350 | 87373 |
| 34 | 0.00425 | 87376 |
| 35 | 0.00462 | 87377 |
| 36 | 0.00537 | 87378 |
| 37 | 0.01111 | 87378 |
| 38 | 0.00487 | 87378 |
| 39 | 0.00362 | 87378 |
| 40 | 0.00400 | 87378 |
| 41 | 0.00262 | 87380 |
| 42 | 0.01436 | 87380 |
| 43 | 0.00437 | 87381 |
| 44 | 0.00649 | 87381 |
| 45 | 0.01473 | 87381 |
| 46 | 0.01485 | 87384 |
| 47 | 0.00974 | 87384 |
| 48 | 0.00262 | 87384 |
| 49 | 0.00412 | 87384 |
| 50 | 0.00449 | 87385 |
| 51 | 0.00474 | 87385 |
| 52 | 0.00874 | 87385 |
| 53 | 0.00374 | 87385 |
| 54 | 0.00611 | 87385 |
| 55 | 0.00462 | 87385 |
| 56 | 0.00923 | 87385 |
| 57 | 0.00811 | 87385 |
| 58 | 0.00474 | 87385 |
| 59 | 0.00561 | 87385 |
| 60 | 0.00200 | 87385 |
| 61 | 0.00424 | 87385 |
| 62 | 0.00424 | 87385 |
| 63 | 0.00249 | 87385 |
| 64 | 0.00698 | 87385 |
| 65 | 0.01422 | 87385 |
| 66 | 0.01696 | 87385 |
| 67 | 0.00636 | 87385 |
| 68 | 0.00736 | 87385 |
| 69 | 0.00437 | 87385 |
| 70 | 0.00362 | 87385 |
| 71 | 0.00711 | 87385 |
| 72 | 0.00175 | 87385 |
| 73 | 0.00935 | 87385 |
| 74 | 0.00412 | 87385 |
| 75 | 0.00561 | 87385 |
| 76 | 0.00973 | 87385 |
| 77 | 0.00686 | 87385 |
| 78 | 0.00686 | 87385 |
| 79 | 0.01234 | 87385 |
| 80 | 0.00424 | 87385 |
| 81 | 0.00312 | 87385 |
| 82 | 0.00362 | 87385 |
| 83 | 0.00561 | 87385 |
| 84 | 0.00474 | 87385 |
| 85 | 0.01072 | 87385 |
| 86 | 0.00673 | 87385 |
| 87 | 0.00536 | 87385 |
| 88 | 0.00237 | 87385 |
| 89 | 0.00187 | 87385 |
| 90 | 0.00212 | 87385 |
| 91 | 0.00411 | 87385 |
| 92 | 0.00461 | 87385 |
| 93 | 0.02169 | 87385 |
| 94 | 0.00399 | 87385 |
| 95 | 0.00461 | 87385 |
| 96 | 0.00075 | 87385 |
| 97 | 0.00386 | 87385 |
| 98 | 0.00299 | 87385 |
| 99 | 0.00711 | 87385 |
| 100 | 0.00075 | 87385 |
| 101 | 0.00573 | 87385 |
| 102 | 0.00424 | 87385 |
| 103 | 0.00249 | 87385 |
| 104 | 0.00287 | 87385 |
| 105 | 0.02381 | 87385 |
| 106 | 0.00349 | 87385 |
| 107 | 0.01384 | 87385 |
| 108 | 0.00350 | 87385 |
| 109 | 0.00550 | 87385 |
| 110 | 0.00187 | 87385 |
| 111 | 0.00325 | 87385 |
| 112 | 0.00225 | 87385 |
| 113 | 0.00848 | 87385 |
| 114 | 0.00112 | 87385 |
| 115 | 0.00598 | 87385 |
| 116 | 0.01371 | 87385 |
| 117 | 0.00362 | 87385 |
| 118 | 0.00598 | 87385 |
| 119 | 0.00399 | 87385 |
| 120 | 0.01137 | 87385 |
| 121 | 0.00375 | 87385 |
| 122 | 0.00162 | 87385 |
| 123 | 0.00262 | 87385 |
| 124 | 0.00399 | 87385 |
| 125 | 0.00549 | 87385 |
| 126 | 0.00449 | 87385 |
| 127 | 0.00125 | 87385 |
| 128 | 0.00324 | 87385 |
| 129 | 0.00212 | 87385 |
| 130 | 0.00573 | 87385 |
| 131 | 0.00287 | 87385 |
| 132 | 0.00249 | 87385 |
| 133 | 0.00075 | 87385 |
| 134 | 0.00212 | 87385 |
| 135 | 0.00274 | 87385 |
| 136 | 0.00960 | 87385 |
| 137 | 0.00175 | 87385 |
| 138 | 0.00411 | 87385 |
| 139 | 0.00112 | 87385 |
| 140 | 0.00411 | 87385 |
| 141 | 0.00598 | 87385 |
| 142 | 0.00087 | 87385 |
| 143 | 0.00748 | 87385 |
| 144 | 0.00187 | 87385 |
| 145 | 0.00349 | 87385 |
| 146 | 0.00237 | 87385 |
| 147 | 0.00324 | 87385 |
| 148 | 0.00362 | 87385 |
| 149 | 0.00150 | 87385 |
| 150 | 0.00474 | 87385 |
| 151 | 0.00212 | 87385 |
| 152 | 0.00873 | 87385 |
| 153 | 0.00212 | 87385 |
| 154 | 0.00412 | 87385 |
| 155 | 0.00412 | 87385 |
| 156 | 0.00212 | 87385 |
| 157 | 0.00125 | 87385 |
| 158 | 0.00187 | 87385 |
| 159 | 0.00586 | 87385 |
| 160 | 0.03032 | 87385 |
| 161 | 0.02371 | 87385 |
| 162 | 0.00125 | 87385 |
| 163 | 0.00237 | 87385 |
| 164 | 0.00548 | 87385 |
| 165 | 0.00561 | 87385 |
| 166 | 0.00250 | 87385 |
| 167 | 0.00150 | 87385 |
| 168 | 0.00212 | 87385 |
| 169 | 0.01010 | 87385 |
| 170 | 0.01583 | 87385 |
| 171 | 0.00199 | 87385 |
| 172 | 0.00486 | 87385 |
| 173 | 0.00561 | 87385 |
| 174 | 0.00237 | 87385 |
| 175 | 0.00810 | 87385 |
| 176 | 0.01147 | 87385 |
| 177 | 0.00573 | 87385 |
| 178 | 0.00112 | 87385 |
| 179 | 0.00536 | 87385 |
| 180 | 0.00324 | 87385 |
| 181 | 0.00199 | 87385 |
| 182 | 0.01957 | 87385 |
| 183 | 0.00723 | 87385 |
| 184 | 0.00511 | 87385 |
| 185 | 0.00075 | 87385 |
| 186 | 0.00586 | 87385 |
| 187 | 0.00948 | 87385 |
| 188 | 0.01459 | 87385 |
| 189 | 0.00199 | 87385 |
| 190 | 0.00798 | 87385 |
| 191 | 0.01458 | 87385 |
| 192 | 0.01309 | 87385 |
| 193 | 0.00237 | 87385 |
| 194 | 0.00262 | 87385 |
| 195 | 0.00424 | 87385 |
| 196 | 0.00162 | 87385 |
| 197 | 0.00125 | 87385 |
| 198 | 0.00100 | 87385 |
| 199 | 0.00362 | 87385 |
| 200 | 0.00262 | 87385 |
| 201 | 0.00424 | 87385 |
| 202 | 0.00187 | 87385 |
| 203 | 0.01060 | 87385 |
| 204 | 0.00274 | 87385 |
| 205 | 0.00349 | 87385 |
| 206 | 0.00112 | 87385 |
| 207 | 0.00474 | 87385 |
| 208 | 0.00486 | 87385 |
| 209 | 0.00262 | 87385 |
| 210 | 0.00399 | 87385 |
| 211 | 0.00212 | 87385 |
| 212 | 0.00137 | 87385 |
| 213 | 0.00486 | 87385 |
| 214 | 0.00536 | 87385 |
| 215 | 0.00137 | 87385 |
| 216 | 0.00374 | 87385 |
| 217 | 0.00349 | 87385 |
| 218 | 0.00137 | 87385 |
| 219 | 0.00212 | 87385 |
| 220 | 0.00562 | 87385 |
| 221 | 0.00511 | 87385 |
| 222 | 0.00561 | 87385 |
| 223 | 0.00137 | 87385 |
| 224 | 0.00511 | 87385 |
| 225 | 0.00436 | 87385 |
| 226 | 0.00150 | 87385 |
| 227 | 0.00624 | 87385 |
| 228 | 0.01160 | 87385 |
| 229 | 0.00125 | 87385 |
| 230 | 0.00349 | 87385 |
| 231 | 0.00337 | 87385 |
| 232 | 0.00986 | 87385 |
| 233 | 0.00299 | 87385 |
| 234 | 0.00287 | 87385 |
| 235 | 0.00212 | 87385 |
| 236 | 0.00324 | 87385 |
| 237 | 0.00412 | 87385 |
| 238 | 0.00436 | 87385 |
| 239 | 0.00062 | 87385 |
| 240 | 0.00125 | 87385 |
| 241 | 0.00287 | 87385 |
| 242 | 0.00125 | 87385 |
| 243 | 0.01385 | 87385 |
| 244 | 0.00274 | 87385 |
| 245 | 0.00187 | 87385 |
| 246 | 0.00349 | 87385 |
| 247 | 0.00736 | 87385 |
| 248 | 0.00125 | 87385 |
| 249 | 0.00462 | 87385 |
| 250 | 0.00411 | 87385 |
| 251 | 0.00262 | 87385 |
| 252 | 0.00224 | 87385 |
| 253 | 0.00424 | 87385 |
| 254 | 0.00337 | 87385 |
| 255 | 0.00861 | 87385 |
| 256 | 0.00262 | 87385 |
| 257 | 0.00312 | 87385 |
| 258 | 0.00461 | 87385 |
| 259 | 0.00175 | 87385 |
| 260 | 0.00761 | 87385 |
| 261 | 0.00224 | 87385 |
| 262 | 0.00961 | 87385 |
| 263 | 0.00087 | 87385 |
| 264 | 0.00611 | 87385 |
| 265 | 0.00224 | 87385 |
| 266 | 0.00249 | 87385 |
| 267 | 0.00386 | 87385 |
| 268 | 0.00362 | 87385 |
| 269 | 0.00237 | 87385 |
| 270 | 0.00574 | 87385 |
| 271 | 0.01047 | 87385 |
| 272 | 0.00224 | 87385 |
| 273 | 0.00424 | 87385 |
| 274 | 0.00350 | 87385 |
| 275 | 0.02636 | 87385 |
| 276 | 0.08171 | 87385 |
| 277 | 0.04345 | 87385 |
| 278 | 0.14992 | 87385 |
| 279 | 0.04086 | 87385 |
| 280 | 0.05026 | 87385 |
| 281 | 0.15551 | 87385 |
| 282 | 0.01358 | 87385 |
| 283 | 0.00716 | 87385 |
| 284 | 0.02049 | 87385 |
| 285 | 0.05216 | 87385 |
| 286 | 0.07099 | 87385 |
| 287 | 0.16830 | 87385 |
| 288 | 0.02035 | 87385 |
| 289 | 0.14136 | 87385 |
| 290 | 0.00920 | 87385 |
| 291 | 0.03623 | 87385 |
| 292 | 0.20476 | 87385 |
| 293 | 0.02212 | 87385 |
| 294 | 0.07729 | 87385 |
| 295 | 0.00677 | 87385 |
| 296 | 0.00928 | 87385 |
| 297 | 0.22304 | 87385 |
| 298 | 0.10895 | 87385 |
| 299 | 0.15029 | 87385 |
| 300 | 0.03404 | 87385 |
| 301 | 0.00902 | 87385 |
| 302 | 0.02903 | 87385 |
| 303 | 0.05308 | 87385 |
| 304 | 0.10064 | 87385 |
| 305 | 0.10327 | 87385 |
| 306 | 0.11075 | 87385 |
| 307 | 0.08839 | 87385 |
| 308 | 0.07607 | 87385 |
| 309 | 0.04961 | 87385 |
| 310 | 0.00463 | 87385 |
| 311 | 0.03228 | 87385 |
| 312 | 0.00325 | 87385 |
| 313 | 0.04438 | 87385 |
| 314 | 0.00425 | 87385 |
| 315 | 0.00700 | 87385 |
| 316 | 0.10071 | 87385 |
| 317 | 0.01336 | 87385 |
| 318 | 0.01011 | 87385 |
| 319 | 0.08359 | 87385 |
| 320 | 0.01899 | 87386 |
| 321 | 0.00400 | 87388 |
| 322 | 0.03847 | 87478 |
| 323 | 0.00425 | 187268 |
| 324 | 0.01201 | 187298 |
| 325 | 0.00425 | 187304 |
| 326 | 0.00763 | 187311 |
| 327 | 0.02133 | 187312 |
| 328 | 0.04004 | 187314 |
| 329 | 0.09131 | 187399 |
| 330 | 0.02557 | 187416 |
| 331 | 0.07265 | 187430 |
| 332 | 0.10357 | 187431 |
| 333 | 0.01211 | 187435 |
| 334 | 0.01510 | 187437 |
| 335 | 0.03342 | 187437 |
| 336 | 0.07469 | 187439 |
| 337 | 0.05004 | 187445 |
| 338 | 0.01485 | 187451 |
| 339 | 0.06470 | 187507 |
| 340 | 0.00274 | 187512 |
| 341 | 0.02469 | 187516 |
| 342 | 0.00723 | 187516 |
| 343 | 0.04061 | 187516 |
| 344 | 0.01059 | 187516 |
| 345 | 0.03959 | 187517 |
| 346 | 0.01171 | 187517 |
| 347 | 0.01133 | 187518 |
| 348 | 0.00585 | 187518 |
| 349 | 0.07232 | 187519 |
| 350 | 0.05415 | 187522 |
| 351 | 0.06950 | 187592 |
| 352 | 0.00785 | 187598 |
| 353 | 0.01869 | 187598 |
| 354 | 0.02590 | 187598 |
| 355 | 0.04060 | 187598 |
| 356 | 0.03373 | 187600 |
| 357 | 0.02115 | 187600 |
| 358 | 0.00598 | 187600 |
| 359 | 0.06599 | 187600 |
| 360 | 0.01120 | 187603 |
| 361 | 0.00921 | 187603 |
| 362 | 0.02115 | 187604 |
| 363 | 0.01843 | 187604 |
| 364 | 0.05267 | 187605 |
| 365 | 0.03673 | 187605 |
| 366 | 0.00461 | 187605 |
| 367 | 0.03375 | 187605 |
| 368 | 0.00638 | 187605 |
| 369 | 0.05432 | 187606 |
| 370 | 0.00601 | 187607 |
| 371 | 0.03630 | 187607 |
| 372 | 0.00250 | 187607 |
| 373 | 0.08343 | 187607 |
| 374 | 0.00675 | 187607 |
| 375 | 0.01505 | 187607 |
| 376 | 0.02924 | 187607 |
| 377 | 0.01792 | 187607 |
| 378 | 0.02923 | 187607 |
| 379 | 0.01790 | 187607 |
| 380 | 0.00758 | 187607 |
| 381 | 0.01429 | 187607 |
| 382 | 0.02237 | 187614 |
| 383 | 0.03529 | 397717 |
| 384 | 0.02932 | 397720 |
| 385 | 0.00870 | 397725 |
| 386 | 0.01466 | 397728 |
| 387 | 0.01480 | 397729 |
| 388 | 0.01342 | 397729 |
| 389 | 0.01119 | 397839 |
| 390 | 0.04896 | 397840 |
| 391 | 0.02187 | 397840 |
| 392 | 0.00646 | 397845 |
| 393 | 0.02671 | 397849 |
| 394 | 0.02950 | 397849 |
| 395 | 0.05078 | 397852 |
| 396 | 0.01468 | 397852 |
| 397 | 0.00608 | 397856 |
| 398 | 0.02522 | 397857 |
| 399 | 0.01379 | 397857 |
| 400 | 0.02248 | 397857 |
| 401 | 0.05106 | 397861 |
| 402 | 0.08745 | 397861 |
| 403 | 0.04560 | 397862 |
| 404 | 0.04135 | 397862 |
| 405 | 0.01143 | 397862 |
| 406 | 0.02372 | 397864 |
| 407 | 0.02621 | 397864 |
| 408 | 0.01640 | 397864 |
| 409 | 0.00584 | 397864 |
| 410 | 0.03281 | 397863 |
| 411 | 0.01007 | 397863 |
| 412 | 0.03168 | 397864 |
| 413 | 0.02859 | 397864 |
| 414 | 0.01181 | 397865 |
| 415 | 0.01743 | 397866 |
| 416 | 0.06397 | 397876 |
| 417 | 0.07901 | 397877 |
| 418 | 0.02923 | 397880 |
| 419 | 0.03218 | 397880 |
| 420 | 0.02722 | 397881 |
| 421 | 0.00596 | 397881 |
| 422 | 0.01489 | 397881 |
| 423 | 0.03017 | 397878 |
| 424 | 0.02049 | 397878 |
| 425 | 0.00807 | 397878 |
| 426 | 0.01280 | 397887 |
| 427 | 0.04619 | 397885 |
| 428 | 0.09358 | 397870 |
| 429 | 0.01131 | 397861 |
| 430 | 0.02847 | 397852 |
| 431 | 0.02387 | 397852 |
| 432 | 0.03557 | 397850 |
| 433 | 0.16084 | 397835 |
| 434 | 0.03419 | 397830 |
| 435 | 0.02760 | 397821 |
| 436 | 0.07362 | 397808 |
| 437 | 0.00336 | 397807 |
| 438 | 0.06217 | 397776 |
| 439 | 0.02215 | 397772 |
| 440 | 0.03425 | 397749 |
| 441 | 0.03675 | 397733 |
| 442 | 0.04499 | 397732 |
| 443 | 0.07570 | 397730 |
| 444 | 0.00461 | 397717 |
| 445 | 0.04100 | 397666 |
| 446 | 0.01708 | 397662 |
| 447 | 0.02845 | 397662 |
| 448 | 0.03199 | 397656 |
| 449 | 0.04077 | 397656 |
| 450 | 0.04186 | 397657 |
| 451 | 0.02655 | 397538 |
| 452 | 0.27348 | 397535 |
| 453 | 0.03106 | 397528 |
| 454 | 0.02742 | 397500 |
| 455 | 0.02720 | 397468 |
| 456 | 0.02981 | 397454 |
| 457 | 0.03406 | 397446 |
| 458 | 0.02230 | 397436 |
| 459 | 0.02644 | 397435 |
| 460 | 0.01516 | 397434 |
| 461 | 0.01870 | 397434 |
| 462 | 0.03652 | 397426 |
| 463 | 0.01670 | 397426 |
| 464 | 0.01952 | 397341 |
| 465 | 0.02912 | 397328 |
| 466 | 0.03556 | 397220 |
| 467 | 0.02183 | 397220 |
| 468 | 0.00744 | 397209 |
| 469 | 0.00860 | 397139 |
| 470 | 0.01962 | 397115 |
| 471 | 0.02333 | 397079 |
| 472 | 0.00877 | 397039 |
| 473 | 0.01443 | 396997 |
| 474 | 0.00026 | 396928 |
| 475 | 0.00013 | 396916 |
| 476 | 0.00013 | 310657 |
| 477 | 0.00000 | 310523 |
| 478 | 0.01695 | 310519 |
| 479 | 0.00000 | 310511 |
| 480 | 0.01667 | 310509 |
| 481 | 0.01695 | 310509 |
| 482 | 0.00000 | 310509 |
| 483 | 0.00000 | 310509 |
| 484 | 0.00000 | 310509 |
| 485 | 0.00000 | 310509 |
| 486 | 0.01695 | 310509 |
| 487 | 0.00000 | 310509 |
| 488 | 0.01695 | 310509 |
| 489 | 0.00000 | 310509 |
| 490 | 0.00000 | 310509 |
| 491 | 0.01695 | 310509 |
| 492 | 0.01695 | 310509 |
| 493 | 0.00000 | 310509 |
| 494 | 0.00000 | 310509 |
| 495 | 0.00000 | 310509 |
| 496 | 0.00000 | 310509 |
| 497 | 0.00000 | 310509 |
| 498 | 0.01695 | 310509 |
| 499 | 0.00000 | 310509 |
| 500 | 0.00000 | 310509 |
| 501 | 0.01695 | 310509 |
| 502 | 0.00000 | 310509 |
| 503 | 0.01695 | 310509 |
| 504 | 0.00000 | 310509 |
| 505 | 0.00000 | 310509 |
| 506 | 0.01695 | 310509 |
| 507 | 0.01695 | 310509 |
| 508 | 0.00000 | 310509 |
| 509 | 0.00000 | 310509 |
| 510 | 0.00000 | 310509 |
| 511 | 0.00000 | 310509 |
| 512 | 0.01695 | 310509 |
| 513 | 0.00000 | 310509 |
| 514 | 0.00000 | 310509 |
| 515 | 0.01695 | 310509 |
| 516 | 0.01695 | 310509 |
| 517 | 0.03390 | 310509 |
| 518 | 0.00000 | 310509 |
| 519 | 0.00000 | 310509 |
| 520 | 0.00000 | 310509 |
| 521 | 0.00000 | 310509 |
| 522 | 0.00000 | 310509 |
| 523 | 0.00000 | 310509 |
| 524 | 0.00000 | 310509 |
| 525 | 0.00000 | 310509 |
| 526 | 0.00000 | 310509 |
| 527 | 0.01695 | 310509 |
| 528 | 0.00000 | 310509 |
| 529 | 0.00000 | 310509 |
| 530 | 0.00000 | 310509 |
| 531 | 0.01695 | 310509 |
| 532 | 0.00000 | 310509 |
| 533 | 0.00000 | 310509 |
| 534 | 0.00000 | 310509 |
| 535 | 0.00000 | 310509 |
| 536 | 0.00000 | 310509 |
| 537 | 0.00000 | 310509 |
| 538 | 0.00000 | 310509 |
| 539 | 0.00000 | 310509 |
| 540 | 0.00000 | 310509 |
| 541 | 0.01695 | 310509 |
| 542 | 0.01695 | 310509 |
| 543 | 0.03390 | 310509 |
| 544 | 0.00000 | 310509 |
| 545 | 0.00000 | 310509 |
| 546 | 0.00000 | 310509 |
| 547 | 0.00000 | 310509 |
| 548 | 0.00000 | 310509 |
| 549 | 0.01695 | 310509 |
| 550 | 0.00000 | 310509 |
| 551 | 0.00000 | 310509 |
| 552 | 0.00000 | 310509 |
| 553 | 0.03390 | 310509 |
| 554 | 0.00000 | 310509 |
| 555 | 0.01695 | 310509 |
| 556 | 0.00000 | 310509 |
| 557 | 0.01695 | 310509 |
| 558 | 0.00000 | 310509 |
| 559 | 0.00000 | 310509 |
| 560 | 0.00000 | 310509 |
| 561 | 0.00000 | 310509 |
| 562 | 0.01695 | 310509 |
| 563 | 0.00000 | 310509 |
| 564 | 0.00000 | 310509 |
| 565 | 0.01695 | 310509 |
| 566 | 0.00000 | 310509 |
| 567 | 0.00000 | 310509 |
| 568 | 0.01695 | 310509 |
| 569 | 0.00000 | 310509 |
| 570 | 0.00000 | 310509 |
| 571 | 0.03390 | 310509 |
| 572 | 0.01695 | 310509 |
| 573 | 0.00000 | 310509 |
| 574 | 0.01695 | 310509 |
| 575 | 0.00000 | 310509 |
| 576 | 0.00000 | 310509 |
| 577 | 0.00000 | 310509 |
| 578 | 0.00000 | 310509 |
| 579 | 0.00000 | 310509 |
| 580 | 0.00000 | 310509 |
| 581 | 0.00000 | 310509 |
| 582 | 0.00000 | 310509 |
| 583 | 0.00000 | 310509 |
| 584 | 0.01695 | 310509 |
| 585 | 0.00000 | 310509 |
| 586 | 0.00000 | 310509 |
| 587 | 0.00000 | 310509 |
| 588 | 0.00000 | 310509 |
| 589 | 0.00000 | 310509 |
| 590 | 0.00000 | 310509 |
| 591 | 0.00000 | 310509 |
| 592 | 0.01695 | 310509 |
| 593 | 0.00000 | 310509 |
| 594 | 0.00000 | 310509 |
| 595 | 0.01695 | 310509 |
| 596 | 0.00000 | 310509 |
| 597 | 0.00000 | 310509 |
| 598 | 0.00000 | 310509 |
| 599 | 0.01695 | 310509 |
| 600 | 0.01695 | 310509 |
| 601 | 0.03390 | 310509 |
| 602 | 0.01695 | 310509 |
| 603 | 0.00000 | 310509 |
| 604 | 0.00000 | 310509 |
| 605 | 0.00000 | 310509 |
| 606 | 0.01695 | 310509 |
| 607 | 0.03390 | 310509 |
| 608 | 0.00000 | 310509 |
| 609 | 0.00000 | 310509 |
| 610 | 0.00000 | 310509 |
| 611 | 0.00000 | 310509 |
| 612 | 0.00000 | 310509 |
| 613 | 0.00000 | 310509 |
| 614 | 0.05085 | 310509 |
| 615 | 0.03390 | 310509 |
| 616 | 0.00000 | 310509 |
| 617 | 0.00000 | 310509 |
| 618 | 0.00000 | 310509 |
| 619 | 0.00000 | 310509 |
| 620 | 0.03390 | 310509 |
| 621 | 0.03390 | 310509 |
| 622 | 0.01695 | 310509 |
| 623 | 0.00000 | 310509 |
| 624 | 0.00000 | 310509 |
| 625 | 0.00000 | 310509 |
| 626 | 0.01695 | 310509 |
| 627 | 0.00000 | 310509 |
| 628 | 0.00000 | 310509 |
| 629 | 0.00000 | 310509 |
| 630 | 0.01695 | 310509 |
| 631 | 0.00000 | 310509 |
| 632 | 0.01695 | 310509 |
| 633 | 0.01695 | 310509 |
| 634 | 0.00000 | 310509 |
| 635 | 0.00000 | 310509 |
| 636 | 0.01695 | 310509 |
| 637 | 0.01695 | 310509 |
| 638 | 0.00000 | 310509 |
| 639 | 0.00000 | 310509 |
| 640 | 0.00000 | 310509 |
| 641 | 0.00000 | 310509 |
| 642 | 0.00000 | 310509 |
| 643 | 0.00000 | 310509 |
| 644 | 0.00000 | 310509 |
| 645 | 0.00000 | 310509 |
| 646 | 0.01695 | 310509 |
| 647 | 0.00000 | 310509 |
| 648 | 0.00000 | 310509 |
| 649 | 0.00000 | 310509 |
| 650 | 0.00000 | 310509 |
| 651 | 0.00000 | 310509 |
| 652 | 0.00000 | 310509 |
| 653 | 0.03390 | 310509 |
| 654 | 0.00000 | 310509 |
| 655 | 0.01695 | 310509 |
| 656 | 0.01695 | 310509 |
| 657 | 0.01695 | 310509 |
| 658 | 0.01695 | 310509 |
| 659 | 0.00000 | 310509 |
| 660 | 0.00000 | 310509 |
| 661 | 0.00000 | 310509 |
| 662 | 0.01695 | 310509 |
| 663 | 0.01695 | 310509 |
| 664 | 0.00000 | 310509 |
| 665 | 0.01695 | 310509 |
| 666 | 0.01695 | 310509 |
| 667 | 0.05085 | 310509 |
| 668 | 0.01695 | 310509 |
| 669 | 0.05085 | 310509 |
| 670 | 0.08475 | 310509 |
| 671 | 0.05085 | 310509 |
| 672 | 0.03390 | 310509 |
| 673 | 0.00000 | 310509 |
| 674 | 0.00000 | 310509 |
| 675 | 0.01695 | 310509 |
| 676 | 0.03390 | 310509 |
| 677 | 0.00000 | 310509 |
| 678 | 0.01724 | 310509 |
| 679 | 0.03448 | 310509 |
| 680 | 0.03448 | 310509 |
| 681 | 0.03448 | 310509 |
| 682 | 0.05172 | 310509 |
| 683 | 0.01724 | 310509 |
| 684 | 0.03390 | 310509 |
| 685 | 0.03448 | 310509 |
| 686 | 0.05085 | 310509 |
| 687 | 0.00000 | 310509 |
| 688 | 0.05172 | 310509 |
| 689 | 0.03390 | 310509 |
| 690 | 0.00000 | 310509 |
| 691 | 0.00000 | 310509 |
| 692 | 0.01695 | 310509 |
| 693 | 0.06780 | 310507 |
| 694 | 0.05085 | 310507 |
| 695 | 0.00000 | 310507 |
| 696 | 0.01695 | 310506 |
| 697 | 0.01695 | 310506 |
| 698 | 0.01695 | 310506 |
| 699 | 0.06780 | 310505 |
| 700 | 0.11864 | 310502 |
| 701 | 0.03390 | 310502 |
| 702 | 0.05085 | 310502 |
| 703 | 0.08475 | 310501 |
| 704 | 0.05085 | 310500 |
| 705 | 0.00000 | 310499 |
| 706 | 0.01695 | 310497 |
| 707 | 0.01695 | 310497 |
| 708 | 0.08621 | 310493 |
| 709 | 0.01724 | 310493 |
| 710 | 0.01724 | 310492 |
| 711 | 0.01724 | 310491 |
| 712 | 0.03448 | 310488 |
| 713 | 0.03448 | 310488 |
| 714 | 0.05085 | 310487 |
| 715 | 0.03390 | 310485 |
| 716 | 0.01695 | 310484 |
| 717 | 0.05085 | 310484 |
| 718 | 0.01695 | 310481 |
| 719 | 0.01695 | 310481 |
| 720 | 0.05085 | 310480 |
| 721 | 0.00000 | 310477 |
| 722 | 0.01695 | 310476 |
| 723 | 0.00000 | 310473 |
| 724 | 0.03390 | 310453 |
| 725 | 0.03390 | 310445 |
| 726 | 0.00000 | 310443 |
| 727 | 0.01695 | 310442 |
| 728 | 0.00000 | 310419 |
| 729 | 0.00000 | 310411 |
| 730 | 0.00000 | 310411 |
| 731 | 0.03448 | 310411 |
| 732 | 0.01724 | 310386 |
| 733 | 0.03448 | 310373 |
| 734 | 0.00000 | 310339 |
| 735 | 0.00000 | 310337 |
| 736 | 0.05263 | 310323 |
| 737 | 0.05263 | 310316 |
| 738 | 0.03509 | 310301 |
| 739 | 0.01754 | 310296 |
| 740 | 0.00000 | 310287 |
| 741 | 0.01754 | 310216 |
| 742 | 0.03571 | 310189 |
| 743 | 0.00000 | 310100 |
| 744 | 0.00000 | 310072 |
| 745 | 0.00000 | 309988 |
| 746 | 0.00000 | 210688 |
| 747 | 0.00000 | 210354 |
| 748 | 0.00000 | 210314 |
| 749 | 0.00000 | 210304 |
| 750 | 0.04000 | 210290 |
| 751 | 0.16000 | 210288 |
| 752 | 0.00000 | 210287 |
| 753 | 0.00000 | 210287 |
| 754 | 0.00000 | 210287 |
| 755 | 0.00000 | 210287 |
| 756 | 0.08000 | 210287 |
| 757 | 0.00000 | 210287 |
| 758 | 0.04000 | 210287 |
| 759 | 0.04000 | 210288 |
| 760 | 0.00000 | 210288 |
| 761 | 0.03846 | 210288 |
| 762 | 0.03846 | 210288 |
| 763 | 0.00000 | 210288 |
| 764 | 0.07692 | 210288 |
| 765 | 0.00000 | 210287 |
| 766 | 0.07692 | 210287 |
| 767 | 0.00000 | 210287 |
| 768 | 0.00000 | 210288 |
| 769 | 0.00000 | 210316 |
| 770 | 0.00000 | 210335 |
| 771 | 0.00000 | 210335 |
| 772 | 0.00000 | 345490 |
| 773 | 0.00000 | 345496 |
| 774 | 0.00026 | 345496 |
| 775 | 0.02145 | 345526 |
| 776 | 0.00659 | 345533 |
| 777 | 0.02776 | 345548 |
| 778 | 0.01020 | 345623 |
| 779 | 0.00856 | 345626 |
| 780 | 0.01119 | 345647 |
| 781 | 0.01219 | 345654 |
| 782 | 0.03577 | 345654 |
| 783 | 0.00978 | 345655 |
| 784 | 0.01367 | 345668 |
| 785 | 0.01419 | 345670 |
| 786 | 0.03284 | 345674 |
| 787 | 0.06407 | 345681 |
| 788 | 0.04466 | 345685 |
| 789 | 0.01076 | 345689 |
| 790 | 0.01589 | 345691 |
| 791 | 0.02766 | 345789 |
| 792 | 0.04118 | 345803 |
| 793 | 0.04267 | 345803 |
| 794 | 0.01176 | 345806 |
| 795 | 0.01076 | 345807 |
| 796 | 0.04366 | 345812 |
| 797 | 0.01939 | 345815 |
| 798 | 0.01189 | 345832 |
| 799 | 0.07651 | 345846 |
| 800 | 0.02642 | 346074 |
| 801 | 0.01652 | 346074 |
| 802 | 0.02979 | 346081 |
| 803 | 0.04895 | 346081 |
| 804 | 0.07605 | 346088 |
| 805 | 0.01503 | 346090 |
| 806 | 0.02468 | 346093 |
| 807 | 0.01503 | 346098 |
| 808 | 0.02032 | 346099 |
| 809 | 0.01116 | 346099 |
| 810 | 0.06265 | 346099 |
| 811 | 0.02982 | 346100 |
| 812 | 0.02265 | 346100 |
| 813 | 0.02115 | 346100 |
| 814 | 0.03479 | 346100 |
| 815 | 0.02303 | 346100 |
| 816 | 0.01111 | 346103 |
| 817 | 0.02497 | 346104 |
| 818 | 0.01971 | 346104 |
| 819 | 0.01809 | 346104 |
| 820 | 0.03443 | 346105 |
| 821 | 0.03079 | 346106 |
| 822 | 0.01135 | 346107 |
| 823 | 0.04189 | 346107 |
| 824 | 0.04898 | 346107 |
| 825 | 0.01470 | 346107 |
| 826 | 0.01507 | 346107 |
| 827 | 0.01370 | 346107 |
| 828 | 0.03239 | 346107 |
| 829 | 0.01171 | 346107 |
| 830 | 0.01021 | 346107 |
| 831 | 0.02503 | 346107 |
| 832 | 0.03002 | 346106 |
| 833 | 0.02753 | 346106 |
| 834 | 0.01221 | 346105 |
| 835 | 0.01033 | 346104 |
| 836 | 0.04258 | 346102 |
| 837 | 0.02889 | 346102 |
| 838 | 0.01121 | 346102 |
| 839 | 0.01843 | 346102 |
| 840 | 0.01768 | 346097 |
| 841 | 0.03716 | 346095 |
| 842 | 0.02495 | 346096 |
| 843 | 0.01008 | 346096 |
| 844 | 0.00685 | 346095 |
| 845 | 0.00822 | 346095 |
| 846 | 0.01793 | 346094 |
| 847 | 0.00685 | 346094 |
| 848 | 0.01769 | 346093 |
| 849 | 0.01085 | 346091 |
| 850 | 0.00960 | 346090 |
| 851 | 0.00636 | 346090 |
| 852 | 0.01796 | 346082 |
| 853 | 0.01830 | 346071 |
| 854 | 0.01208 | 346064 |
| 855 | 0.00336 | 346039 |
| 856 | 0.01046 | 345991 |
| 857 | 0.00697 | 345989 |
| 858 | 0.00909 | 345984 |
| 859 | 0.01717 | 345984 |
| 860 | 0.02315 | 345982 |
| 861 | 0.01630 | 345962 |
| 862 | 0.00722 | 345960 |
| 863 | 0.00846 | 345957 |
| 864 | 0.00846 | 345947 |
| 865 | 0.01468 | 345936 |
| 866 | 0.00199 | 345927 |
| 867 | 0.01618 | 345884 |
| 868 | 0.01233 | 345539 |
| 869 | 0.00398 | 136448 |
| 870 | 0.00486 | 135825 |
| 871 | 0.00699 | 135824 |
| 872 | 0.00312 | 135823 |
| 873 | 0.00462 | 135823 |
| 874 | 0.00337 | 135822 |
| 875 | 0.00474 | 135822 |
| 876 | 0.00175 | 135822 |
| 877 | 0.00612 | 135822 |
| 878 | 0.01123 | 135822 |
| 879 | 0.00449 | 135822 |
| 880 | 0.01498 | 135822 |
| 881 | 0.01585 | 135822 |
| 882 | 0.00923 | 135822 |
| 883 | 0.03270 | 135822 |
| 884 | 0.00175 | 135822 |
| 885 | 0.01710 | 135822 |
| 886 | 0.00512 | 135822 |
| 887 | 0.00799 | 135822 |
| 888 | 0.00537 | 135822 |
| 889 | 0.00400 | 135822 |
| 890 | 0.00688 | 135822 |
| 891 | 0.00438 | 135822 |
| 892 | 0.00263 | 135822 |
| 893 | 0.01688 | 135822 |
| 894 | 0.00574 | 135822 |
| 895 | 0.00200 | 135822 |
| 896 | 0.00262 | 135822 |
| 897 | 0.00861 | 135822 |
| 898 | 0.00112 | 135822 |
| 899 | 0.01173 | 135822 |
| 900 | 0.00187 | 135822 |
| 901 | 0.00587 | 135822 |
| 902 | 0.01260 | 135822 |
| 903 | 0.00574 | 135822 |
| 904 | 0.00612 | 135822 |
| 905 | 0.00324 | 135822 |
| 906 | 0.00849 | 135822 |
| 907 | 0.00287 | 135822 |
| 908 | 0.00488 | 135822 |
| 909 | 0.00200 | 135822 |
| 910 | 0.00537 | 135822 |
| 911 | 0.00162 | 135822 |
| 912 | 0.00162 | 135822 |
| 913 | 0.00325 | 135822 |
| 914 | 0.00449 | 135822 |
| 915 | 0.00087 | 135822 |
| 916 | 0.00337 | 135822 |
| 917 | 0.00125 | 135822 |
| 918 | 0.00325 | 135822 |
| 919 | 0.00325 | 135822 |
| 920 | 0.00237 | 135822 |
| 921 | 0.00187 | 135822 |
| 922 | 0.00212 | 135822 |
| 923 | 0.00637 | 135822 |
| 924 | 0.00312 | 135822 |
| 925 | 0.00062 | 135822 |
| 926 | 0.00400 | 135822 |
| 927 | 0.00112 | 135822 |
| 928 | 0.00312 | 135822 |
| 929 | 0.01186 | 135822 |
| 930 | 0.00349 | 135822 |
| 931 | 0.01685 | 135822 |
| 932 | 0.01473 | 135822 |
| 933 | 0.00462 | 135822 |
| 934 | 0.00175 | 135822 |
| 935 | 0.00499 | 135822 |
| 936 | 0.00112 | 135822 |
| 937 | 0.00200 | 135822 |
| 938 | 0.00375 | 135822 |
| 939 | 0.00487 | 135822 |
| 940 | 0.00150 | 135822 |
| 941 | 0.00225 | 135822 |
| 942 | 0.00237 | 135822 |
| 943 | 0.00262 | 135822 |
| 944 | 0.00087 | 135822 |
| 945 | 0.00162 | 135822 |
| 946 | 0.00075 | 135822 |
| 947 | 0.00187 | 135822 |
| 948 | 0.00037 | 135822 |
| 949 | 0.00125 | 135822 |
| 950 | 0.00262 | 135822 |
| 951 | 0.00886 | 135822 |
| 952 | 0.00986 | 135822 |
| 953 | 0.00287 | 135822 |
| 954 | 0.00112 | 135822 |
| 955 | 0.00137 | 135822 |
| 956 | 0.00325 | 135822 |
| 957 | 0.00100 | 135822 |
| 958 | 0.00350 | 135822 |
| 959 | 0.00100 | 135822 |
| 960 | 0.00362 | 135822 |
| 961 | 0.00150 | 135822 |
| 962 | 0.00911 | 135822 |
| 963 | 0.00674 | 135822 |
| 964 | 0.00537 | 135822 |
| 965 | 0.00749 | 135822 |
| 966 | 0.00312 | 135822 |
| 967 | 0.00062 | 135822 |
| 968 | 0.00212 | 135822 |
| 969 | 0.00475 | 135822 |
| 970 | 0.00275 | 135822 |
| 971 | 0.00050 | 135822 |
| 972 | 0.00200 | 135822 |
| 973 | 0.00337 | 135822 |
| 974 | 0.00824 | 135822 |
| 975 | 0.00350 | 135822 |
| 976 | 0.00187 | 135822 |
| 977 | 0.00037 | 135822 |
| 978 | 0.00649 | 135822 |
| 979 | 0.00187 | 135822 |
| 980 | 0.00287 | 135822 |
| 981 | 0.00225 | 135822 |
| 982 | 0.00262 | 135822 |
| 983 | 0.00637 | 135822 |
| 984 | 0.00800 | 135822 |
| 985 | 0.00824 | 135822 |
| 986 | 0.00050 | 135822 |
| 987 | 0.00275 | 135822 |
| 988 | 0.00050 | 135822 |
| 989 | 0.00250 | 135822 |
| 990 | 0.00125 | 135822 |
| 991 | 0.00225 | 135822 |
| 992 | 0.00062 | 135822 |
| 993 | 0.00450 | 135822 |
| 994 | 0.00400 | 135822 |
| 995 | 0.00275 | 135822 |
| 996 | 0.00262 | 135822 |
| 997 | 0.00137 | 135822 |
| 998 | 0.00150 | 135822 |
| 999 | 0.00362 | 135822 |
| 1000 | 0.14646 | 135822 |
| 1001 | 0.00150 | 135822 |
| 1002 | 0.00587 | 135822 |
| 1003 | 0.00100 | 135822 |
| 1004 | 0.00387 | 135822 |
| 1005 | 0.00100 | 135822 |
| 1006 | 0.00653 | 135822 |
| 1007 | 0.00125 | 135822 |
| 1008 | 0.00176 | 135822 |
| 1009 | 0.00237 | 135822 |
| 1010 | 0.00375 | 135822 |
| 1011 | 0.00100 | 135822 |
| 1012 | 0.00037 | 135822 |
| 1013 | 0.00287 | 135822 |
| 1014 | 0.00112 | 135822 |
| 1015 | 0.00200 | 135822 |
| 1016 | 0.00374 | 135822 |
| 1017 | 0.00250 | 135822 |
| 1018 | 0.00162 | 135822 |
| 1019 | 0.00287 | 135822 |
| 1020 | 0.00874 | 135822 |
| 1021 | 0.00062 | 135822 |
| 1022 | 0.00175 | 135822 |
| 1023 | 0.00424 | 135822 |
| 1024 | 0.00374 | 135822 |
| 1025 | 0.00150 | 135822 |
| 1026 | 0.00250 | 135822 |
| 1027 | 0.00275 | 135822 |
| 1028 | 0.00162 | 135822 |
| 1029 | 0.00562 | 135822 |
| 1030 | 0.00137 | 135822 |
| 1031 | 0.00237 | 135822 |
| 1032 | 0.00200 | 135822 |
| 1033 | 0.00212 | 135822 |
| 1034 | 0.00624 | 135822 |
| 1035 | 0.00337 | 135822 |
| 1036 | 0.00162 | 135822 |
| 1037 | 0.00375 | 135822 |
| 1038 | 0.00137 | 135822 |
| 1039 | 0.00562 | 135822 |
| 1040 | 0.00325 | 135822 |
| 1041 | 0.00350 | 135822 |
| 1042 | 0.00225 | 135822 |
| 1043 | 0.00100 | 135822 |
| 1044 | 0.00850 | 135822 |
| 1045 | 0.00125 | 135822 |
| 1046 | 0.00551 | 135822 |
| 1047 | 0.00924 | 135822 |
| 1048 | 0.00452 | 135822 |
| 1049 | 0.08995 | 135822 |
| 1050 | 0.08853 | 135822 |
| 1051 | 0.01609 | 135822 |
| 1052 | 0.02608 | 135822 |
| 1053 | 0.00354 | 135822 |
| 1054 | 0.12916 | 135822 |
| 1055 | 0.10200 | 135822 |
| 1056 | 0.05403 | 135822 |
| 1057 | 0.05892 | 135822 |
| 1058 | 0.03645 | 135822 |
| 1059 | 0.09903 | 135822 |
| 1060 | 0.03593 | 135822 |
| 1061 | 0.05500 | 135822 |
| 1062 | 0.05196 | 135822 |
| 1063 | 0.01266 | 135822 |
| 1064 | 0.05611 | 135822 |
| 1065 | 0.03533 | 135822 |
| 1066 | 0.04076 | 135822 |
| 1067 | 0.08764 | 135822 |
| 1068 | 0.02532 | 135822 |
| 1069 | 0.03531 | 135822 |
| 1070 | 0.00978 | 135822 |
| 1071 | 0.05832 | 135822 |
| 1072 | 0.01367 | 135822 |
| 1073 | 0.07451 | 135822 |
| 1074 | 0.04464 | 135822 |
| 1075 | 0.03900 | 135822 |
| 1076 | 0.05690 | 135822 |
| 1077 | 0.06381 | 135822 |
| 1078 | 0.04296 | 135822 |
| 1079 | 0.03793 | 135822 |
| 1080 | 0.02627 | 135822 |
| 1081 | 0.04760 | 135822 |
| 1082 | 0.08349 | 135822 |
| 1083 | 0.12176 | 135822 |
| 1084 | 0.05731 | 135822 |
| 1085 | 0.10111 | 135822 |
| 1086 | 0.09657 | 135822 |
| 1087 | 0.04234 | 135822 |
| 1088 | 0.00954 | 135822 |
| 1089 | 0.14889 | 135822 |
| 1090 | 0.12841 | 135822 |
| 1091 | 0.02152 | 135822 |
| 1092 | 0.03911 | 135822 |
| 1093 | 0.07709 | 135822 |
| 1094 | 0.03921 | 135822 |
| 1095 | 0.01688 | 135822 |
| 1096 | 0.06032 | 135822 |
| 1097 | 0.02708 | 135822 |
| 1098 | 0.04602 | 135822 |
| 1099 | 0.01891 | 135822 |
| 1100 | 0.14183 | 135822 |
| 1101 | 0.02572 | 135822 |
| 1102 | 0.03984 | 135822 |
| 1103 | 0.08522 | 135822 |
| 1104 | 0.00968 | 135822 |
| 1105 | 0.04414 | 135822 |
| 1106 | 0.04719 | 135822 |
| 1107 | 0.01005 | 135822 |
| 1108 | 0.04302 | 135822 |
| 1109 | 0.02894 | 135822 |
| 1110 | 0.07436 | 135822 |
| 1111 | 0.06585 | 135822 |
| 1112 | 0.03086 | 135822 |
| 1113 | 0.17156 | 135822 |
| 1114 | 0.11911 | 135822 |
| 1115 | 0.10506 | 135822 |
| 1116 | 0.03653 | 135822 |
| 1117 | 0.02021 | 135822 |
| 1118 | 0.03074 | 135822 |
| 1119 | 0.01982 | 135822 |
| 1120 | 0.03993 | 135822 |
| 1121 | 0.01733 | 135822 |
| 1122 | 0.05173 | 135822 |
| 1123 | 0.07741 | 135822 |
| 1124 | 0.06795 | 135822 |
| 1125 | 0.01948 | 135822 |
| 1126 | 0.09251 | 135822 |
| 1127 | 0.04115 | 135822 |
| 1128 | 0.03972 | 135822 |
| 1129 | 0.01423 | 135821 |
| 1130 | 0.07186 | 135820 |
| 1131 | 0.05201 | 135820 |
| 1132 | 0.08749 | 135820 |
| 1133 | 0.14158 | 135817 |
| 1134 | 0.10097 | 135817 |
| 1135 | 0.05251 | 135817 |
| 1136 | 0.18975 | 135816 |
| 1137 | 0.07140 | 135815 |
| 1138 | 0.11156 | 135815 |
| 1139 | 0.01208 | 135813 |
| 1140 | 0.01560 | 135811 |
| 1141 | 0.06641 | 135808 |
| 1142 | 0.01331 | 135803 |
| 1143 | 0.01946 | 135786 |
| 1144 | 0.02786 | 135774 |
| 1145 | 0.03102 | 135773 |
| 1146 | 0.06602 | 135771 |
| 1147 | 0.02779 | 135768 |
| 1148 | 0.08915 | 135765 |
| 1149 | 0.01097 | 135763 |
| 1150 | 0.02080 | 135762 |
| 1151 | 0.02459 | 135760 |
| 1152 | 0.04074 | 135717 |
| 1153 | 0.07076 | 135646 |
| 1154 | 0.09738 | 135632 |
| 1155 | 0.02465 | 135625 |
| 1156 | 0.05586 | 135612 |
| 1157 | 0.06397 | 135607 |
| 1158 | 0.02250 | 135595 |
| 1159 | 0.04661 | 135592 |
| 1160 | 0.07147 | 135582 |
| 1161 | 0.01482 | 135553 |
| 1162 | 0.04712 | 135540 |
| 1163 | 0.01788 | 135525 |
| 1164 | 0.08558 | 135468 |
| 1165 | 0.06680 | 135439 |
| 1166 | 0.05682 | 135420 |
| 1167 | 0.08234 | 135408 |
| 1168 | 0.04539 | 135387 |
| 1169 | 0.04794 | 135369 |
| 1170 | 0.08898 | 135349 |
| 1171 | 0.02802 | 135341 |
| 1172 | 0.04505 | 135298 |
| 1173 | 0.10562 | 135195 |
| 1174 | 0.10816 | 134971 |
| 1175 | 0.06300 | 134898 |
| 1176 | 0.08480 | 134853 |
| 1177 | 0.05258 | 134773 |
| 1178 | 0.02578 | 134745 |
| 1179 | 0.11942 | 134684 |
| 1180 | 0.00741 | 134662 |
| 1181 | 0.00869 | 134431 |
| 1182 | 0.01801 | 133597 |
| 1183 | 0.00031 | 135553 |
| 1184 | 0.00000 | 135540 |
